# Safety and feasibility of apheresis to harvest and concentrate parasites from subjects with induced blood stage *Plasmodium vivax* infection

**DOI:** 10.1186/s12936-021-03581-w

**Published:** 2021-01-14

**Authors:** Anand Odedra, Kari Mudie, Glen Kennedy, Rebecca E. Watts, Emilie Rossignol, Hayley Mitchell, Jeremy Gower, Maria Rebelo, Zuleima Pava, Rebecca Pawliw, Stephen Woolley, David G. Lalloo, Greg Robinson, Sean Lynch, Katharine A. Collins, Fiona Amante, James McCarthy

**Affiliations:** 1 QIMR Berghofer Medical Research Institute, Herston Road, Brisbane, QLD 4006 Australia; 2 Liverpool School of Tropical Medicine, Pembroke Place, Liverpool, L3 5QA UK; 3 Haematology Department, Royal Brisbane and Women’s Hospital, Butterfield Street, Herston, Brisbane, QLD 4029 Australia; 4 Department of Medical Microbiology, Radboud University Medical Centre, Geert Grooteplein Zuid 10, 6525 GA Nijmegen, The Netherlands

**Keywords:** Malaria, Plasmodium, Apheresis, Parasite, Concentration

## Abstract

**Background:**

In the absence of a method to culture *Plasmodium vivax*, the only way to source parasites is ex vivo. This hampers many aspects of *P. vivax* research. This study aimed to assess the safety of apheresis, a method for selective removal of specific components of blood as a means of extracting and concentrating *P. vivax* parasites.

**Methods:**

An iterative approach was employed across four non-immune healthy human subjects in single subject cohorts. All four subjects were inoculated with ~ 564 blood stage *P. vivax* (HMP013-*Pv*) and subjected to apheresis 10 to 11 days later*.* Blood samples collected during apheresis (haematocrit layers 0.5% to 11%) were tested for the presence and concentration of *P. vivax* by microscopy, flow cytometry, 18S rDNA qPCR for total parasites, and *pvs2*5 qRT-PCR for female gametocyte transcripts. Safety was determined by monitoring adverse events. Malaria transmission to mosquitoes was assessed by membrane feeding assays.

**Results:**

There were no serious adverse events and no significant safety concerns. Apheresis concentrated asexual parasites by up to 4.9-fold (range: 0.9–4.9-fold) and gametocytes by up to 1.45-fold (range: 0.38–1.45-fold) compared to pre-apheresis densities. No single haematocrit layer contained > 40% of all the recovered *P. vivax* asexual parasites. Ex vivo concentration of parasites by Percoll gradient centrifugation of whole blood achieved greater concentration of gametocytes than apheresis. Mosquito transmission was enhanced by up to fivefold in a single apheresis sample compared to pre-apheresis.

**Conclusion:**

The modest level of parasite concentration suggests that the use of apheresis may not be an ideal method for harvesting *P. vivax.*

*Trial Registration* Australia New Zealand Clinical Trials Registry (ANZCTR) Trial ID: ACTRN12617001502325 registered on 19th October 2017. https://www.anzctr.org.au/Trial/Registration/TrialReview.aspx?id=373812.

## Background

*Plasmodium falciparum* is the most prevalent malaria parasite in Africa. However, *Plasmodium vivax* has a wider geographical distribution. In 2018, there were approximately 75 million cases of malaria due to *P. vivax*, accounting for 50% of cases in South East Asia and 75% of cases in the Americas [1].

In the absence of a method for continuous in vitro culture of *P. vivax*, parasites are usually sourced ex vivo from infected humans. This limits many aspects of the study of *P. vivax* research, including the development of interventions to control and eliminate *P. vivax*, such as diagnostics, drugs and vaccines. For example, a reliable source of *P. vivax* sporozoites is required to test and develop new drugs targeting the dormant liver-stage parasites—the hypnozoites. Currently this entails an expensive, logistically complex and unreliable process of sourcing *P. vivax*-infected mosquitoes from endemic areas. In addition to the practical issues, parasites sourced in this way are not genetically homogenous. Thus, experiments are subject to possible effects of strain variability.

Experimental infection of human subjects with malaria termed volunteer infection studies (VIS) or controlled human malaria infection (CHMI) studies are increasingly being used for drug and vaccine development [2–7]. Infections can be induced by mosquito bite, injection of sporozoites or blood-stage parasites. The latter is used in the induced blood stage malaria (IBSM) model, where healthy subjects are injected with *Plasmodium* infected red blood cells. The IBSM model is being increasingly used for clinical studies of *P. vivax* [7–9].

It has recently been shown that *P. vivax* can be successfully transmitted from healthy subjects to *Anopheles stephensi* mosquitoes [9, 10]. In this study sporozoites were harvested from the infected mosquitoes that were able to infect human hepatocytes in vitro [9]. Although this system offers the potential to study the biology of *P. vivax* malaria transmission and liver stage parasites, it is not a sustainable large-scale source of sporozoites for downstream work.

Apheresis is the removal of a specific component of an individual’s blood. Currently, centrifugal apheresis is the preferred method whereby blood components are separated based on buoyancy. Computer-controlled automated apheresis systems undertake continuous removal, separation of the target component, and then return the remaining blood to the individual [11]. Automated erythrocytapheresis, also known as red cell exchange (RCE) has been used in the past for treatment of severe *P. falciparum* malaria with the rationale of reducing the parasitized red blood cell concentration by replacing *Plasmodium*-infected red blood cells with normal donor red blood cells [12]. However, the rapid parasite clearance resulting from artesunate therapy has negated the need for RCE as a treatment for severe malaria [13, 14].

Reported below are the results of a *P. vivax* clinical trial where apheresis was used as a means to harvest and concentrate all blood stages of *P. vivax,* including gametocytes, from human subjects experimentally infected with blood-stage *P. vivax* parasites.

## Methods

### Study design

The study presented here is a Phase 1 exploratory study that was conducted in four sequential single subject cohorts (ANZCTR Trial ID: ACTRN12617001502325) and performed at Q-Pharm Pty Ltd, Brisbane, Australia and the Apheresis Unit at the Royal Brisbane and Women’s Hospital (RBWH), Australia between October 2017 and May 2019. The primary objective of the study was to determine the safety of the *P. vivax* infection in healthy subjects following inoculation with blood-stage parasites, and the safety of apheresis for collection of *P. vivax* parasites from experimentally infected subjects. Secondary objectives were to assess the feasibility of apheresis as a method of harvesting, concentrating and subsequently cryopreserving *P. vivax* parasites from healthy subjects. Exploratory objectives were to evaluate the potential for apheresis to be used as a method for producing a *P. vivax* human malaria parasite bank, to evaluate the transmission of *P. vivax* gametocytes to mosquitoes and to collect and store plasma and peripheral blood mononuclear cells harvested using apheresis.

Specific modifications to the study protocol, such as the apheresis procedure, were required between subjects in an attempt to optimize the procedure and meet the objectives. All changes made between subjects were based on the findings from previous subjects.

### Study subjects

Healthy adult males and females, aged between 18 and 55 years who met all inclusion criteria and none of the exclusion criteria were eligible for participation. Subjects were required to be malaria-naïve, Duffy blood group positive and have blood type O. Female subjects had to be Rh(D) positive. All subjects had to be available for a safety follow up period of three months. A full list of the inclusion/exclusion criteria for this study is included in the study protocol located in Additional file 1.

### Study conduct

#### Pre-clinical component

A pre-clinical experiment was conducted prior to the clinical trial in order to confirm the feasibility of harvesting *Plasmodium* parasites using apheresis. The *P. falciparum* NF54 clone was used in these experiments [15] due to limited availability of *P. vivax* parasites. *Plasmodium. falciparum* infected red blood cells (17.6 ml; 16 ml blood with 0.1% asexual parasitaemia and 1.67 ml blood with 0.01% gametocytaemia) were added to 450 ml of fresh venous whole blood and subjected to ex vivo apheresis. Samples were collected from the 1%, 2%, 3%, 5% and 7% haematocrit (HCT) layers as determined by visualizing the colour saturation of the apheresis product. An automated haematology analyser (Sysmex XN-3000; Sysmex UK) was used retrospectively to confirm the HCT of samples collected during apheresis. Presence of parasites was assessed in each layer by 18S qPCR [16] and microscopy.

#### Clinical component

Following intravenous injection of *P. vivax* (day 0), subjects were monitored by daily telephone calls until day 4, when subjects visited the clinical unit daily until the day of apheresis. Subjects were monitored for adverse events (AEs), signs and symptoms of malaria infection, and blood was collected for 18S qPCR measurement of parasitaemia. The severity of AEs were determined by the common terminology of clinical trial adverse events (CTCAE) v. 4.03 [17].

The threshold for commencement of apheresis and treatment with artemether–lumefantrine was within 24 h of a parasitaemia > 20,000 parasites/mL, or the Malaria Clinical Score reaching > 6 [10], or at the Investigator’s discretion. The morning that this threshold was reached (anticipated based on previous studies to be Day 10 or 11 [9], subjects were admitted to the clinical unit (Q-Pharm) for initial safety assessments before being escorted to the Apheresis Unit at RBWH by Q-Pharm staff. The Apheresis Unit is located in the Haematology Department at RBWH where patients are subject to donor or therapeutic apheresis. At the Apheresis Unit the subjects underwent the apheresis procedure as per the Standard Operating Procedure (Additional files 2, 3, 4 and 5) whilst being supervised by the apheresis specialist nurse and under the supervision of the responsible clinical haematologist (GK). The same apheresis nurse performed the apheresis procedure for all four subjects. The apheresis procedure lasted 1–4 h. Subjects were then escorted back to the clinical unit and began treatment with artemether–lumefantrine (Riamet®, Novartis Pharmaceuticals Australia Pty Ltd). Treatment consisted of six doses of 4 tablets at 12 hourly intervals (each tablet contains 20 mg artemether and 120 mg of lumefantrine). Subjects remained confined within the clinical unit for 48–72 h for safety monitoring. Following release from confinement, subjects attended protocol specified visits until three months post-treatment to monitor for signs of recrudescent parasitaemia and to assess late safety signals. Relapse is not a concern in the *P. vivax* IBSM studies as liver infection is bypassed and hypnozoites are not produced. A schematic of the study design is shown in Additional file 6: Fig. S1.

This study used an iterative adaptive design approach where subject safety and outcome data were analysed after each subject and modifications made to improve the chances of meeting the exploratory objectives in the subsequent subject. A summary of the changes instituted is shown in Table 1.

**Table 1:** Summary of main study design differences between subjects

	Subject 1	Subject 2	Subject 3	Subject 4^#^
Apheresis procedure	CMNC	CMNC	CMNC	Red cell depletion followed by CMNC on red cell depletion product
HCT layers sampled*	1%, 2%, 3%, 5%, 7%	1%, 2%, 3%, 5%, 7%	0.5%, 1%, 2%, 3%, 5%, 7%, 11%^+^ 2–3%, 5–7%, 1–7%	From the primary apheresis (HCT): Intermediate (64%) From the secondary apheresis (HCT): Final (3%) Spare (5%) Waste (42%)
Apheresis timepoint	10	10	11 PM	11 AM
Mosquito feeding assay samples	Pre-apheresis (with-Percoll enrichment), 1%, 2%, 3% HCT layers	Pre-apheresis (with-Percoll enrichment)	Pre-apheresis (with and without-Percoll enrichment)	Pre-apheresis (without-Percoll enrichment), Intermediate, Final, Waste
Whole blood:citrate ratio during apheresis	15:1	8:1	8:1	13:1
Citrate added to apheresis collection bags	No	Yes	Yes	Yes
Biological duplicates*	No	No	Yes	Yes

CMNC; continuous mononuclear cell collection, HCT; haematocrit. Protocols for subjects 1 to 4 and all experiments can be found in Additional files 7, 8, 9 and 10. *Biological duplicates involved repeat 18S qPCR testing from two separate blood samples from each HCT layer collected using apheresis.

When a HCT range is included the sample was taken from multiple HCT layers e.g. 5–7% = 5%, 6% and 7% HCT. ^+^Originally aimed to sample 8% HCT layer but actual sample consisted of 11% HCT. ^#^During cohort 4 a red cell depletion was carried out, producing an intermediate bag sample, followed by a second apheresis procedure on the red cell depletion product. The second apheresis procedure involved sampling of ~ 100 ml of the lowest HCT layers of the sample (final bag) followed by ~ 100mls of the subsequent lowest HCT layers (spare bag) and then the remainder ~ 100mls (waste bag).

### Malaria challenge agent

The *P. vivax* human malaria parasite (HMP) bank HMP013 was derived from blood group O rhesus positive blood donated from a returned traveller from India who presented with clinical manifestations of malaria [9]. The inoculum was prepared as previously described [18].

### Measurement of parasitaemia by qPCR

Parasitaemia was quantified using 18S qPCR targeting the highly conserved *Plasmodium* 18S ribosomal RNA gene [16, 19]. Quantitative reverse transcriptase PCR (qRT-PCR) assays were used to quantify gametocyte levels with assays targeting the *P. falciparum pfs25* (female) and *pfMGET* (male) gametocyte mRNA transcripts [20] and *P. vivax pvs2*5 (female) gametocyte mRNA transcripts [21].

### Flow cytometry

Flow cytometry was performed to characterize cell populations present in samples collected during the apheresis process. A combination of stains and antibodies were used to identify cells containing DNA/RNA (SYBR Green I), white blood cells (WBCs) (CD45 antibody) and/or reticulocytes (CD71 antibody). Samples from subject 1 were stained with SYBR Green I (Molecular Probes); samples from subjects 2 and 3 were stained with SYBR Green I and CD45-PacificBlue; and samples from subject 4 were stained with SYBR Green I, CD45-Pacific Blue and/or CD71-APC. Samples were kept on ice or at 4–8 °C until analysed by flow cytometry.

#### SYBR Green I staining

A volume of 2.5 μl or 1 × 10^6^ cells from each sample was stained with 30–50 μl of SYBR Green I at 10× for 30 min in the dark. After incubation, 200 μl of FACS buffer (2% fetal bovine serum in phosphate buffered saline) was added.

#### Antibody staining

Approximately 1 × 10^6^ cells were stained with 5–10 μg/ml of CD45-Pacific Blue or 2.5 μl of CD71 stock solution for 30 min at 4–8 °C in the dark. Cells were washed twice with PBS by centrifugation at 1455×*g* for 4 min, at 4 °C. After the last wash 200 μl of FACS buffer was added to the cells.

Double staining with SYBR Green I and CD45-Pacific Blue: A volume of 30 μl of SYBR Green I at 10× was added to pelleted cells that were previously stained with CD45-Pacific Blue (as mentioned above) for 30 min at 4–8 °C, in the dark. After incubation a volume of 200 μl of FACS buffer was added.

Triple staining with SYBR Green I, CD45-Pacific Blue and CD71-APC: Approximately 1 × 10^6^ cells were stained with 10 μg/ml of CD45-Pacific Blue, 2.5 μl of CD71 stock solution and 30 μl of SYBR Green I at 10x. Samples were incubated for 30 min in the fridge (4–8 °C) in the dark. Cells were washed twice with PBS by centrifugation at 1455×*g* for 4 min, at 4ºC. After incubation a volume of 200 μl of FACS buffer was added.

#### Flow cytometry analysis

Samples from subjects 1, 2 and 3 were acquired on a FACS CANTO II (BD Biosciences), using the 488 nm and 405 nm lasers. SYBR Green I positive cells were detected using a 530/30 nm band-pass filter and CD45-Pacific Blue positive cells were detected using a 450/50 nm band-pass filter. Samples from subject 4 were acquired on a LSR FORTESSA (BD Biosciences), using the 488 nm, 640 nm and 405 nm lasers. SYBR Green I positive cells were detected using a 530/30 nm filter, CD45-Pacific Blue positive cells were detected using a 450/50 nm filter and CD71-APC positive cells were detected using a 670/14 nm filter. Flow cytometry data was analysed using FlowJo® software (version 10.8, Tree Star Inc, Oregon, USA).

### Microscopy

Thick and thin smears were stained with Giemsa and examined under a 100× oil immersion objective by level 1 or 2 WHO certified malaria microscopists. Apheresis samples were expected to have a significantly different composition in terms of proportions of RBCs and WBCs when compared to whole blood (e.g. RBCs make up 1% and approximately 46% of 1% HCT and whole blood samples respectively). As such, standard parasitaemia measures by microscopy were not feasible. It was decided that the expert microscopists would estimate parasitaemia based on sample composition.

### Mosquito feeding assays

Transmissibility of pre-apheresis samples and post-apheresis samples to *An. stephensi* was evaluated using membrane feeding assays (MFA) [9, 22]. For enriched MFA, gametocytes present in 80 mL of whole blood (pre-apheresis) were enriched in 70% Percoll gradient. For direct MFA (DMFA), 650µL of pellet from whole blood (pre-apheresis) or from each apheresis sample was reconstituted to 50% haematocrit with malaria naïve AB + serum. Infection in midguts was assessed by qPCR [23] 8 days after the feeding assays. For logistic reasons, enriched MFA was not carried out in subject 4. Following consideration of gametocyte levels measured by qRT-PCR targeting *pvs25*, DMFA was not carried out in subject 2.

### Apheresis procedures

Apheresis was carried out using a Spectra Optia v11.3 apheresis system (Terumo BCT, Inc Tokyo Japan) as detailed in the Additional file 11.

The continuous mononuclear cell collection procedure was used to sample from the targeted HCT layers from the blood of subjects 1 to 3. The targeted HCT layers that were sampled from these subjects ranged from the platelet rich layer through to the red cell rich layer.

A double stage procedure was used to collect the targeted HCT layers from subject 4. In the first stage, a red cell depletion procedure was used to collect approximately 500 ml of packed red blood cells from the subject. Targeted HCT layers were then collected from the red cell concentrate using the polymorphonuclear (PMN) collection procedure. The starting product (red cell concentrate) for the second stage of this procedure had a significantly higher HCT than the whole blood of subjects 1 to 3 and sampling focussed on the higher HCT layers. This was the rationale for using a PMN collection in subject 4 rather than the CMNC collection used in subjects 1 to 3.

### Statistical analysis

Continuous data was summarized using descriptive statistics (mean and standard deviation, or median and interquartile range). Categorical data was presented using N and %. Descriptive statistics were produced using Microsoft Excel® (version 1903). GraphPad® Prism was used for the construction of all figures.

## Results

### Pre-clinical experiment

The feasibility of extracting *Plasmodium* parasites from blood using apheresis was initially assessed using cultured *P. falciparum* parasites. The 1% HCT layer contained the greatest concentration of all parasites as determined by 18S qPCR, with a 1.3-fold concentration of all parasites and a 3.7 and eightfold concentration for female and male gametocytes, compared to pre-apheresis (Additional file 6: Fig. S5 and Tables 1, 2 and 3). The 2% HCT layer contained the greatest concentration of asexual parasites by microscopy (2.7-fold concentration; Additional file 6: Fig. S6A and Table S4). The highest concentration of gametocytes detected by microscopy was seen in samples collected from the 1% HCT layer (76-fold; Additional file 6: Fig. S6A and Table S4) with stage 1 gametocytes making up the largest proportion (37.5%). All stages of gametocytes apart from stage 5 gametocytes were visualized. Both asexual parasites and gametocytes were also visualized by microscopy in the 1%, 3% and 5% HCT layers (Additional file 6: Fig. S6B and Table S5). These results demonstrated the technical feasibility of the approach and the experiment was allowed to proceed to the clinical stage.

**Table 2:** Summary of the main safety findings

	Subject 1	Subject 2	Subject 3	Subject 4	Total AEs
SAEs	0	0	0	0	0
AEs	20	13	15	20	68
AEs related to malaria	13	8	14	19	54
AEs related to apheresis	2	3	0	4	9
Max temp °C	40.2	38.8	40.2	39.6	N/A
Max malaria clinical score	8	1	2	7	N/A
Ibuprofen use	400 mg × 5	400 mg × 5	400 mg × 5	nil	N/A
Acetaminophen use	1 g × 4; 500 mg × 1	1 g × 2	1 g × 4	500 mg × 2; 1 g × 2	N/A
Peak ALT (IU/L)	111	118	47	80	N/A
Peak AST (IU/L)	83	57	42	44	N/A
Platelet Nadir (× 10^9^/L)	119	98	99	75	N/A
Maximum drop in haemoglobin from baseline (g/L)	17	9	25	20	N/A
Lymphocyte Nadir (× 10^9^/L)	0.33	0.43	0.58	0.75	N/A
Peak parasitaemia (parasites/mL)	15,943	35,156	64,243	44,431	N/A

*SAE* serious adverse event, *AE* adverse event

Summary of the main safety findings encountered during the study

**Table 3:** Mosquito infection rates following membrane feeding assays

Sample	Subject 1				Subject 2	Subject 3		Subject 4			
	Pre-apheresis (with-Percoll enrichment)	Apheresis samples			Pre-apheresis (with-Percoll enrichment)	Pre-apheresis (without-Percoll enrichment)	Pre-apheresis (with-Percoll enrichment)	^a^Pre-apheresis (without-Percoll enrichment)	Apheresis samples		
		1% HCT	2% HCT	3% HCT					Intermediate (64% HCT)	Final (3% HCT)	Waste (42% HCT)
Feeding rate (No. mosquitos fed/No. total mosquitos [%])	127/133 [95.5%]	101/103 [98.1%]	103/104 [99%]	114/116 [98.3%]	75/77 [97.4%]	102/103 [99%]	107/107 [100%]	29/29 [100%]	28/29 [97%]	26/26 [100%]	30/30 [100%]
Mortality rate (No. dead mosquitos/No. total mosquitos [%])	23/133 [17.3%]	3/103 [2.9%]	6/104 [5.8%]	11/116 [9.5%]	7/77 [9.1%]	6/103 [5.8%]	5/107 [4.7%]	1/29 [3.4%]	3/29 [10.3%]	1/26 [3.8%]	1/30 [3.3%]
Infection rate (No. mosquitoes with oocysts/No. mosquitos tested [%])	0/110[0%]	0/105 [0%]	0/98 [0%]	0/105 [0%]	0/77 [0%]	16/93 [17.2%]	99/100 [99%]	1/28 [3.6%]	1/25 [4%]	5/25 [20%]	0/24 [0%]

Only subjects 1 and 4 involved the testing of samples collected using apheresis. For logistic reasons, MFA was not carried out in subject 4. Following consideration of gametocyte levels, DMFA was not carried out in subject 2. Reported parameters include feeding rate of mosquitoes, adult mosquito mortality rate after feeding and mosquito infection rate

^a^MFA without Percoll enrichment to save on blood volume draw for safety reasons

### Clinical experiment

**Fig. 1:**
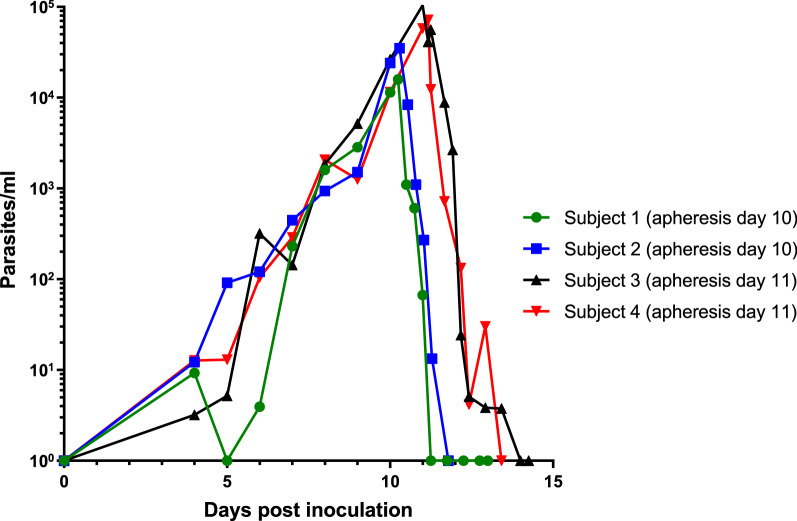
Parasite growth curves. Parasitaemia as measured by 18S qPCR in all four subjects. Day 0 represents the day of inoculation. Apheresis occurred on day 10 for subjects 1 and 2, and day 11 for subjects 3 and 4

The course of *P. vivax* infection (Fig. 1) followed the same course as demonstrated in previous studies [9]. Subjects 1 and 2 were treated with artemether/lumefantrine 10 days post malaria inoculation. To augment pre-apheresis parasitaemia, treatment of subjects 3 and 4 was delayed to day 11. Apheresis could be delayed safely as the clinical signs observed for subjects 3 and 4 were mild enough for the artemether/lumefantrine treatment, only administered after apheresis, to be postponed by 24 h. All subjects became 18S qPCR negative for parasites within 72 h of treatment initiation.

A summary of the key differences in the planning of the clinical trial in each of the four subject cohorts is shown in Table 1. During cohort 4 a red cell depletion was carried out, producing an intermediate bag sample, followed by a second apheresis procedure on the red cell depletion product. The second apheresis procedure involved sampling of ~ 100 ml of the lowest HCT layers of the sample (final bag) followed by 100mls of the subsequent lowest HCT layers (spare bag) and then the remainder (waste bag) (Additional file 6: Fig. S3). A schematic of the sampling that occurred during cohort 4 can be seen in Additional file 6: Fig. S3.

### Safety

A total of 68 AEs occurred in the 4 subjects (Table 2). No Serious AEs were reported. The majority of AEs were mild or moderate. Five severe AEs occurred in 4 subjects: one episode of neutropenia (0.68 × 10^9^/L [0.45 × LLN]; duration 8 days), two of lymphopenia (0.43 × 10^9^/L [0.43 × LLN] and 0.33 × 10^9^/L [0.33 × LLN]; both lasting 3 days), and two of fever (both 40.2 °C; duration 30 min and 25 min). All severe AEs were transient and resolved by the end of the study. The majority of AEs (54/68; 79.4%) were attributed to malaria, while 9/68 (13.2%) were attributed to apheresis. These included neutropenia (one subject nadir 0.68 × 10^9^/L) which was recorded three times due to changes in severity, two cases of lymphopenia (nadir 0.33 × 10^9^/L and 0.75 × 10^9^/L) and two cases of leukopenia (nadir 2.2 × 10^9^/L and 2.4 × 10^9^/L). One subject had an episode of herpes labialis (herpes simplex virus-1 PCR positive). One subject experienced mild hypophosphatemia (0.70 mmol/L) of two days duration. All AEs attributed to apheresis apart from the case of hypophosphataemia were considered to be possibly related to apheresis, malaria or a combination of both.

### Characteristics of samples collected by apheresis

The red blood cell counts in samples from the various HCT layers were generally in alignment with what would be expected (Additional file 6: Table S6), except in two subjects. In subject 2 the red blood cell counts in samples from the 2% HCT layer was closer to what would be expected from a 3% HCT layer and vice versa, and the sample from the 8% HCT sample in subject 3 had a HCT of 11%. The variability in expected HCT and actual HCT is a potential limitation and should be considered when designing any future studies. The cell composition of samples collected using apheresis in subjects 1 to 3 (Additional file 6: Fig. S7A) showed an ~ 60 to 170-fold decrease in the RBC:WBC ratio from pre-apheresis samples compared to apheresis samples. Among samples collected by apheresis from subject 4, where a double apheresis process was undertaken, the RBC:WBC ratio was close to that of the pre-apheresis sample (Additional file 6: Fig. S7B), with the exception of the final bag sample (3% HCT). Reticulocyte counts measured on the Sysmex analyser were the highest in subject 2: 0.23 × 10^9^/L (reference range for whole blood: 25–120 × 10^9^/L).

#### Concentration of asexual parasites

18S qPCR targets the highly conserved plasmodium 18S ribosomal RNA gene present in asexual parasites and gametocytes [14, 18]. However, based on the *P. vivax* life cycle, microscopy and *P. vivax* female gametocyte qRT PCR data (*pvs25*) it was determined that the vast majority of parasites, detected using 18S qPCR, were asexual parasites.

**Fig. 2:**
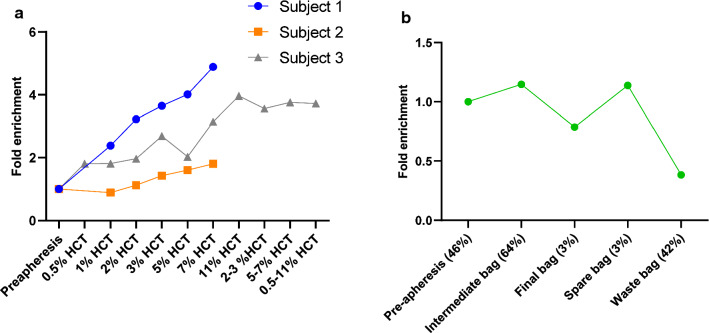
Fold enrichment of parasites/ml determined by 18S qPCR. Fold enrichment of parasites as determined by 18 qPCR in samples collected using apheresis compared to the pre-apheresis samples in subjects 1 to 3 (**a**) and subject 4 (**b**)

No single HCT layer contained > 40% of all the recovered *P. vivax* asexual parasites (Fig. 2). An increase in parasite concentration per ml of sample attained via apheresis occurred as HCT increased in subjects 1 to 3 (Fig. 2a), with some variation in relative enrichment of parasites in apheresis samples compared to pre-apheresis samples at any given HCT (Fig. 2a). The highest concentration achieved was a 4.9-fold increase in parasite density in the 7% HCT layer in subject 1 (Fig. 2a and Additional file 6: Table S7). There was no apparent enrichment of parasites when the procedure was modified to include a second apheresis process (subject 4; Fig. 2b).

When parasite concentration was adjusted for RBC count, all apheresis samples collected from subjects 1 to 3 demonstrated enrichment for asexual parasites compared to pre-apheresis (Additional file 6: Fig. S2A). In general, when parasite counts were corrected for RBC count, parasite enrichment was highest in the samples collected at the lower HCT (Additional file 6: Fig. S2 and Table S7). The relative concentration of parasites from subject 4, where the second apheresis procedure was performed, was observed in the lowest HCT samples. In particular, the final bag (3% HCT) and the spare bag (5% HCT) samples had relative enrichment levels of 20- and 8-fold respectively compared to pre-apheresis (Additional file 6: Fig. S2 and Table S7).

#### Concentration of P. vivax gametocytes

**Fig. 3:**
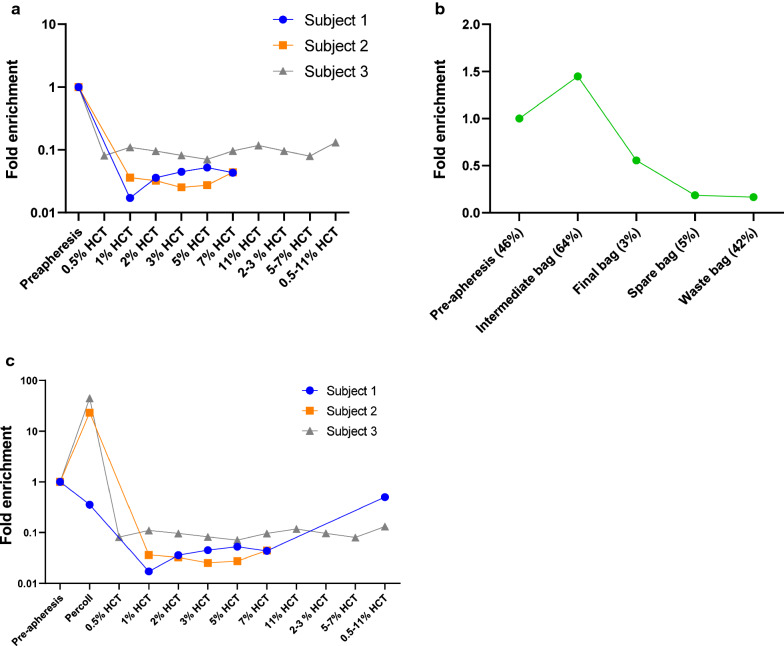
Fold enrichment female *P. vivax* gametocytes determined by *pvs25*/ml. Fold enrichment of female *P. vivax* gametocytes determined by *pvs25*/ml in samples collected using apheresis compared to the pre-apheresis sample in subjects 1, 2 and 3 (**a**) and subject 4 (**b**). Fold enrichment of female *P. vivax* gametocytes determined by *pvs25*/ml in samples collected using apheresis compared to the pre-apheresis sample and pre-apheresis samples enriched with Percoll in subjects 1, 2 and 3 (**c**)

Analysis of the apheresis samples from subject 4, where the double apheresis process was undertaken, demonstrated an increase in the level of female gametocytes of 1.45-fold per ml in the apheresis sample compared to a venous blood sample collected pre-apheresis, as determined by qRT PCR for the gametocyte-specific transcript *pvs25* (Fig. 3b and Additional file 6: Table S8).

Subjects 1, 2 and 3 demonstrated a reduction in the level of female gametocytes compared to pre-apheresis (Fig. 3a and Additional file 6: Table S8). In most cases the reduction was > 10-fold. Percoll concentration of whole blood taken pre-apheresis resulted in a significant enrichment (up to 45-fold in subject 3) of *pvs25* compared to pre-apheresis samples not enriched with Percoll (Fig. 3c and Additional file 6: Table S8).

In subject 1 the Percoll enrichment experiment failed due to technical issues resulting from suboptimal processing of the sample.

When gametocyte concentrations were corrected for red blood cell counts, enrichment levels were generally higher in the lower HCT samples (Additional file 6: Fig. S4a, b and Table S8). Relative enrichment of gametocytes in subjects 1, 2 and 3 was lower than for total parasites, with a maximum enrichment of 6.2-fold in the 0.5% HCT layer (Additional file 6: Table S8). In subjects 3 and 4 enrichment relative to red blood cell count was observed (Additional file 6: Fig. S4A, B). In subject 4, the greatest enrichment of gametocyte transcripts in the double apheresis process was observed in the spare bag (5% HCT) with an enrichment of 6.1-fold (Additional file 6: Fig. S4A, B and Table S8).

### Flow cytometry

**Fig. 4:**
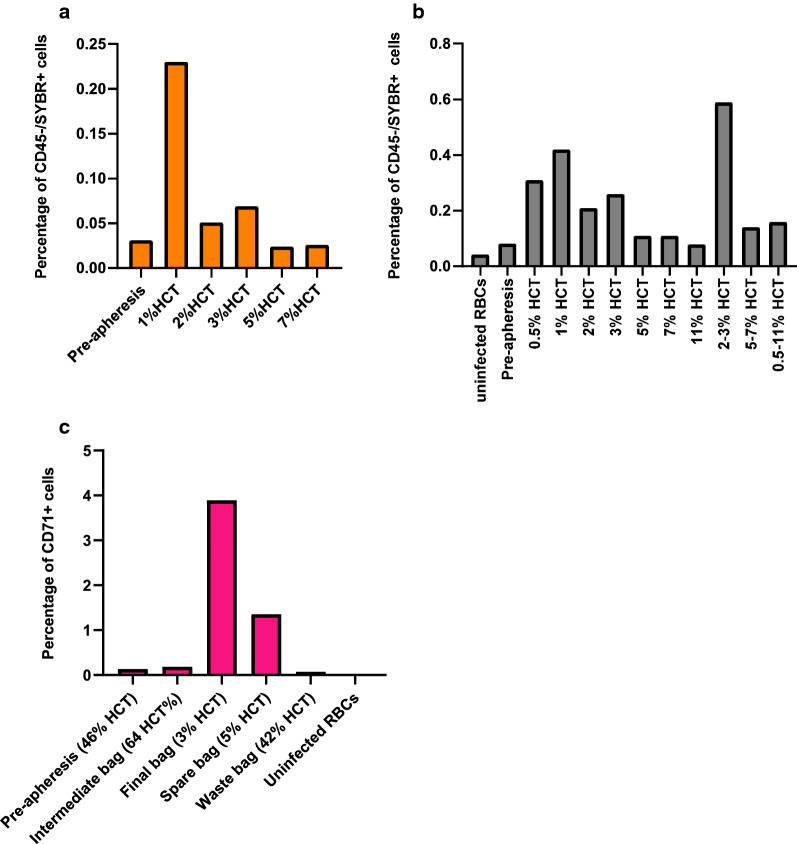
Flow cytometry cohorts 2 to 4. Percentage of CD45-/SYBR + in samples obtained from subject 2 (**a**) and subject 3 (**b**). The percentage of (CD71 +) reticulocytes from subject 4 (**c**)

In subjects 2 and 3, it was observed that as sampling was undertaken from progressively lower haematocrits there was an increase in the percentage of WBCs (Additional file 6: Figs. S10 and S11). A small percentage of SYBR Green I + and CD45- cells, which could represent either parasitized RBCs or reticulocytes, were detected in samples from subjects 2 and 3 (Fig. 4a, b); pooled samples collected at 2–3% HCT from subject 3 had the highest concentration (0.59%) of SYBR Green I + and CD45-cells (Fig. 4b). In subject 4, where CD71 antibody staining was used to identify reticulocytes, the highest levels of reticulocytes (CD71 + cells) were observed in the final bag (3% HCT) and the spare bag (5% HCT) (Fig. 4c). Microscopic analysis of these samples suggested that the reticulocyte population detected by flow cytometry consisted of uninfected reticulocytes. No parasitized RBCs or reticulocytes could be detected by flow cytometry, and thus indicating that these cells are absent or below the limit of detection by flow cytometry.

### Microscopy

The high concentration of WBCs in apheresis samples, meant many of the thick and thin blood smears were extremely difficult to read (Additional file 6: Tables S9 and S10). Among samples where it was possible to read the films, parasite counts were low, and in alignment with the counts obtained by qPCR (Additional file 6: Tables S9 and S10). Notwithstanding the technical difficulties in reading the slides and the low parasite counts, no apparent concentration of parasites was observed in any of the apheresis samples compared to pre-apheresis. The 3% HCT sample in subject 3 contained 7 parasitized cells compared to 0 in the pre-apheresis sample and the 1% HCT sample in subject 1 demonstrated a fourfold increase in the number of visualized parasites compared to pre-apheresis. In general, higher parasite numbers were seen in the lower HCT layers. The vast majority of parasitized RBCs contained ring form parasites, with trophozoites and gametocytes observed in samples from subjects 3 and 4 only (Additional file 6: Tables S9 and S10).

### Mosquito transmission

Membrane feeding of pre-apheresis or post Percoll enrichment samples were undertaken in all 4 subjects, but for logistical reasons membrane feeding on apheresis samples could only be undertaken in subjects 1 and 4 (Table 3).

None of the samples from subject 1 or 2 resulted in successful mosquito transmission (Table 3). In subject 3, the infection rate from the Percoll-enriched sample was 5.8-fold higher than the infection rate from the non-enriched pre-apheresis sample (99% vs 17.2% [Table 3]). Likewise, in subject 4, the infection rate from the final bag (3% HCT) was 5.5-fold higher than the infection rate from either the pre-apheresis sample or the intermediate bag (64%HCT) (pre-apheresis: 3.6%, intermediate bag: 4%, final bag: 20% [Table 3]).

The final sample bag (3% HCT) in subject 4 was the only sample obtained using apheresis that demonstrated an increase in transmission over samples obtained pre-apheresis. Due to logistic issues the spare bag (5% HCT), which contained the greatest enrichment for gametocytes compared to pre-apheresis when adjusted for red cell counts in subject 4 (Additional file 6: Fig. S4b) was not subject to membrane feeding.

### Additional 18S qPCR testing

To investigate for possible accumulation of parasites in the magnets and tubing structures in the apheresis cassette, qPCR testing was carried out on three blood clots with diameters of up to 1 cm found near two magnets, and a silo like structure that formed part of the single use apheresis tubing and processing cassette from subject 3 (Additional file 6: Fig. S13). Clots were thoroughly homogenized and tested by 18S qPCR. The greatest enrichment observed relative to pre-apheresis whole blood was 1.4-fold in the apheresis cassette magnet 2 sample (Additional file 6: Fig. S14 and Table S11).

To investigate whether haemolysis may be taking place in the apheresed blood resulting in release of parasite DNA into the extracellular fluid, plasma from apheresis samples in subject 3 was collected by centrifugation of the sample, with the plasma subject to 18S qPCR testing. However, no significant accumulation of parasite DNA was detected in the plasma compared to pre-apheresis (Additional file 6: Fig. S14 and Table S11).

## Discussion

Using apheresis it was possible to achieve modest concentration of both asexual and gametocyte stages of *P. vivax*. However, the modest level of parasite enrichment (4.9-fold and 1.45-fold for asexual parasites and gametocytes respectively) was deemed to be insufficient for downstream research. Furthermore the relatively low parasite levels, particularly of gametocytes, meant that the figures may be subject to chance variations in parasite levels.

Results of this study suggest that apheresis in healthy subjects infected with blood-stage *P. vivax* parasites is safe. No serious adverse events were encountered, with all adverse events having resolved by the end of study. The majority of adverse events were malaria related, and in line with previous *P. vivax* IBSM studies [7, 8]. Adverse events related to apheresis consisted largely of asymptomatic transient reductions in haematology parameters.

After correction for red blood cell number, parasite quantitation by qPCR suggested that both asexual parasites and gametocytes were preferentially concentrated in the lower HCT layers (Additional file 6: Figs. S2 and S4). Furthermore, the pre-clinical experiment demonstrated a selective concentration of *P. falciparum* gametocytes compared to asexual parasites in lower HCT layers (Additional file 6: Fig. S6A and Table S4). However as the pre-clinical experiment did not involve collection of blood from an infected subject they do not fully replicate those collected ex vivo, for example being subject to host interactions such as sequestration, thus limiting the utility of this work in predicting what would be the situation in natural infection. Findings from the pre-clinical and clinical experiments were consistent with the previously published observations of the buoyancy of *Plasmodium* parasites [24].

Several scenarios were considered to explain why concentration of parasites using apheresis was lower than expected. Firstly, as gametocyte concentrations in apheresis samples were around the level of detection of the *pvs25* by qRT-PCR, minor variation in concentration may have been difficult to quantify [25]. Secondly, infected RBCs containing magnetic haemozoin [26] may have attached to ferromagnetic components of the apheresis apparatus. Thirdly, it is possible that lysis of asexual parasites and gametocytes occurred during the apheresis procedure. This latter hypothesis is supported by a recent report suggesting that parasite maturation results in increasing fragility of *P. vivax* infected red blood cells [27]. Although it may have been possible to assess for low level haemolysis during the procedure, for example by measuring haptoglobin levels, controlling for a range of other variables would have been difficult.

An enhanced level of transmission to mosquitoes compared to whole blood samples collected pre-apheresis was only observed on one occasion (final sample bag [3% HCT] in subject 4), corresponding to the higher gametocyte concentration in this sample compared to the pre-apheresis whole blood (Fig. 4b). A possible explanation of the low success in the transmission studies was the difficulty in maintaining tight temperature control to prevent exflagellation of male gametocytes [28], thereby negatively impacting gametocyte infectivity [29]. Blood was most vulnerable to a temperature drop whilst in the apheresis equipment itself. It was not possible to heat apheresis equipment, and it was deemed impractical to heat the room where apheresis took place to > 35 °C. Temperature monitoring was not possible during the experiments. However, the demonstration of transmission success in the final bag (3% HCT) indicates that at least some gametocytes were maintained within a temperature range that did not trigger exflagellation. Regardless of the underlying cause, recent reports of success in improving concentration of gametocytes and enhanced transmission by either Percoll [9, 10] or magnetic bead [30] enrichment suggests that such methods are superior for concentration of gametocytes for mosquito transmission experiments.

Each of the vials from the HMP bank used in this study to infect subjects contain 2.08 × 10^6^ parasites. Based on the greatest level of asexual enrichment per ml of sample observed (7% HCT, subject 1), calculations suggest that apheresis alone can create parasites vials with a maximum of 7.92 × 10^4^ parasites (Additional file 6). Therefore, to create a HMP bank using an apheresis approach equivalent to the one used to infect volunteers in this study a > 25-fold increase in pre-apheresis parasitaemia would be required, equating to > 650,000 parasites/ml. Attaining such high parasitaemia would likely lead to significant discomfort in study volunteers, and therefore raise significant ethical concerns. Therefore, unless significant improvements in enrichment can be attained, apheresis should not be used to create HMP banks, and the current practice of collecting blood by venesection is preferable.

Strengths of the study include the wide sampling across HCT layers (1%, 2%, 3%, 5% and 7%) and the use of multiple methods to enumerate parasites (flow cytometry, microscopy and qPCR). Although only a small number of subjects were studied using this approach, the lack of promising data meant that continuation of the trial was deemed inappropriate by the safety review team and the study was terminated.

## Conclusion

Given the moderate levels of enrichment and the significant ethical, financial and logistical concerns surrounding *P. vivax* IBSM studies, it was decided that further apheresis studies are not warranted at this point. If apheresis were to be used again in the context of Malaria VIS, it should ideally only be carried out in subjects with normal haematological parameters at screening to avoid potential complications or the need for blood transfusion.

## Supplementary Information

**Additional file 1. **Apheresis of subjects with induced blood stage* p. vivax* protocol.

**Additional file 2. **Apheresis Cohort 1 standard operating procedure.

**Additional file 3. **Apheresis Cohort 2 standard operating procedure.

**Additional file 4. **Apheresis Cohort 3 standard operating procedure.

**Additional file 5. **Apheresis Cohort 4 standard operating procedure.

**Additional file 6. **Supplementary Material v1.0.

**Additional file 7. **Apheresis Cohort 1 laboratory standard operating procedure.

**Additional file 8. **Apheresis Cohort 2 laboratory standard operating procedure.

**Additional file 9. **Apheresis Cohort 3 laboratory standard operating procedure.

**Additional file 10. **Apheresis Cohort 4 laboratory standard operating procedure.

**Additional file 11. **Spectra Optia Brochur System Overview.

## Data Availability

Data collected for this study will be made available immediately after article publication with no end date. De-identified datasets containing the variables analysed for the primary and secondary objectives will be made available as well as other supporting documents (e.g., protocol and informed consent). Investigators who seek access to individual subject data will contact the corresponding author (AO) to receive instructions on the formal request process, which will include the submission of a brief proposal. The proposal will be reviewed for merit and feasibility by the corresponding author (AO). Investigators will be notified of the decision within 30 days of receipt. If the request is accepted, a data transfer agreement covering relevant conditions will be required.

## Abbreviations

VIS: Volunteer infection studies
CHMI: Controlled human malaria infection
IBSM: Induced blood stage malaria
RCE: Red cell exchange
QIMR: Queensland Institute of Medical Research
RBWH: Royal Brisbane and Women’s Hospital
HREC: Human Research Ethics Committee
GCP: Good Clinical Practice
HCT: Haematocrit
CTCAE: Common terminology of clinical trial adverse events
CMNC: Continuous mononuclear cell collection
PCR: Polymerase chain reaction
qPCR: Quantitative polymerase chain reaction
qRT PCR: Quantitative reverse transcriptase polymerase chain reaction
WBCs: White blood cells
RBCs: Red blood cells
MFA: Membrane feeding assays
DMFA: Direct membrane feeding assays
PMN: Polymorphonuclear
LLN: Lower limit of normal
ULN: Upper limit of normal
SAE: Serious adverse event
AE: Adverse event
