# Supplementary material for: Safety and feasibility of apheresis to harvest and concentrate parasites from subjects with induced blood stage Plasmodium vivax infection
Source: Malar J. 2021 Jan 14;20:43. doi: 10.1186/s12936-021-03581-w (PMC7807416; doi:10.1186/s12936-021-03581-w)
Supplement: Supplementary file 1 — Additional file 1. Apheresis of subjects with induced blood stage p. vivax protocol. [file 12936_2021_3581_MOESM1_ESM.docx]

**
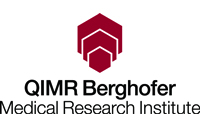
**

**Apheresis of subjects with induced blood stage *Plasmodium vivax***

**Protocol Identifying Number:** QP17C14/P2355

**Principal Investigator:** Prof. James McCarthy

**Sponsor:** QIMR Berghofer Medical Research Institute (QIMR Berghofer),

300 Herston Road, Herston QLD 4006

**Version 4.0**

**22 Oct 2018**

CONFIDENTIALITY STATEMENT

This document contains information that is privileged or confidential. As such, it may not be disclosed unless specific prior permission is granted in writing by QIMR Berghofer or such disclosure is required by federal or other laws or regulations. Persons to whom any of this information is to be disclosed must first be informed that the information is confidential. These restrictions on disclosure will apply equally to all future information supplied, which is indicated as privileged or confidential.

Table of Contents

[LIST OF ABBREVIATIONS 1](#_Toc517181350)

[STATEMENT OF COMPLIANCE 3](#_Toc517181351)

[PROTOCOL SUMMARY 5](#_Toc517181352)

[SCHEMATIC OF STUDY DESIGN 10](#_Toc517181353)

[1 KEY ROLES 11](#_Toc517181354)

[2 INTRODUCTION: BACKGROUND INFORMATION AND SCIENTIFIC RATIONALE 13](#_Toc517181355)

[2.1 Background Information 13](#_Toc517181356)

[2.2 Rationale 14](#_Toc517181357)

[2.3 Potential Risks and Benefits 15](#_Toc517181358)

[2.3.1 Known Potential Risks 15](#_Toc517181359)

[2.3.2 Known Potential Benefits 21](#_Toc517181360)

[3 OBJECTIVES AND PURPOSE 21](#_Toc517181361)

[4 STUDY DESIGN AND ENDPOINTS 22](#_Toc517181362)

[4.1 Description of the Study Design 22](#_Toc517181363)

[4.2 Study Endpoints 23](#_Toc517181364)

[4.2.1 Primary Endpoint 23](#_Toc517181365)

[4.2.2 Secondary Endpoints 23](#_Toc517181366)

[4.2.3 Exploratory Endpoints 23](#_Toc517181367)

[4.2.4 Exploratory Endpoints 23](#_Toc517181368)

[5 STUDY ENROLLMENT AND WITHDRAWAL 24](#_Toc517181369)

[5.1 Subject Inclusion Criteria 24](#_Toc517181370)

[5.2 Subject Exclusion Criteria 25](#_Toc517181371)

[5.3 Strategies for Recruitment and Retention 30](#_Toc517181372)

[5.4 Subject Withdrawal or termination 30](#_Toc517181373)

[5.4.1 Reasons for Withdrawal or Termination 30](#_Toc517181374)

[5.4.2 Handling of Subject Withdrawals or termination 31](#_Toc517181375)

[5.5 Premature Termination or Suspension of Study 31](#_Toc517181376)

[6 STUDY AGENT 32](#_Toc517181377)

[6.1 Study Agent(s) and Control Description 32](#_Toc517181378)

[6.1.1 Acquisition 32](#_Toc517181379)

[6.1.2 Formulation, Appearance, Packaging, and Labeling 32](#_Toc517181380)

[6.1.3 Product Storage and Stability 33](#_Toc517181381)

[6.1.4 Preparation 33](#_Toc517181382)

[6.1.5 Dosing and Administration 33](#_Toc517181383)

[6.1.6 Route of Administration 35](#_Toc517181384)

[6.1.7 Starting Dose and Dose Escalation Schedule 35](#_Toc517181385)

[6.1.8 Dose Adjustments/Modifications/Delays 35](#_Toc517181386)

[6.1.9 Duration of Therapy 35](#_Toc517181387)

[6.1.10 Tracking of Dose 35](#_Toc517181388)

[6.2 Study agent Accountability Procedures 35](#_Toc517181389)

[7 STUDY PROCEDURES AND SCHEDULE 36](#_Toc517181390)

[7.1 Study Procedures/Evaluations 36](#_Toc517181391)

[7.1.1 Study specific procedures 36](#_Toc517181392)

[7.1.2 Standard of care study procedures 38](#_Toc517181393)

[7.2 Laboratory Procedures/Evaluations 38](#_Toc517181394)

[7.2.1 Clinical Laboratory Evaluations 38](#_Toc517181395)

[7.2.2 Other Assays or Procedures 41](#_Toc517181396)

[7.2.3 Specimen Preparation, Handling, and Storage 43](#_Toc517181397)

[7.2.4 Specimen Shipment 43](#_Toc517181398)

[7.3 Study Schedule 43](#_Toc517181399)

[7.3.1 Screening 43](#_Toc517181400)

[7.3.2 Enrollment/Baseline 45](#_Toc517181401)

[7.3.3 Follow-up 46](#_Toc517181402)

[7.3.4 Final Study Visit 50](#_Toc517181403)

[7.3.5 Early Termination Visit 51](#_Toc517181404)

[7.3.6 Unscheduled Visit 51](#_Toc517181405)

[7.3.7 Schedule of Events Table 51](#_Toc517181406)

[7.4 Justification for Sensitive Procedures 55](#_Toc517181407)

[7.5 Concomitant Medications, Treatments, and Procedures 55](#_Toc517181408)

[7.5.1 Precautionary Medications, Treatments, and Procedures 55](#_Toc517181409)

[7.6 Prohibited Medications, Treatments, and Procedures 55](#_Toc517181410)

[7.7 Prophylactic Medications, Treatments, and Procedures 56](#_Toc517181411)

[7.8 Rescue Medications, Treatments, and Procedures 56](#_Toc517181412)

[7.9 Subject Access to Study Agent At Study Closure 56](#_Toc517181413)

[8 ASSESSMENT OF SAFETY 56](#_Toc517181414)

[8.1 Specification of Safety Parameters 56](#_Toc517181415)

[8.1.1 Definition of Adverse Events (AE) 56](#_Toc517181416)

[8.1.2 Definition of Serious Adverse Events (SAE) 57](#_Toc517181417)

[8.1.3 Definition of Unanticipated Problems (UP) 58](#_Toc517181418)

[8.2 Classification of an Adverse Event 58](#_Toc517181419)

[8.2.1 Severity of Event 58](#_Toc517181420)

[8.2.2 Relationship to Study Agent 59](#_Toc517181421)

[8.2.3 Expectedness 60](#_Toc517181422)

[8.3 Time Period and Frequency for Event Assessment and Follow-Up 60](#_Toc517181423)

[8.4 Reporting Procedures 61](#_Toc517181424)

[8.4.1 Adverse Event Reporting 61](#_Toc517181425)

[8.4.2 Serious Adverse Event Reporting 62](#_Toc517181426)

[8.4.3 Unanticipated Problem Reporting 62](#_Toc517181427)

[8.4.4 Events of Special Interest 63](#_Toc517181428)

[8.4.5 Reporting of Pregnancy 63](#_Toc517181429)

[8.5 Study Halting Rules 63](#_Toc517181430)

[8.6 Safety Oversight 63](#_Toc517181431)

[9 CLINICAL MONITORING 63](#_Toc517181432)

[10 STATISTICAL CONSIDERATIONS 64](#_Toc517181433)

[10.1 Statistical and Analytical Plans 64](#_Toc517181434)

[10.2 Statistical Hypotheses 64](#_Toc517181435)

[10.3 Analysis Datasets 64](#_Toc517181436)

[10.4 Description of Statistical Methods 64](#_Toc517181437)

[10.4.1 General Approach 64](#_Toc517181438)

[10.4.2 Analysis of the Primary Efficacy Endpoint(s) 65](#_Toc517181439)

[10.4.3 Analysis of the Secondary Endpoint(s) 65](#_Toc517181440)

[10.4.4 Safety Analyses 65](#_Toc517181441)

[10.4.5 Adherence and Retention Analyses 65](#_Toc517181442)

[10.4.6 Baseline Descriptive Statistics 65](#_Toc517181443)

[10.4.7 Planned Interim Analyses 65](#_Toc517181444)

[10.4.8 Additional Sub-Group Analyses 66](#_Toc517181445)

[10.4.9 Multiple Comparison/Multiplicity 66](#_Toc517181446)

[10.4.10 Tabulation of Individual Response Data 66](#_Toc517181447)

[10.4.11 Exploratory Analyses 66](#_Toc517181448)

[10.5 Sample Size 66](#_Toc517181449)

[10.6 Measures to Minimize Bias 66](#_Toc517181450)

[10.6.1 Enrollment/ Randomization/ Masking Procedures 66](#_Toc517181451)

[10.6.2 Evaluation of Success of Blinding 67](#_Toc517181452)

[10.6.3 Breaking the Study Blind/Subject Code 67](#_Toc517181453)

[11 SOURCE DOCUMENTS AND ACCESS TO SOURCE DATA/DOCUMENTS 67](#_Toc517181454)

[12 QUALITY ASSURANCE AND QUALITY CONTROL 67](#_Toc517181455)

[13 ETHICS/PROTECTION OF HUMAN SUBJECTS 68](#_Toc517181456)

[13.1 Ethical Standard 68](#_Toc517181457)

[13.2 Ethical Review 68](#_Toc517181458)

[13.3 Informed Consent Process 70](#_Toc517181459)

[13.3.1 Consent/assent and Other Informational Documents Provided to Subjects 70](#_Toc517181460)

[13.3.2 Consent Procedures and Documentation 70](#_Toc517181461)

[13.4 Subject and data Confidentiality 70](#_Toc517181462)

[13.4.1 Research Use of Stored Human Samples,Specimens or Data 71](#_Toc517181463)

[13.5 Future Use of Stored Specimens 71](#_Toc517181464)

[14 DATA HANDLING AND RECORD KEEPING 72](#_Toc517181465)

[14.1 Data Collection and Management Responsibilities 72](#_Toc517181466)

[14.2 Study Records Retention 72](#_Toc517181467)

[14.3 Protocol Deviations 72](#_Toc517181468)

[14.4 Publication and Data Sharing Policy 73](#_Toc517181469)

[15 STUDY ADMINISTRATION 74](#_Toc517181470)

[15.1 Study Leadership 74](#_Toc517181471)

[15.2 Liability/Indemnity/Insurance 74](#_Toc517181472)

[16 CONFLICT OF INTEREST POLICY 74](#_Toc517181473)

[17 LITERATURE REFERENCES 74](#_Toc517181474)

[APPENDIX 1](#_Toc517181475)

#

# LIST OF ABBREVIATIONS

| ACTH | Adrenocorticotrophic Hormone |
| --- | --- |
| AE | Adverse Event |
| AESI | Adverse Event of Special Interest |
| ALT | Alanine aminotransferase |
| AST | Aspartate aminotransferase |
| BSPC | Blood Stage Plasmodium Challenge |
| CHMI | Controlled Human Malaria Infection |
| CMNC | Continuios Mononuclear Cell |
| CMV | Cytomegalovirus |
| CRF | Case Report Form |
| CRU | Clinical Research Unit |
| CTCAE | Common Terminology Criteria for Adverse Events |
| DFA | Direct feeding assay |
| DRE | Disease-Related Events |
| EBV | Epstien-Barr Virus |
| ECG | Electrocardiogram |
| eCRF | Electronic Case Report Form |
| EOS | End of Study |
| FSH | Follicle Stimulating Hormone |
| G6PD | Glucose-6 phosphate dehydrogenase |
| GCP | Good Clinical Practice |
| GMP | Good Manufacturing Practices |
| HMP | Human Malaria Parasite |
| HREC | Human Research Ethics Committee |
| IB | Investigator’s Brochure |
| IBSM | Induced Blood Stage Malaria |
| ICH | International Conference on Harmonisation |
| IMM | Local Independent Medical Monitor |
| IUD | Intrauterine |
| LFT | Liver function test |
| MCB | Master Cell Bank |
| MedDRA | Medical Dictionary for Regulatory Activities |
| MFA | Membrane feeding assay |
| PBMC | Perphieral Blood Mononuclear Cells |
| PI | Principal Investigator |
| PRN | Pro Re Nata (as needed) |
| PT | Preferred Term |
| QIMR Berghofer | Queensland Institute of Medical Research Berghofer |
| qPCR | Quantitative polymerase chain reaction |
| qRT-PCR | Quantitative reverse transcription polymerase chain reaction |
| Rh | Rhesus |
| SAE | Serious Adverse Event |
| SAP | Statistical Analysis Plan |
| SCID | Severe Combined Immunodeficiency |
| SD | Standard Deviation |
| SOC | System Organ Class |
| SOP | Standard Operating Procedure |
| SRT | Safety Review Team |
| SUSAR | Suspected Unexpected Serious Adverse event |
| TEAE | Treatment-Emergent Adverse Event |
| WHO | World Health Organization |
| WOCBP | Women of Childbearing Potential |

# STATEMENT OF COMPLIANCE

**Investigator declaration**

I have read the protocol and agree that it contains all necessary details for carrying out the study as described. I will conduct this protocol as outlined herein and will make a reasonable effort to complete the study within the time designated.

I agree to personally conduct or supervise the described study.

The study will be conducted in accordance with the following:

- World Medical Association Declaration of Helsinki – Ethical Principles for Medical Research Involving Human Subjects (Fortaleza, Brazil 2013).
- NHMRC National Statement on Ethical Conduct in Human Research (2007, updated May 2015).
- Notes for Guidance on Good Clinical Practice – Annotated with TGA Comments (CPMP/ICH/135/95), as adopted by the Australian Therapeutic Goods Administration (July 2000).
- Current ethics approved Clinical Trial Protocol.

I agree to inform all subjects that the study drug is being used for investigational purposes and I will ensure that the requirements related to obtaining informed consent are in accordance with ICH Guidelines for Good Clinical Practice (GCP) section 4.8 and local requirements.

I agree to report adverse events that occur in the course of the study to the Sponsor in accordance with ICH Guidelines for GCP section 4.11 and local requirements.

I have read and understand the information in the Investigator’s Brochure, including the potential risks and side effects of the study drug.

I agree to promptly report to the Human Research Ethics Committee (HREC) all changes in the research activity and all unanticipated problems involving risk to subjects. I will not make any changes to the conduct of the study without HREC and Sponsor approval, except when necessary to eliminate apparent immediate harm to subjects.

I agree to maintain adequate and accurate records and make those records available in accordance with ICH Guidelines for GCP section 4.11 and local requirements.

I agree to ensure that all associates, colleagues, and employees assisting in the conduct of the study are informed about their obligations in meeting the above commitments.

I understand that the study may be terminated or enrolment suspended at any time by the Sponsor, with or without cause, or by me if it becomes necessary to protect the best interest of the subjects.

______________________________ Date: _____________________________

Prof. James McCarthy, Principal Investigator

**Signatories**

The undersigned agrees that the protocol was written in accordance with the World Medical Association Declaration of Helsinki – Ethical Principles for Medical Research Involving Human Subjects (Fortaleza, Brazil 2013), the National Statement on Ethical Conduct in Human Research (NHMRC, 2007, updated May 2015) and the Notes for Guidance on Good Clinical Practice – Annotated with TGA Comments (CPMP/ICH/135/95), as adopted by the Australian Therapeutic Goods Administration (July 2000).

| **Name** | Signature | Date |
| --- | --- | --- |
| Protocol Writer:  **Dr Anand Odedra, MBBS**  Q-Pharm Pty Ltd (Visiting Medical Officer) and QIMR Berghofer Medical Research Institute |  |  |

**This clinical trial protocol has been reviewed and approved by the Sponsor.**

| **Name** | Signature | Date |
| --- | --- | --- |
| Sponsor Representative:  **Prof. David Whiteman, MBBS, PhD, FAFPHM**  Deputy Director  QIMR Berghofer Medical Research Institute |  |  |

# PROTOCOL SUMMARY

| **Title:** | Apheresis of subjects with induced blood stage *Plasmodium vivax* |
| --- | --- |
| **Précis:** | This is a Phase 1 exploratory study designed to determine the safety and feasibility of using apheresis as a method for extracting all lifecycle stages of malaria parasites from the blood of healthy subjects experimentally infected with blood stage *P. vivax*. This study will be conducted in up to 8 subjects (8 cohorts of 1 subject each).  Subjects will be inoculated intravenously on Day 0 with approximately 1100 viable *P. vivax* HMPBS02-*Pv* parasite-infected erythrocytes. On an outpatient basis, subjects will be monitored daily via phone and then will attend the clinical unit daily from 4 days post-inoculation for blood sampling to measure parasitaemia via qPCR targeting the *P. vivax* 18S rRNA gene (referred to as malaria 18S qPCR), to monitor symptoms and signs of malaria, and to record AEs.  The threshold for the commencement of apheresis and subsequent antimalarial rescue treatment with artemether/lumefantrine will occur when parasitaemia is >20,000 parasites/mL or the Malaria Clinical Score is >6 (within 24 hours of notification) or at the Investigator’s discretion. On the day that this threshold is reached (expected to occur on Day 10, 11 or 12), the subject will be admitted to the clinical unit for initial safety assessments before undergoing apheresis whilst being supervised by the apheresis specialist nurse.  The subject will then be administered the first dose of artemether/lumefantrine and will remain confined within the clinical unit for 72 hours (or less -48h minimum- at the discretion of the investigator if the subject is deemed clinically well and the subject is happy for early release) to monitor for safety and tolerability of apheresis and rescue therapy, and to ensure adequate clinical and parasitological response to treatment. In the unlikely event that artemether/lumefantrine fails to clear parasitaemia, subjects will be treated with chloroquine. If oral administration of either artemether/lumefantrine or chloroquine is not possible (eg. the subject is vomiting), the subject will receive intravenous treatment with artesunate. After discharge from the clinical unit, subjects will be followed up on an out-patient basis for monitoring of safety and parasite clearance. Follow-up for safety assessments will be performed on Day 28±3, Day 56±7 (phone call only), and Day 90±7 (End of Study).  Subjects will also be evaluated for the presence of sexual parasite stages (gametocytes) and other parasite lifecycle stages in the blood during the study using reverse transcriptase qPCR (qRT-PCR). Parasite lifecycle stage qRT-PCR may also be used at the Investigator’s discretion to determine the timing of direct feeding assays (DFAs) and/or membrane feeding assays (MFAs) which may occur at up to 2 time-points prior to apheresis and rescue treatment initiation. |
| **Objectives:** | Primary objectives   - To further assess the safety of the *P. vivax* induced blood stage malaria (IBSM) model following inoculation of healthy subjects with *P. vivax* isolate HMPBS02-*Pv*. - To assess the safety of apheresis in the *P. vivax* IBSM model following inoculation of healthy subjects with *P. vivax* isolate HMPBS02-*Pv*.   Secondary objectives   - To assess the feasibility of apheresis as a method of extracting and concentrating all stages of malaria parasites following inoculation of healthy subjects with *P. vivax* isolate HMPBS02-*Pv*. - To evaluate the success of cryopreservation of all stages of *P. vivax* parasites from blood products extracted via apheresis from healthy subjects inoculated with. *P. vivax* isolate HMPBS02-*Pv*.   Exploratory   - To explore the potential for apheresis to be used as a method for producing a *P. vivax* Human Malaria Parasite (HMP) bank to be used for future IBSM studies. - To evaluate the transmission of *P. vivax* gametocytes to mosquitoes.   Exploratory optional   - To collect and store plasma and peripheral blood mononuclear cells (PBMCs) harvested using apheresis for future research on the immunology and pathophysiology of *P. vivax*. - To assess development and/or functionality of antibodies to asexual stage parasites and/or gametocytes. |
| **Endpoints:** | Primary endpoint  The primary objectives will be assessed by adverse events (AE) recording, safety laboratory parameters, vital signs recording, electrocardiograms (ECGs), physical examination findings and Malaria Clinical Score recording.  Secondary endpoints  The secondary objectives will be assessed as follows:   - The feasibility of apheresis as a method of extracting and concentrating all stages of malaria parasites will be determined by qPCR, qRT-PCR, microscopy, and flow cytometry on blood products extracted using apheresis, and expressed as a percentage of baseline parasitaemia (pre-apheresis). - The success of cryopreservation of gametocytes will be determined by measuring their infectivity to mosquitoes using a membrane feeding assay, reported as prevalence of infection (percentage of oocyst positive mosquitoes). The success of cryopreservation of asexual parasites will be determined based on their viability tested using *in vitro* re-invasion assay.   Exploratory endpoints  The exploratory objectives will be assessed as follows:   - The potential for apheresis to be used as a method for producing a *P. vivax* HMP bank will be determined based on the efficiency of extraction and concentration of parasites, the viability of extracted parasites, the successful leukodepletion of the sample, and the confirmation that the donor subject is free from blood-borne infections. - Transmission of *P. vivax* gametocytes to mosquitoes will be determined using a direct feeding assay and/or membrane feeding assay, and reported as prevalence of infection (percentage of oocyst positive mosquitoes).   Optional exploratory endpoints  The optional exploratory objectives will be assessed as follows:   - The successful collection of PBMCs will be determined, if considered appropriate, by cell counts and cell viability tested using trypan blue staining. - Detection and evaluation of anti-blood-stage or anti-gametocyte specific antibodies using immunological assays (including but not limited to enzyme-linked immunosorbent assays and indirect immunofluorescence assays). |
| **Population:** | A maximum of 8 subjects will be enrolled in this study (8 cohorts of 1 subject each). Subjects will be malaria naïve healthy adults (male), aged between 18-55 years old, who meet all of the inclusion criteria and none of the exclusion criteria. |
| **Phase:** | Phase 1 (exploratory study) |
| **Number of Sites enrolling subjects:** | Single Centre  Q-Pharm Pty Ltd  Level 5, 300C Herston Rd and  Level 6, Block 8, Royal Brisbane and Women’s Hospital  Herston, QLD 4006, Australia |
| **Description of Study**  **Agent(s):** | **Malaria challenge agent**:  The *P.vivax* HMPBS02-*Pv* Master Cell Bank (MCB) was produced from blood donated from a returned traveller from India who presented with clinical manifestations of malaria. Each inoculum dose will be prepared aseptically from an aliquot of the *P. vivax* HMPBS02-*Pv* MCB. Each subject will be inoculated intravenously with a dose of approximately 1100 viable parasite-infected erythrocytes in 2 mL saline for injection.  **Antimalarial rescue medication:**  Artemether/lumefantrine  Riamet^®^ (20 mg Artemether and 120 mg Lumefantrine) will be administered to all subjects post apheresis or earlier if apheresis cannot performed for safety reasons. A course of treatment comprises 6 doses of 4 tablets administered orally over a period of 60 hours (total course of 24 tablets). Each dose of tablets should be taken with food or drinks rich in fat (e.g., milk).  Chloroquine (if required)  Subjects will be administered Chloroquine if artemether/lumefantrine fails to clear the malaria parasites. Chloroquine tablets each containing 250 mg chloroquine phosphate (equivalent to 155 mg chloroquine base) will be administered orally. An initial dose of chloroquine will be administered as 4 tablets, followed by a dose of 2 tablets each at 6, 24 and 48 hours (i.e. a total dose of 2.5 g chloroquine phosphate [1.550 g base]).  Artesunate (if required)  Treatment of subjects with intravenous artesunate will only occur in the event that subjects are unable to complete oral treatment with either artemether/lumefantrine or chloroquine (e.g. the subject is vomiting). This would be done at the recommended dose regime of 2.4 mg/kg at approximately 0, 12, 24 hours and then daily for up to 7 days or until able to take oral drugs. |
| **Study Duration:** | Approximately 12 months. |
| **Subject Duration:** | Approximately 4 months. |

# SCHEMATIC OF STUDY DESIGN


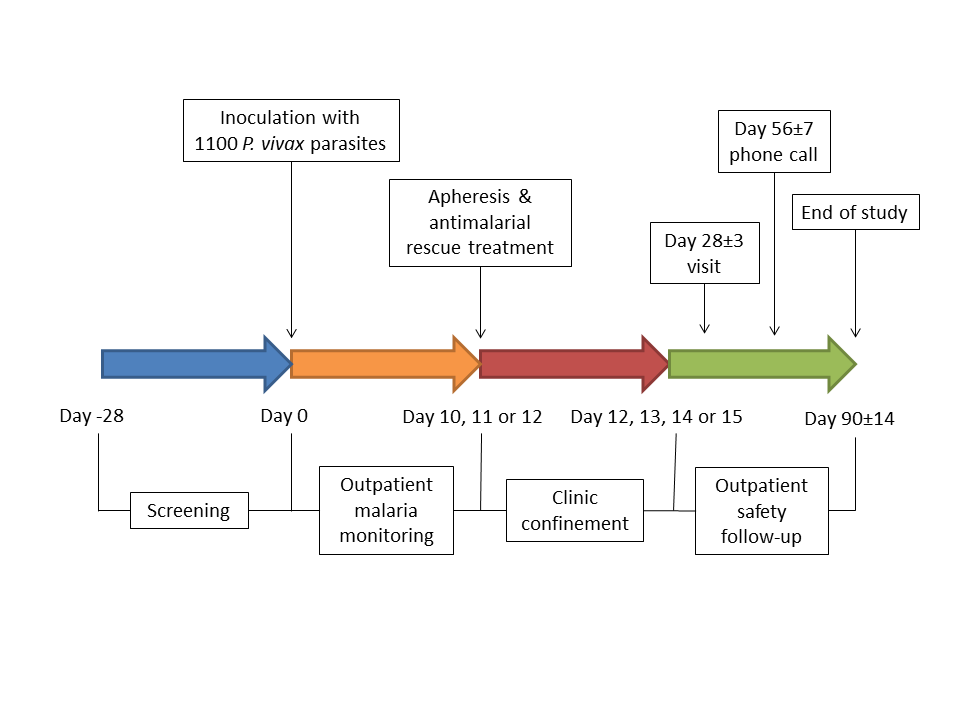


# 1 KEY ROLES

| Principal Investigator | Prof. James McCarthy, MBBS  Q-Pharm Pty Ltd (visiting Medical Officer) and  QIMR Berghofer Medical Research Institute  Level 5, 300C Herston Rd  Herston, QLD 4006, Australia  Tel: +61(0)7 3845 3647 or +61 (0)7 3845 3636  Email: [j.mccarthy@uq.edu.au](mailto:j.mccarthy@uq.edu.au) |
| --- | --- |
| Co-Principal Investigator | Dr Anand Odedra, MBBS  Q-Pharm Pty Ltd (visiting Medical Officer) and  QIMR Berghofer Medical Research Institute  Level 5, 300C Herston Rd  Herston, QLD 4006, Australia  Tel: +61(0)7 3845 3647 or +61 (0)411041391  Email: anand.odedra@qimrberghofer.edu.au |
| Co-Principal Investigator | Bridget Barber, MD  Q-Pharm Pty Limited (Visiting Medical Officer) and  QIMR Berghofer Medical Research Institute  Level 5, 300C Herston Rd  Herston, QLD 4006, AUSTRALIA  Tel: +61 (0)4 24 73 71 53  Email: Bridget.Barber@qimrberghofer.edu.au |
| Co-Principal Investigator | Professor David Lalloo, MBBS MD FRCP MRCP  Liverpool School of Tropical Medicine  Pembroke Place Liverpool  L3 5QA UK  Tel: +44 (0)151 705 3218  Email: david.lalloo@lstmed.ac.uk |
| Collaborating Principal Investigator | A/Prof. Glen Kennedy, MBBS  Acting Executive Director  Cancer Care Services  Metro North HHS  Herston QLD 4029, Australia  Tel: +61(0)7 3646-7692  glen.kennedy@health.qld.gov.au |
| Co-Investigator | Dr Paul Griffin, MBBS, FRACP, FRCPA  Manager of Medical Services and Principal Investigator,  Q-Pharm Pty Ltd  Level 5, 300C Herston Rd  Herston, QLD 4006, Australia  Tel: +61 (0)7 3845 3636 or +61 (0)402077302  Email: [p.griffin@qpharm.com.au](mailto:p.griffin@qpharm.com.au) |
| Trial Sponsor | QIMR Berghofer Medical Research Institute  300 Herston Road  Herston QLD 4006, Australia  Tel: +61 (0)7 3362 0222 |
| Sponsor Signatory | Prof. David Whiteman, MBBS, PhD, FAFPHM  Deputy Director  QIMR Berghofer Medical Research Institute  300 Herston Rd,  Herston, QLD 4006, Australia  Tel: +61 (0)7 3362 0222  David.Whiteman@qimrberghofer.edu.au |
| Independent Medical Monitor | Professor Dennis Shanks  Australian Army Malaria Institute  Gallipoli Barracks  Enoggera Qld 4051  Ph +61 (0)7 3332 4931  Dennis.SHANKS@defence.gov.au |
| Institutional Ethics Committe | QIMR Berghofer Medical Research Institute Human Research Ethics Committee (QIMR Berghofer-HREC; EC00278)  Locked Bag 2000, Royal Brisbane and Women’s Hospital, Brisbane, QLD 4029, Australia  Tel: +61 (0)7 3362 0117 |
| Sponsor’s Monitors | Clinical Network Services (CNS) Pty Ltd  Level 4, 88 Jephson St  Toowong QLD 4066, Australia  Tel: +61 (0)7 3719 6000 |
| Clinical Study Centre | Q-Pharm Pty Ltd  Level 5, 300C Herston Rd and  Level 6, Block 8, Royal Brisbane and Women’s Hospital  Herston, QLD 4006, Australia  Tel: +61 (0)7 3845 3636 |
| Clinical Laboratories | Clinical laboratory measurements  Sullivan Nicolaides Pathology Central Laboratory (SNP)  24 Hurworth Street  Bowen Hills, QLD 4006, Australia  Tel: +61 (0)7 3377 8782  Parasite quantification in blood samples  Queensland Paediatric Infectious Diseases Laboratory (Q-PID), SASVRC, Level 8, Centre for Children’s Health Research  62 Graham Street,  South Brisbane, QLD 4101, Australia  Tel: +61 (0)7 3069 7464 |

# 2 INTRODUCTION: BACKGROUND INFORMATION AND SCIENTIFIC RATIONALE

## 2.1 Background Information

Despite the decrease in malaria incidence achieved in the last 17 years, this parasitic disease still threatens almost half of the world’s population. In 2015, there were 214 million cases of malaria and 438,000 deaths (1). Most malaria cases occurred in sub-Saharan Africa. However, Asia, Latin America, the Middle East and parts of Europe are also at risk (1).

Whilst *Plasmodium falciparum (P. falciparum*) is the most prevalent malaria parasite in Africa, *Plasmodium vivax* (*P. vivax*) has a wider geographical distribution. In 2015, 41% of the malaria cases that occurred outside the African continent were caused by *P. vivax* resulting in an estimated 3,100 deaths (1). Moreover, 70 to 80 million cases per year of relapsing malaria occur due to infection with *P. vivax*.

The World Health Organization (WHO) has declared that the response to malaria is a global development priority and has changed their recommendation from control to eradication programs. A robust development pipeline of potential drug candidates is required in order to meet this target and the screening process for determining safety and clinical efficacy of potential antimalarials requires fast, efficient test systems.

Controlled human malaria infection (CHMI) is increasingly being used to evaluate antimalarial drug candidates (2-4, 14-24). CHMI studies with *P. falciparum*  and *P. vivax* use the induced blood stage malaria (IBSM) model, whereby subjects are infected with blood stage malaria parasites. The availability of the IBSM model offers a pathway to test the efficacy of *P. falciparum*  and/or *P. vivax* vaccines and drugs in non-immune subjects, in a rapid and cost effective manner. One of the advantages of the blood stage challenge model is its ability to allow analysis of antimalarial efficacy, by providing opportunities to monitor parasite growth after challenge. This serves as a robust surrogate for the activity of a test compound/vaccine. Validation studies have shown a high correlation between natural and experimental infections, which further justifies the use of CHMI for testing new vaccines or drugs (2, 4). The safety and utility of this approach for assessment of antimalarial efficacy has been augmented by the implementation of a rapid, sensitive and robust real-time PCR assay for quantification of parasitaemia (5). A significant benefit of the IBSM model for *P. vivax* studies is the exclusion of the liver stage of the parasite that is the source of relapses, thus removing risk of recurring *P. vivax* infection from occult hypnozoites.

In the absence of a method of *in vitro* culture of *P. vivax*, the only way to source parasites is *ex vivo*. This hampers all aspects of development of tools to eliminate this parasite. For example, to test and develop candidate hypnozoiticidal drugs, a reliable source of *P. vivax* sporozoites is required. Currently this entails an expensive, logistically complex and unreliable process of sourcing *P. vivax*-infected mosquitoes from endemic areas of Asia. In addition to the logistic issues, the parasites are not genetically homogenous.

Recently we have been successful in transmitting *P. vivax* to mosquitoes and harvesting sporozoites. In a recent study we pooled 75 mL blood from 6 subjects, concentrated parasites by density gradient centrifugation and fed the concentrate to 6 pots of 100 *Anopheles Stephensi* mosquitoes (ANZCTR reference ID: ACTRN12616000174482). Approximately 95% of fed mosquitoes were infected; an average yield of approximately 4,000 sporozoites per salivary gland was obtained when mosquitoes were dissected 14 days later. Although this system offers great potential to study transmission biology, ethical and logistic issues will preclude it from becoming a source of sporozoites for downstream work.

Apheresis is the removal of a specific component of an individual’s blood with the remainder of the blood being returned to the individual. Apheresis may involve the removal of red cells (erythrocytapheresis), white cells (leukocytapheresis), plasma (plasmapheresis) or platelets (thrombocytapheresis). Currently centrifugal apheresis is the preferred method whereby blood components can be separated based on bouyancy. Centrifugal machines use citrate to prevent clotting of extracorporeal blood, thereby minimising the bleeding risk; they pack red cells to a haematocrit of ≥80%, therefore requiring smaller blood volumes to achieve extraction and enable peripheral veins to be used (6).

Since its introduction in the 1970s, apheresis has been used to treat a wide array of conditions, including essential thrombocythaemia, familial hypercholesterolaemia, graft versus host disease and babesiosis. Parasite apheresis has also been used to reduce Loa loa parasitemia, to prevent post-treatment encephalopathy (7-9). Until now apheresis in malaria has been limited to the context of exchange blood transfusion and red cell exchange as adjuncts to intravenous quinine therapy for severe *P. falciparum*  malaria (parasitemia >5%), as per WHO recommendations prior to the availability of IV artesunate (10). The rapid parasite clearance resulting from artesunate therapy has resulted in exchange transfusion and red cell exchange falling out of favour as a treatment for malaria.

## 2.2 Rationale

We propose to carry out a exploratory study to assess the use of apheresis as a means to harvest and concentrate all stages of *P. vivax* including gametocytes from up to 8 human subjects infected with blood stage *P. vivax* malaria. The harvested parasites would then be cryopreserved; the asexual parasites could then be used to produce a human malaria parasite (HMP) bank for use in future IBSM model studies, while the gametocytes could be used to infect *Anopheles* mosquitoes. Previous work has established that cryopreserved *P. vivax* gametocytes are infectious to mosquitoes (11). These infected mosquitoes, or sporozoites derived from them, could be used to infect healthy subjects in radical cure challenge models. Additionally, these sporozoites could be used in *in vitro* microfluidic devices that sustain human hepatocyte culture (12), as well as in humanised mouse models such as the severe combined immunodeficiency (SCID) mouse model which has shown the potential of acting as a model for the hepatic stage of malaria infection (13). Furthermore, it would transform *P.vivax* mosquito membrane feeding assays, obviating the need for a gametocytaemic test subject. We estimate that in collecting the entire parasite population from up to 5L of a subject’s blood by apheresis there would be sufficient gametocytes to feed ~400 pots of 100 mosquitoes. From a total of ~40,000 mosquitoes the theoretical yield would be ~3.2 x10^7^ sporozoites.

This explorative study has been designed to establish for the first time apheresis as a method of acquiring purified *P. vivax* parasites from healthy human subjects infected with IBSM. Venesection (whole blood removal using large bore cannula) cannot be used to select a specific heamatocrit and therefore purified parasites cannot be sourced as efficiently as through apheresis, hence our decision to use apheresis.

Hypotheses:

- The apheresis procedure is safe in healthy subjects experimentally infected with blood stage *P. vivax.*
- Apheresis can be used to extract and concentrate all stages of *P. vivax* parasites at numbers greater than can be attained by simple blood draws.
- Parasites harvested via apheresis can be successfully cryopreserved.
- Cryopreserved gametocytes can successfully be used to infect *Anopheles* mosquitoes.
- Asexual parasites extracted by apheresis can be used to produce a HMP bank for future IBSM model studies.
- Peripheral blood mononuclear cells (PBMCs) can be collected using apheresis.

## 2.3 Potential Risks and Benefits

### 2.3.1 Known Potential Risks

**Apheresis**

Apheresis is generally considered to be a safe and well tolerated method of treatment as well as a method for collecting blood component donations from healthy volunteers. Apheresis in the form of automated red blood cell exchange has been used as an adjunct to anti-malarial treatment, particularly during the quinine era to remove *P. falciparum* infected red cells. During review of the litereature we identified 42 patients treated with red cell exchange as adjunct therapy, the only significant adverse effect experienced as a result of automated red cell exchange was transient hypotension (blood pressure 70/40) which resulted in halting of apheresis for 10 minutes before starting again once the hypotension had resolved (25-39). In the current study, subjects will have far lower parasitaemia than that seen in severe malaria. Furthermore, subjects will be infected with blood stage *P. vivax* which is generally considered one of the benign malarias. There is no documented use of apheresis in *P.vivax* infection, but the good safety profile evident in subjects with severe *P.falciparum* infection suggests that this procedure should be well tolerated by healthy subjects with subclinical *P. vivax* infection. However, it is important to note that we will be carrying out a Continuous Mononuclear Cell (CMNC) procedure on our subjects (this is the procedure typically performed to collect stem cells and lymphocytes) which is different to automated red cell exchange, although both procedures fall under the umbrella of apheresis. Leukapheresis has been used successfully to aquire PBMCs from subjects immunised with radiation-attenuated *P. falciparum* sporozoites via mosquito bite prior to malaria challenge (40). PBMCs have been extracted via leukapheresis from healthy volunteers in order to study differernces in immune responses to *P. vivax* between duffy positive and duffy negative individuals and were subsesquently cryopreserved (41). The potential for minor transient complications is documented below.

The Australian haemovigilance report published in 2016 states that during 2013-2014 they recorded over 518,000 apheresis donations (plasmapheresis and plateletpheresis), the total rate of adverse events was 212 per 10,000 apheresis procedures (42). There were no associated deaths. The most common adverse events associated with plasmapheresis were vasovagal (65/10,000), haematoma and bruising (13/10,000). The vast majority of these adverse events were mild in severity. Adverse events requiring hospitalistaion were 2/10,000 and 4/10,000 for plasmapheresis and plateletpheresis respectively. Plateletpheresis is generally considered to have greater complication rates due to the greater need for anti-coagulation and longer duration of the procedure. The vast majority of these hospital attendances were brief and uneventful requiring no treatment. With the exception of citrate reactions which are known to be more common in CMNC procedures, other adverse events are thought to be similar to other forms of apheresis.

Risk management of citrate reactions

Citrate reactions are probably the most likely adverse events that may occur during the apheresis procedure. They are a result of low blood calcium levels caused by the anticoagulant citrate commonly used instead of heparin for apheresis due to its lower tendency to cause bleeding and its short half-life. Citrate reactions can occur in around 48% (43) of CMNC produces, the vast majority of which present as mild tingling around the mouth, nose, ears, fingers and toes (44). Other symptoms of mild citrate reactions include sneezing, headache, shivers, and mild drops in blood pressure. Very occasionally citrate reactions can cause anxiety, irritability, abdominal discomfort, nausea and vomiting, spasms of muscles of the hands and feet and more significant drops in blood pressure. Very rarely citrate reactions may be severe and can manifest with seizures and abnormal heart rhythms. Subjects will be supervised during the apheresis procedure by nursing staff experienced in the delivery of apheresis, and if any of the above symptoms occur the subject will be given calcium replacement which usually results in a rapid resolution of symptoms and halting of further progression. To reduce the chances of citrate reactions we will only include subjects in the trial who have no history of low blood calcium levels and with normal calcium levels on screening blood tests. If the subject develops signs or symptoms of low calium levels during apheresis they may be advised verbally to commence a high calcium diet for 24 hours post apheresis. This is in line with routine management of citrate reactions within the apheresis unit. The decision to commence a high calcium diet will be made by the apheresis specialist staff and the exact nature of that high calcium diet will also be determined by the apheresis specialist staff in conjunction with the subject (e.g. avoid milk if lactose intolerant).

Risk management of fainting and pre-fainting

Apheresis is an isovolaemic procedure hence there is thought to be little if any physiological challenge to the donor as a result of the procedure (45) hence syncope and presyncope is less common. Evidence from the United States suggests that double red cell and platelet donation have reduced instances of syncope compared with whole blood donation (46). Vasovagal reactions causing fainting can occur during or after donation sometimes up to 8 hours after donation. Events that occur in the donation center are termed immediate whereas those that occur after donation are termed delayed. A specialist apheresis nurse will be present throughout the procedure and in the event of pre-faint or faint they will act according to their standard protocols (see Appendix 3) e.g. by halting the apheresis procedure temporarily or permanently. Patients most commonly experience fainting and pre-fainting during venous cannulation often because of difficult venous access. We will reduce the liklihood of this by only including subjects with no previous history of syncope as a result of venous cannulation or blood taking and good venous access. Furthermore, we will avoid blood draws from veins located in the ante-cubital fossa in the lead up to apheresis in order to preserve the veins. Venous cannulation will be carried out by experienced staff. Delayed events are much less common than immediate reactions. In the unlikely event of a delayed event, the subject will be under medical supervision in confinement and thus will be managed appropriately. We will be initiating apheresis 8-12 days after inoculation of *P. vivax* at which point the subject is likely to have symptoms similar to a mild/moderate “flu like” illness. The symptoms tend to be well controlled by simple analgesia such as paracetamol (the use of ibuprofen immediately prior to apheresis is not recommended due to potential for altered platelet function). In general these subjects will be far more healthy than those with severe *P. falciparum*  malaria requiring therapeutic apheresis, which evidence suggests is generally well tolerated.

Risk management of bruising and haematoma during venous cannulation

The risk of bruising and haematoma is approximately 13/10,000 procedures, the vast majority of these are mild in nature and require no intervention. Low platelet counts commonly occur in malaria and may increase the risk of bleeding and haematoma. However, apheresis procedures have been done successfully with very low platelet counts. We will be using peripheral venous access as it is less invasive thus safer and the more commonly used method of access. We will reduce the chances of bruising and heamatoma by only including subjects with good venous access and we plan to avoid blood draws from the veins in the ante-cubital fossa in the lead up to apheresis in order to preserve the veins. Venous cannulation will be carried out by experienced staff. The subject will not be permitted to take ibuprofen 48 hours before apheresis.

Risk management of haemaglobin drop

Statistical analysis of the trends in haemoglobin during *P. vivax* IBSM studies at QIMR Berghofer have shown on average the haemoglobin drop by 10 days post inoculation is 3 g/L and the average maximum fall in haemoglobin at any point during the study is 13 g/L with a standard deviation of 7 g/L. We estimate a maximum drop of 20% in haemoglobin from apheresis, this approximates to 30 g/L. Thus, the maximum anticipated fall of around 43 g/L will leave a haemoglobin of 92 g/L, alevel well above the cut-off for transfusion.

General risk management procedures

There have been cases of air emboli (air bubbles in the blood) occurring as a result of apheresis. However, modern machines have a built in alarm that can detect air emboli. There is also the possibility of machine malfunction although this is rare, but if it occured it may mean that some of the blood that was removed may not be returned. This is unlikely to cause any significant problems to the subject.

The apheresis procedure will be supervised by experienced and trained staff in the delivery of apheresis. A specialist apheresis nurse will be present throughout the procedure. In addition one of our trial doctors experienced in malaria management will be present during the procedure or contactable by phone and available at the site of apheresis within 10 minutes in case of any concerns. Furthermore, the apheresis unit will have a haematology doctor/consultant within easy access in the event of any other issues. The haematology staff will retain the right to stop the apheresis procedure at any time as per their discretion. Time on the apheresis machine will be approximately 2-4 hours. The subject may have to remain in the apheresis unit slightly longer to allow connection to the apheresis apparatus.

**Induced blood stage malaria**

In this study, a cryopreserved inoculum containing *P. vivax* isolate HMPBS02-*Pv*  will be used. The *P. vivax* parasite HMPBS02-*Pv* bank has been previously used to inoculate 34 malaria study subjects using the IBSM model (24). No SAEs related to the challenge inoculum were reported in the subjects exposed to date.

Risk management of blood borne infections

Overall, the risk of infection from a possible blood borne virus from the blood transfused in this study would be expected to be very low for a number of reasons. First, the donor was screened and tested negative for presence of acute blood borne infections. Furthermore, the volume of blood used in the IBSM model for transmitting malaria is significantly lower than in a transfused unit, thus reducing the risk for transfusion reaction. The white cells are removed from the master cell bank during the production process, which lowers the risk of Epstein-Barr virus (EBV) and Cytomegalovirus (CMV) infection due to transfusion. The bank tested PCR negative for both CMV and EBV viruses even though the donor of the bank was antibody positive for both CMV and EBV.

As part of the safety monitoring, all study subjects will have serum stored for testing of blood-borne virus infections before entry and after the completion of the study.

Risk management of reaction to the blood sample

The malaria inoculum contains a small number of red cells from the original donor. The risk for development of red cell antibodies in this study is considered extremely low. The donor of the malaria cell bank used in this study was confirmed to be blood group O, Rh (D) positive; people with blood group O are generally considered “universal donors”, as recipients of their blood have minimal risk of developing red cell alloantibodies when given much larger volumes of blood than is envisaged for the process routinely used in the IBSM model. However, it is possible that subjects could suffer a transfusion reaction after they receive the inoculum, or develop antibodies to the donor red blood cells that may make blood transfusion more difficult in the future. Subjects will also be monitored for signs and symptoms in the period immediately after the administration of the malaria parasite dose and screened at the end of the study for occurrence of red cell alloantibodies as part of the safety monitoring.

Risk management of malaria infection

The number of blood stage parasites used to infect the study subjects in this study is much lower than that which reaches the blood after the bite of a single malaria-infected mosquito, where approximately 30,000 parasites are released into the blood when they break out of a single infected liver cell. For this study, following administration of the challenge inoculum, the growth of the parasites as well as any symptoms in the subjects will be closely monitored. The threshold for commencement of treatment will be when parasitaemia is ≥ 20,000 parasites/mL or the clinical symptom score is >6 (then treatment will begin within a 24 h period from notification) or at the Investigator’s discretion. This treatment threshold has been selected because it is below the point at which advanced and severe clinical symptoms of malaria infection are likely to occur. Furthermore, treatment may be started before the threshold is met by the Investigator at their discretion.

The parasites used in the challenge inoculum for this study are known to be sensitive to the standard anti-malarial drugs artemether/lumefantrine and Chloroquine. As such, there is no serious risk of clinical malaria providing that the inoculated subjects comply with the curative anti-malarial regimen as directed by the Investigator.

Risk management of liver function derangements

Transient, asymptomatic liver function test (LFT) derangements have been reported in several subjects in IBSM studies (19, 23, 47,48). These LFT derangments which consisted of ALT/AST elevation with no change in bilirubin did not require treatment, and resolved by the end of the studies. Following an independent review involving drug-induced liver injury experts, it was found that these LFT elevations, are most likely a direct consequence of the malaria infection rather than reflecting a direct drug-induced liver injury caused by an investigational antimalarial drug. As a precaution all subjects in this study will undergo regular safety monitoring to assess for asymptomatic LFT abnormalities. Subjects are required to reduce intake of possibly hepatotoxic substances during the course of the study including alcohol and paracetamol (paracetamol can be used as second line symptom control after ibuprofen and will not exceed 4g/day in the study).

**Antimalarial rescue medications**

Artemether/lumefantrine, chloroquine, and artesunate risks are detailed in their respective approved manufacturer’s prescribing information (Appendix 4).

Mosquito feeding

Subjects will be continuously monitored during the direct feeding of mosquitoes. If a participant develops severe skin sensitivity or experiences discomfort through the mosquito feed process, they will be given the option to withdraw from this process. Treatment for the skin irritation will be provided.

In summary, the risk to subjects in this study will be minimised as follows:

- Adherence to the inclusion/exclusion criteria, specific contraception rules for subjects.
- Close clinical and laboratory monitoring to ensure the safety and wellbeing of the healthy subjects.
- Monitoring and management by specialist staff trained in the delivery of apheresis, haematology team can stop apheresis at any point at their discretion.
- Only subjects without a history of low blood calcium and with normal calcium levels on screening blood tests will be included.
- Subjects will be advised to have a high calcium diet in the 24 hours prior to apheresis.
- Subjects will not be allowed to utilise ibuprofen in the 48 hours prior to apheresis.
- Paracetamol ≤ 4g/day
- Subjects with good venous access will be selected and ante cubital fossa veins will not be used for blood sampling prior to apheresis.
- Admission to clinical unit for at least 48 h following the apheresis procedure and initiation of anti-malarial treatment.
- If subject requires hospitalisation this will be done at the Infectious Diseases Unit, Royal Brisbane and Women’s Hospital.
- A Safety Review Team will meet approximately 4 weeks after the inoculation of each subject to review safety data before proceeding with the inoculation of a subsequent subject.

### 2.3.2 Known Potential Benefits

There are no known direct benefits to the subjects in this study. There may be a benefit to the subjects from the results of the screening tests and procedures (blood tests, physical examination and electrocardiogram).

# 3 OBJECTIVES AND PURPOSE

Primary objectives

- To further assess the safety of the *P. vivax* induced blood stage malaria (IBSM) model following inoculation of healthy subjects with *P. vivax* isolate HMPBS02-*Pv*.
- To assess the safety of apheresis in the *P. vivax* IBSM model following inoculation of healthy subjects with *P. vivax* isolate HMPBS02-*Pv*.

Secondary objectives

- To assess the feasibility of apheresis as a method of extracting and concentrating all stages of malaria parasites following inoculation of healthy subjects with *P. vivax* isolate HMPBS02-*Pv*.
- To evaluate the success of cryopreservation of all stages of *P. vivax* parasites from blood products extracted via apheresis from healthy subjects inoculated with. *P. vivax* isolate HMPBS02-*Pv*.

Exploratory

- To explore the potential for apheresis to be used as a method for producing a *P. vivax* Human Malaria Parasite (HMP) bank to be used for future IBSM studies.
- To evaluate the transmission of *P. vivax* gametocytes to mosquitoes.

Exploratory optional

- To collect and store plasma and peripheral blood mononuclear cells (PBMCs) harvested using apheresis for future research on the immunology and pathophysiology of *P.vivax*.
- To assess development and/or functionality of antibodies to asexual stage parasites and/or gametocytes.

# 4 STUDY DESIGN AND ENDPOINTS

## 4.1 Description of the Study Design

This is a Phase 1 exploratory study designed to determine the safety and feasibility of using apheresis as a method for extracting all lifecycle stages of malaria parasites from the blood of healthy subjects experimentally infected with blood stage *P. vivax*. This study will be conducted in up to 8 subjects (8 cohorts of 1 subject each). Approximately 4 weeks after the inoculation of each subject, the safety review team (SRT) will meet to review the progress of the subject before going forward with the inoculation of a subsequent subject.

Subjects will be consenting and eligible healthy adults males, aged between 18-55 years old. Subjects will be inoculated intravenously on Day 0 with approximately 1100 viable *P. vivax* HMPBS02-*Pv* parasite-infected erythrocytes.

On an outpatient basis, subjects will be monitored daily via phone and then will attend the clinical unit daily from 4 days post-inoculation for blood sampling to measure parasitaemia via qPCR targeting the *P. vivax* 18S rRNA gene (referred to as malaria 18S qPCR), to monitor symptoms and signs of malaria, and to record AEs.

The threshold for the commencement of apheresis and subsequent antimalarial rescue treatment with artemether/lumefantrine will occur when parasitaemia is >20,000 parasites/mL or the Malaria Clinical Score is >6 (within 24 hours of notification) or at the Investigator’s discretion. Within 24h of the day that this threshold is reached (expected to occur on Day 9, 10 or 11), subjects will be admitted to the clinical unit (expected on day 10, 11 or 12) for initial safety assessments before being escorted to the apheresis unit in the Cancer Care Services by Q-pharm staff. The subject will then undergo the apheresis procedure as per the Standard Operating Procedure (SOP) (see Appendix 3) whilst being supervised by the apheresis specialist nurse (the apheresis procedure is expected to take 2-4 hours). The subject will then be escorted back to the clinical site and administered the first dose of artemether/lumefantrine. The subject will remain confined within the clinical unit for 72 hours (or less - 48h minimum- at the discretion of the investigator if the subject is deemed clinically well and the subject is happy for early release) to monitor for safety and tolerability of apheresis and rescue therapy, and to ensure adequate clinical and parasitological response to treatment. In the unlikely event that artemether/lumefantrine fails to clear parasitaemia, subjects will be treated with chloroquine. If oral administration of either artemether/lumefantrine or chloroquine is not possible (eg. the subject is vomiting), the subject will receive intravenous treatment with artesunate. After discharge from the clinical unit, subjects will be followed up on an out-patient basis for monitoring of safety and parasite clearance. Follow-up for safety assessments will be performed on Day 28±3, Day 56±7 (phone call only), and Day 90±7(End of Study).

Subjects will also be evaluated for the presence of sexual parasite stages (gametocytes) and other parasite lifecycle stages in the blood during the study using reverse transcriptase qPCR (qRT-PCR). Parasite lifecycle stage qRT-PCR will also be used at the Investigator’s discretion to determine the timing of malaria transmission experiments; direct feeding assays (DFAs) and/or membrane feeding assays (MFAs) which may occur at up to 2 time-points prior to apheresis and rescue treatment initiation.

## 4.2 Study Endpoints

### 4.2.1 Primary Endpoint

The primary objectives will be assessed by adverse events (AE) recording, safety laboratory parameters, vital signs recording, electrocardiograms (ECGs), physical examination findings and Malaria Clinical Score recording.

### 4.2.2 Secondary Endpoints

The secondary objectives will be assessed as follows:

- The feasibility of apheresis as a method of extracting and concentrating all stages of malaria parasites will be determined by qPCR, qRT-PCR, microscopy and flow cytometry on blood products extracted using apheresis, and expressed as a percentage of baseline parasitaemia (pre-apheresis).
- The success of cryopreservation of gametocytes will be determined by measuring their infectivity to mosquitoes using a membrane feeding assay, reported as prevalence of infection (percentage of oocyst positive mosquitoes). The success of cryopreservation of asexual parasites will be determined based on their viability tested using *in vitro* re-invasion assay.

### 4.2.3 Exploratory Endpoints

The exploratory objectives will be assessed as follows:

- The potential for apheresis to be used as a method for producing a *P. vivax* HMP bank will be determined based on the efficiency of extraction and concentration of parasites, the viability of extracted parasites, the successful leukodepletion of the sample, and the confirmation that the donor subject is free from blood-borne viruses.
- Transmission of *P. vivax* gametocytes to mosquitoes will be determined using a direct feeding assay and/or membrane feeding assay, and reported as prevalence of infection (percentage of oocyst positive mosquitoes).

### 4.2.4 OPTIONAL Exploratory Endpoints

The optional exploratory objectives will be assessed as follows:

- The successful collection of PBMCs will be determined by cell counts and cell viability tested using trypan blue staining.
- Detection and evaluation of anti-blood-stage or anti-gametocyte specific antibodies using immunological assays (including but not limited to enzyme-linked immunosorbent assays and indirect immunofluorescence assays).

# 5 STUDY ENROLLMENT AND WITHDRAWAL

## 5.1 Subject Inclusion Criteria

Subjects eligible for inclusion in this study must fulfil **all** of the following criteria:

**Demography**

I01. Adult male subjects between 18 and 55 years of age, inclusive who do not live alone (from Day 0 until at least the end of the anti-malarial drug treatment) and will be contactable and available for the duration of the trial and up to 2 weeks following end of study visit.

I02. Body mass index between 18.0 and 32.0 kg/m^2^, inclusive and a minimum body weight of 50 kg.

**Health status**

I03. Certified as healthy by a comprehensive clinical assessment (detailed medical history and complete physical examination).

I04. Vital signs after 5 minutes resting in supine position:

- 90 mmHg ≤ systolic blood pressure (SBP) ≤ 140 mmHg,
- 50 mmHg ≤ diastolic blood pressure (DBP) ≤ 90 mmHg,
- 50 bpm ≤ heart rate (HR) ≤ 100 bpm.

I05. Normal standard 12-lead electrocardiogram (ECG) after 5 minutes resting in supine position, QTcF≤450 ms with absence of second or third degree atrioventricular block or abnormal T wave morphology.

I06. Laboratory parameters within the normal range, unless the Investigator considers an abnormality to be clinically irrelevant for healthy subjects enrolled in this clinical investigation in accordance with approved clinically acceptable laboratory ranges documented prior to study start. More specifically for serum corrected calcium, creatinine, hepatic transaminase enzymes (aspartate aminotransferase, alanine aminotransferase), and total bilirubin (unless the participant has documented Gilbert syndrome) should not exceed the approved acceptable ranges and haemoglobin must be equal or higher than the lower limit of normal.

I07. As there is the risk of adverse effects with artemether/lumefantrine and chloroquine in pregnancy, it is important that any subjects involved in this study do not get pregnant.

I08. All subjects must be Duffy Blood group positive and have blood type O.

**Contraception**

Male subjects must agree to use a double method of contraception including condom plus diaphragm or condom plus stable oral/transdermal/injectable hormonal contraceptive by female partner during the study.

Abstinent heterosexual male subjects must agree to start a double method if they start a sexual relationship during the study.

Adequate contraception does not apply to male subjects with same sex partners, provided they agree to start a double barrier method if they start a sexual relationship with a female during the study.

Adequate contraception rules do not apply to males who have undergone a vasectomy and had testing to confirm the success of the vasectomy.

Adequate contraception rules do not apply to males with female partners who are not of child bearing potential (e.g. due to total hysterectomy) and this female is the sole partner for that participant.

Adequate contraception does not apply to subjects of childbearing potential with same sex partners (abstinence from penile-vaginal intercourse), when this is their preferred and usual lifestyle.

**Regulations**

I010. Having given written informed consent prior to undertaking any study-related procedure.

## 5.2 Subject Exclusion Criteria

Subjects fulfilling **any** of the following criteria will not be eligible for inclusion in this study:

**Medical history and clinical status**

E01. Any history of malaria or participation in a previous malaria challenge study.

E02. Any history of retinal abnormalities, disease of the retina or macula of the eye, visual field defects, hearing disorders (e.g. reduced hearing, tinnitus).

E03. Must not have travelled to or lived (>2 weeks) in a malaria-endemic region during the past 12 months or planned travel to a malaria-endemic region during the course of the study (for endemic regions see http://www.map.ox.ac.uk/browse-resources/).

E04. Has evidence of increased cardiovascular disease risk (defined as >10%, 5 year risk for those greater than 35 years of age, as determined by the Australian Absolute Cardiovascular Disease Risk Calculator (<http://www.cvdcheck.org.au/>). Risk factors include sex, age, systolic blood pressure (mm/Hg), smoking status, total and HDL cholesterol (mmol/L), and reported diabetes status.

E05. History of splenectomy.

E06. Presence or history of drug hypersensitivity, or allergic disease diagnosed by an allergist/immunologist and/or treated by a physician for allergy or history of a severe allergic reaction, anaphylaxis or convulsions following any vaccination or infusion.

E07. Presence of current or suspected serious chronic diseases such as cardiac or autoimmune disease (HIV or other immuno-deficiencies), insulin-dependent and non-insulin dependent diabetes, progressive neurological disease, severe malnutrition, acute or progressive hepatic disease, acute or progressive renal disease, porphyria, psoriasis, rheumatoid arthritis, asthma, epilepsy, or obsessive compulsive disorder.

E08. History of malignancy of any organ system (other than localised basal cell carcinoma of the skin or *in situ* cervical cancer), treated or untreated, within 5 years of screening, regardless of whether there is evidence of local recurrence or metastases.

E09. Subjects with history of schizophrenia, bi-polar disease, or other severe (disabling) chronic psychiatric diagnosis including depression or receiving psychiatric drugs or who has been hospitalised within the past 5 years prior to enrolment for psychiatric illness, history of suicide attempt, or confinement for danger to self or others.

E10. History of serious psychiatric condition that may affect participation in the study or preclude compliance with the protocol, including but not limited to past or present psychoses, disorders requiring lithium, a history of attempted or planned suicide, more than one previous episode of major depression, any previous single episode of major depression lasting for or requiring treatment for more than 6 months, or any episode of major depression during the 5 years preceding screening.

The Beck Depression Inventory (Appendix 5) will be used as an objective tool for the assessment of depression at screening. In addition to the conditions listed above, subjects with a score of 20 or more on the Beck Depression Inventory and/or a response of 1, 2 or 3 for item 9 of this inventory (related to suicidal ideation) will not be eligible for participation. These subjects will be referred to a general practitioner or medical specialist as appropriate. Subjects with a Beck score of 17 to 19 may be enrolled at the discretion of the Investigator if they do not have a history of the psychiatric conditions mentioned in this criterion and their mental state is not considered to pose additional risk to the health of the volunteer or to the execution of the study and interpretation of the data gathered.

E11. Frequent headaches and/or migraines, recurrent nausea, and/or vomiting (more than twice a month).

E12. Presence of acute infectious disease or fever (e.g., sub-lingual temperature ≥38.5°C) within the 5 days prior to inoculation with malaria parasites.

E13. Evidence of acute illness within the 4 weeks prior to screening that the Investigator deems may compromise participant safety.

E14. Significant inter-current disease of any type, in particular liver, renal, cardiac, pulmonary, neurologic, rheumatologic, or autoimmune disease by history, physical examination, and/or laboratory studies including urinalysis.

E15. Participant has a clinically significant disease or any condition or disease that might affect drug absorption, distribution or excretion (e.g. gastrectomy, diarrhoea).

E16. Participation in any investigational product study within the 12 weeks preceding the study.

E17. Blood donation, any volume, within 1 month before inclusion, or participation in any research study involving blood sampling (more than 450 mL/unit of blood), or blood donation to the Australian Red Cross Blood Service (Blood Service) or other blood bank during the 8 weeks preceding the treatment drug dose in the study.

E18. Participant unwilling to defer blood donations to the Blood Service for at least 6 months.

E19. Medical requirement for intravenous immunoglobulin or blood transfusions.

E20. Participant who has ever received a blood transfusion.

E21. Symptomatic postural hypotension at screening, irrespective of the decrease in blood pressure, or asymptomatic postural hypotension defined as a decrease in systolic blood pressure ≥20 mmHg within 2-3 minutes when changing from supine to standing position. In the event of a single asymptomatic postural hypotension reading the Investigator may repeat the reading once only at their discretion.

E22. Previous history of syncope or presyncope during blood donation or blood taking.

E23. History or presence of alcohol abuse (alcohol consumption more than 4 units per day) or drug habituation, or any prior intravenous usage of an illicit substance.

E24. Smoking more than 5 cigarettes or equivalent per day and unable to stop smoking for the duration of confinement. Subjects may smoke up to 5 cigarettes or equivalent per day for the rest of the study.

E25. Ingestion of any poppy seeds within the 24 hours prior to the screening blood test (subjects will be advised by phone not to consume any poppy seeds in this time period).

E26. Excessive consumption of beverages or food containing xanthine bases, including Red Bull, chocolate etc., more than 400 mg caffeine per day (equivalent to more than 4 cups per day).

**Interfering substance**

E27. Any vaccination within the last 28 days.

E28. Any corticosteroids, anti-inflammatory drugs, immunomodulators or anticoagulants. Any participant currently receiving or having previously received immunosuppressive therapy, including systemic steroids including adrenocorticotrophic hormone (ACTH) or inhaled steroids in dosages which are associated with hypothalamic-pituitary-adrenal axis suppression such as 1 mg/kg/day of prednisone or its equivalent or chronic use of inhaled high potency corticosteroids (budesonide 800 μg per day or fluticasone 750 μg).

E29. Any recent (<6 weeks) or current systemic therapy with an antibiotic or drug with potential anti-malarial activity (i.e. chloroquine, piperaquine, benzodiazepine, flunarizine, fluoxetine, tetracycline, azithromycin, clindamycin, doxycycline etc.).

**General conditions**

E30. Any participant who, in the judgment of the Investigator, is likely to be noncompliant during the study, or is unable to cooperate because of a language or mental deficit.

E31. Any participant in the exclusion period of a previous study according to applicable regulations.

E32. Any participant who is the Investigator or any sub-investigator, research assistant, pharmacist, study coordinator, or other staff thereof, directly involved in conducting the study.

E33. Any participant without a good peripheral venous access.

**Biological status**

E34. Positive result on any of the following tests: hepatitis B surface (HBs Ag) antigen, anti-hepatitis B core antibodies (anti-HBc Ab), anti-hepatitis C virus (anti-HCV) antibodies, anti-human immunodeficiency virus 1 and 2 antibodies (anti-HIV1 and anti HIV2 Ab), syphilis (EIA).

E35. Positive urine drug test for any drug listed in Section 7.2.1 (Drug Screening) unless there is an explanation acceptable to the Investigator (e.g., the participant has stated in advance that they consumed a prescription or over-the-counter product which contained the detected drug) and/or the participant has a negative urine drug screen on retest by the pathology laboratory. Any participant testing positive for acetaminophen (paracetamol) at screening may still be eligible for study participation, at the Investigator’s discretion.

E36. Positive alcohol breath test.

**Specific to the study**

E37. Cardiac/QT risk:

- Family history of sudden death or of congenital prolongation of the QTc interval or known congenital prolongation of the QTc interval or any clinical condition known to prolong the QTc interval.
- History of symptomatic cardiac arrhythmias or with clinically relevant bradycardia.
- History of electrolyte disturbances, particularly hypokalaemia, hypocalcaemia, or hypomagnesaemia.
- Electrocardiogram (ECG) abnormalities in the standard 12-lead ECG (at screening) which in the opinion of the Investigator is clinically relevant or will interfere with the ECG analyses on study.

E38. Known hypersensitivity to artemether/lumefantrine or chloroquine or any of thier excipients, or 4-aminoquinolines, artemether or other artemisinin derivatives, lumefantrine, piperaquine.

E39. Known severe reaction to mosquito bites other than local itching and redness.

E40. Unwillingness to abstain from consumption of grapefruit or Seville orange from initiation of the study (Day 0 until completion of antimalarial treatment).

E41. Unwillingness to abstain from consumption of quinine containing foods/beverages such as tonic water, lemon bitter, from inoculation (Day 0) to the end of the antimalarial treatment.

E42. Use of prescription drugs or non-prescription drugs and herbal supplements (such as St John’s Wort), within 14 days or 5 half-lives (whichever is longer) prior to the inoculation administration. (Note: diazepam interferes with the analysis of blood levels of chloroquine and thus should not have been used for at least 8 weeks prior to administration of the study drug). If needed (i.e. an incidental and limited need) ibuprofen up to 1.2 g/day or paracetamol is acceptable up to 4 g/day. The subject must inform the investigator of any ibuprofen or paracentamol use at the next convenient time. Limited use of other non-prescription medications or dietary supplements not believed to affect subject safety or the overall results of the study, may be permitted on a case-by-case basis following approval by the Sponsor in consultation with the Investigator. Subjects are requested to refrain from taking non-approved concomitant medications from recruitment until the conclusion of the study.

E43. History of coagulopathy or bleeding diasthesis.

Subjects who are excluded from participation on study days for any of the above reasons may be eligible to participate on a postponed schedule if the Investigator considers this appropriate.

## 5.3 Strategies for Recruitment and Retention

A maximum of 8 subjects are planned to be enrolled. It is estimated that up to 20 subjects may need to be screened to complete enrolment.

No restrictions will apply for ethnic or racial categories. The expected population may include all Australian racial categories. Subjects will be recruited from the QIMR Berghofer Human Research Ethics Committee (QIMR Berghofer HREC) approved database of healthy subjects maintained by Q-Pharm, or by a general or study specific advertisement via print, radio or poster media to students of Queensland universities or to the general community, as approved by the QIMR Berghofer HREC.

Subjects who complete the study up to Day 90±7/EOS will be paid $2750 compensation for their participation. Subjects who withdraw or are withdrawn from the study will be compensated on a fractional basis for their involvement unless they are withdrawn as a consequence of their misconduct. Reserve subjects who do not participate in the study will be paid $150 compensation for the inconvenience associated with their attendance for screening and for their attendance on the inoculation day, in case they are required to participate. Subjects who are screened but are ineligible for the study will be paid up to $75 compensation for the inconvenience associated with their attendance for screening (unless there is evidence that the subject knowingly omitted information about their eligibility before screening e.g. positive urine drug screen or positive alcohol breath test etc.).

## 5.4 Subject Withdrawal or termination

5.4.1 Reasons for Withdrawal or Termination

Subjects are free to withdraw from the study at any time. A subject may be considered withdrawn if he/she states an intention to withdraw, fails to return for scheduled protocol visits for any reason, or becomes lost to follow-up. Subjects may also be withdrawn by the Investigator. Possible reasons for withdrawal by the Investigator include the occurrence of a serious adverse event (SAE), failure by the subject to comply with the requirements of the protocol, or for any other reason at the Investigator’s discretion.

5.4.2 Handling of Subject Withdrawals or termination

The Investigator will make every effort to determine the primary reason for a subject’s withdrawal from the study and record this information in the Case Report Form (CRF). For subjects who are lost to follow-up, the Investigator will demonstrate "due diligence" by documenting all steps taken to contact the subject (e.g. dates of telephone calls, home visit, etc.) in the source documents.

If the subject is withdrawn from the study procedures or follow-up for any reason, with the subject’s permission, medical care will be provided for any SAEs that occurred during participation in the study until the symptoms of any SAEs are resolved and the subject’s condition becomes stable. Follow-up for AEs is described in Section 8.3.

If earlier withdrawal from further study procedures occurs, the subject will be asked to complete the antimalarial treatment. The subjects will also be asked to complete the early termination evaluation as described in Section 7.3.5.

## 5.5 Premature Termination or Suspension of Study

The Sponsor, Principal Investigator, HREC and Regulatory Authorities independently reserve the right to discontinue the study at any time for safety or other reasons. This will be done in consultation with the Sponsor where practical. In the event of premature trial termination or suspension, the above-mentioned parties will be notified in writing by the terminator/suspender stating the reasons for early termination or suspension (with the exception of the Sponsor’s responsibility for notifying the Regulatory Authorities). After such a decision, the Sponsor and the Investigator will ensure that adequate consideration is given to the protection of the subjects’ interest and safety. The Investigator must review all subjects as soon as practical and complete all required records.

In addition to the classic assessment of SAEs and the occurrence/severity of other AEs by the Sponsor and the Investigator, after exploring potential confounding factors, the following criteria should be considered as guidance for the decision to stop inoculation of further subjects:

- A subject experiences an SAE that is related to the inoculum.
- The haematology team or apheresis specialist may decide to stop apheresis based on safety, at their discretion.
- The Investigator and Sponsor may decide to stop inoculation based on other safety signals not described in the above criteria.

# 6 STUDY AGENT

## 6.1 Study Agent(s) and Control Description

### 6.1.1 Acquisition

*P. vivax* HMPBS02-*Pv* challenge agent

The *P.vivax* HMPBS02-*Pv* MCB was derived from blood group O rhesus positive blood donated from a returned traveller from India who presented with clinical manifestations of malaria. The *P.vivax* HMPBS02-*Pv* MCB was cryopreserved, aliquoted into cryovials and stored under liquid nitrogen under controlled conditions. Refer to the *P.vivax* HMPBS02-*Pv* Investigator’s Brochure for more details. A *P.vivax* HMPBS02-*Pv* MCB cryovial will be retrieved from storage, thawed, and used to aseptically prepare the inoculum at Q-Gen.

Rescue medications

Artemether/lumefantrine (Riamet^®^) distributed by Novartis Pharmaceuticals Australia Pty Ltd, and chloroquine distributed by Alliance Pharmaceuticals Ltd will be acquired by Q-Pharm. Artesunate is the recommended parenteral treatment for malaria in Australia. Currently, it is a Special Access Scheme drug, and has been sourced from Guilin Pharmaceutical (Shanghai) Co., Ltd. Import was facilitated by Medicines for Malaria Venture. The manufacture of intravenous artesunate is undertaken in a WHO Pre-Qualified GMP facility (<http://www.mmv.org/access/access-portfolio/artesun-injectable-artesunate>).

### 6.1.2 Formulation, Appearance, Packaging, and Labeling

*P. vivax* HMPBS02-*Pv* challenge agent

Each inoculum dose will contain parasitised and unparasitised RBCs, resuspended in 0.9% Sodium Chloride Intravenous Infusion, in a total volume of 2 mL in syringes. The syringes will be double contained following preparation and labelled in accordance with GCP guidelines and the Access to Unapproved Therapeutic Goods - Clinical Trials in Australia (TGA, 2004) .

Rescue medications

Artemether/lumefantrine tablets are pale yellow, flat, round, uncoated tablets with bevelled edges, marked with N/C and a score line on one side and CG on the other, supplied in blister packs containing 16, 24, or 400 tablets. Chloroquine tablets are round, flat, white, uncoated tablets with a breakline on one side, supplied in a bottle containing 100 tablets. Artesunate for i.v administration is presented as a powder for reconstitution (60 mg artesunic acid) in a vial.

All rescue drugs will be labelled according to identity, brand or source, and batch number. The contents of the label for drug to be administered to the subjects will be in accordance with all applicable regulatory requirements.

### 6.1.3 Product Storage and Stability

*P. vivax* HMPBS02-*Pv* challenge agent

The malaria challenge agent is prepared at Q-Gen on inoculation day (Day 0). The time between preparation of the inoculum and administration to each subject will be a maximum of 1.5 hours, during which time the syringes will be kept at the required temperature as recorded on the label.

Rescue medications

- Artemether/lumefantrine: store below 30°C protected from moisture.
- Chloroquine: store below 30°C.
- Artesunate: store in tightly closed containers, protected from light.

All drugs will be held in appropriate locked storage conditions at the clinical unit until required.

### 6.1.4 Preparation

*P. vivax* HMPBS02-*Pv* challenge agent

The inocula will be prepared aseptically at Q-Gen (QIMR Berghofer) from a frozen cryovial of the *P. vivax* HMPBS02-*Pv* MCB by nominated QIMR Berghofer staff under the guidance of the Investigator. The infected erythrocytes will be thawed, washed, re-suspended in normal saline, diluted in a final volume of 2 mL of clinical grade saline, and dispensed into syringes. Any remaining unused infected RBCs will be discarded as per approved standard operating procedures.

Rescue medications

Artemether/lumefantrine, chloroquine are available as tablets and no preparation is required. Artesunate powder for reconstitution will be dissolved in 1 mL sodium bicarbonate (5%), then 5 mL sodium chloride (0.9%) will be added to create a 10 mg/mL solution (total volume 6 mL).

### 6.1.5 Dosing and Administration

*P. vivax* HMPBS02-*Pv* challenge agent

An inoculum dose, containing an estimated ~1100 viable *P. vivax-*infected erythrocytes in a volume of 2 mL, will be administered intravenously to each participant on the morning of Day 0. The actual number of parasites inoculated will take into account the loss of viability resulting from cryopreservation, storage and thawing. On inoculation day, subjects may have food until at least half an hour prior to inoculation. Subjects will undergo intravenous cannulation with an appropriate gauge cannula. Placement and patency will be checked by flushing the vein with 5-10 mL of clinical grade saline. The inoculum will be injected, and the cannula again flushed with 5-10 mL of clinical grade saline. The cannula will then be removed, and haemostasis ensured by use of an appropriate dressing. Following inoculation of the subjects, the parasite count of the inocula will be quantified by malaria 18S qPCR.

Rescue medications

**Artemether/lumefantrine**

All subjects will receive compulsory treatment with artemether/lumefantrine following apheresis directly after returning to Q-Pharm. The threshold for treatment will occur when parasitaemia is >20,000 parasites/mL or the Malaria Clinical Score is >6 or at the Investigator’s discretion. Artemether/lumefantrine tablets containing 20 mg artemether and 120 mg lumefantrine will be administered as 6 doses of 4 tablets (total course of 24 tablets) given over a period of 60 hours (total dose of 480 mg artemether and 2.88 g lumefantrine). Each dose of tablets administered orally should be taken with food or drinks rich in fat (e.g., milk). Subjects will be reminded of the potential side effects of artemether/lumefantrine (Riamet^®^) and given the Consumer Medicine Information for Riamet^®^ (Appendix 4).

**Chloroquine (only if required)**

Subjects will only be administered chloroquine if artemether/lumefantrine fails to clear the malaria parasites. For example, if qPCR results indicate unsatisfactory clearance of the parasitaemia, defined as 2 consecutive qPCR time-points showing less than 20% of baseline (i.e. the parasitaemia before drug treatment) by 72 hours, subjects may be administered chloroquine. The decision to institute early curative treatment will be made in consultation with the Local Independent Medical Monitor (IMM), who is an external malaria expert, to advise on the safety of continuing observation without rescue versus administration of curative treatment. Chloroquine tablets containing 250 mg chloroquine phosphate (equivalent to 155 mg chloroquine base) will be administered as an initial oral dose of 4 tablets, followed by 2 tablets at 6, 24, and 48 hours (total dose of 2.5 g chloroquine phosphate [1.550 g base]).

**Artesunate (only if required)**

Treatment of subjects with i.v. artesunate will only occur in the event that subjects are unable to complete oral treatment with either artemether/lumefantrine or chloroquine (e.g. the subject is vomiting). This would be done at the recommended dose regime of 2.4 mg/kg at approximately 0, 12, 24, hours and then daily for up to 7 days or until able to take oral drugs.

### 6.1.6 Route of Administration

Inoculum

Intravenous.

Rescue medications

- Artemether/lumefantrine: oral.
- Chloroquine (if required): oral.
- Artesunate (if required): intravenous.

### 6.1.7 Starting Dose and Dose Escalation Schedule

Dosing with the malaria challenge agent and rescue drugs is presented in Section 6.1.5. No dose escalation will be performed.

### 6.1.8 Dose Adjustments/Modifications/Delays

Not applicable.

### 6.1.9 Duration of Therapy

- Inoculum: single dose.
- Artemether/lumefantrine: 3 days.
- Chloroquine (if required): 3 days.
- Artesunate (if required): maximum of 7 days.

### 6.1.10 Tracking of Dose

The *P. vivax* challenge inocula, artemether/lumefantrine, chloroquine (if required) and artesunate (if required) will be administered at the clinical research Unit (CRU) in the presence of clinic staff.

## 6.2 Study agent Accountability Procedures

The Q-Pharm pharmacist or designee, as nominated by the Investigator, is responsible for

maintaining accurate study agent accountability records throughout the study. Study agents include the malaria challenge agent and the rescue medication. Dispensing, accountability and documentation will be in accordance with Q-Pharm standard procedures. All products will be inventoried upon receipt by the Q-Pharm pharmacist. The condition of the products at the time of receipt by the pharmacist will be documented, as will the time restrictions of use for the syringes containing the malaria challenge agent. The lot numbers and expiry dates of the inoculum and antimalarial drugs will be documented. The Q-Pharm pharmacist or delegate will ensure that the received products are the specified formulation.

The storage, handling and the disposal of the challenge agents will be in accordance with approved procedures. All dosages prescribed and dispensed to the subjects and all dose changes during the study must be recorded in the CRFs. All drug supplies are to be used only in accordance with this protocol, and not for any other purpose. All used medications will be fully documented. Used and unused drug containers must be destroyed at the site once drug accountability is final and has been checked by the Sponsor or its delegate, and written permission for destruction has been obtained from the Sponsor.

Study products and study accountability logs will be available to the Sponsor or their representative as part of the study monitoring procedures. Upon completion of the study, copies of all study drug management records will be provided to the Sponsor. Original records will be maintained at the clinical site with the rest of the study records.

# 7 STUDY PROCEDURES AND SCHEDULE

## 7.1 Study Procedures/Evaluations

### 7.1.1 Study specific procedures

Medical history

| Past Medical/Surgical History Includes: |
| --- |
| History of all known allergies |
| Current medications, including over-the-counter and herbal preparations |
| History of substance abuse and recreational drug use |
| History of depression, anxiety, mental illness, emotional problems, use of psychiatric medications and previous psychotherapy |
| Surgical procedures and results |

Physical examination

| Complete Physical Examination Includes: |
| --- |
| Weight **(Screening only)** |
| Height **(Screening only)** |
| Review of systems excluding genitourinary examination and including the following: |
| Head, neck (including thyroid), ears, eyes, nose and throat |
| Heart/circulation |
| Chest |
| Lungs |
| Abdomen |
| Skin |
| Neurological exam |

Symptom-Directed Physical Examination: physical examinations will be symptom-directed at specified time-points (i.e. systems will be reviewed only if clinically indicated at the discretion of the Investigator).

Beck Depression Inventory

All subjects will be required to complete the Beck Depression Inventory at screening. This is a validated questionnaire used as an objective tool for the assessment of depression (See Appendix 5).

Vital signs

Vital signs (temperature, heart rate, respiratory rate and blood pressure) will be measured at screening after the subject has rested in the supine position for at least 5 minutes and in the standing position within 2-3 minutes when changing from the supine to standing position (blood pressure and heart rate only). At all other time-points, vital signs will be measured after the subject has rested in the seated position for at least 5 minutes. Tympanic temperature will be taken at the clinical unit, and sublingual temperature will be taken by subjects at home for practical reasons.

Electrocardiograms

A single 12-lead ECG will be recorded after resting supine for at least 5 minutes.

Apheresis

Apheresis will be performed according to the SOP included in Appendix 3. This procedure is similar to the double red blood cell donation procedure used by the Council of Europe and the American Red Cross (49). 386 mL of red blood cells can be collected safely in subjects with normal hemoglobin and hematocrite levels according to this well-recognised procedure. Blood sampling

Blood will be collected for clinical laboratoratory evaluations including haematology, clinical chemistry, serology and pregnancy testing (see section 7.2.1). Blood samples will also be collected to monitor malaria parasitaemia and for immunology/pathophysiology research (see section 7.2.2). The estimated blood volume required for the these tests is listed in Appendix 1. The total volume of whole blood drawn from each adult male subject in addition to the apheresis procedure will not exceed 200 mL in any given 30-day period. This volume includes allowance for unscheduled safety laboratory assessments that may be required at the discretion of the Principal Investigator or the Sponsor to ensure subject safety. The subject haemoglobin count will be checked regularly in order to maintain levels above 120 g/L prior to apheresis and above 90 g/L after the apheresis procedure. Subjects with haemoglobin count below 135 g/L will be excluded from the study.

Urine sample collection

Urine will be collected for urinalysis and drug screening (see section 7.2.1).

Adverse event recording

Adverse events will be recorded as described in section 8 and follow the CTCAE V4.03 gradings.

Malaria Clinical Score

The following 14 signs/symptoms frequently associated with malaria will be graded using a 4-point scale (absent: 0; mild: 1; moderate: 2; severe: 3) and summed to generate a total Malaria Clinical Score (maximum score possible is 42). Individual scores for each symptom as well as the total score will be recorded.

| Headache | Anorexia |
| --- | --- |
| Myalgia (muscle ache) | Nausea |
| Arthralgia (joint ache) | Vomiting |
| Fatigue/lethargy | Abdominal discomfort |
| Malaise (general discomfort/uneasiness) | Fever |
| Chills/Shivering/Rigors | Tachycardia |
| Sweating/hot spells | Hypotension |

### 7.1.2 Standard of care study procedures

Not applicable.

## 7.2 Laboratory Procedures/Evaluations

### 7.2.1 Clinical Laboratory Evaluations

Haematology

| Full blood count (FBC) with differential |
| --- |
| White blood cell count (WBC) |
| WBC differential (diff) |
| A manual blood smear should be reviewed if there are immature/abnormal cells detected on the automated differential or if an automated differential was not able to be performed. |
| neutrophils (NEUT) |
| lymphocytes (LYM) |
| monocytes (MON) |
| eosinophils (EOS) |
| basophils (BAS) |
| Red blood cell count (RBC) |
| Haemoglobin (HGB) |
| Haematocrit (HCT) |
| Platelet count (PLAT) |
| Reticulocyte count (RETI) (Day -3 to -1 eligibility confirmation visit or at screening if between Day -3 to -1, and Day 28±3 or early termination visit only) |
| Blood Group and Rh(D) (Screening only) |
| Duffy antigen (Screening only) |

Biochemistry

| Sodium (SODIUM) | Alkaline phosphatase (ALP) |
| --- | --- |
| Potassium (K) | Alanine aminotransferase (ALT, SGPT) |
| Chloride (CL) | Aspartate aminotransferase (AST, SGOT) |
| Bicarbonate (BICARB) | Corrected calcium (CA) ***(Screening and 1-3 days Pre apheresis and immediately post apheresis)*** |
| Glucose (GLUC) | Phosphate (PHOS) |
| Urea | Lactate dehyrogenase (LDH) |
| Creatinine (CREAT) | Magnesium ***(Screening and 1-3 days Pre apheresis and immediately post apheresis )*** |
| Estimated glomerular filtration rate (eGFR) | Cholesterol ***(Screening only)*** |
| Albumin (ALB) | Triglycerides ***(Screening only)*** |
| Globulin | HDL ***(Screening only)*** |
| Total protein | Urate |
| Total bilirubin (BILI) |  |
| Direct bilirubin (BILDIR) |  |

Urinalysis

Urine will be tested by dipstick at the clinical unit. If there are any abnormalities considered clinically significant in blood, leucocytes or protein, the urine will be sent for formal laboratory urinalysis per the clinical unit standard procedure.

| Glucose (GLUC) |
| --- |
| Bilirubin (BILI) |
| Ketone (KETONES) |
| Specific gravity (SPGRAV) |
| Blood |
| pH |
| Protein (PROT) |
| Urobilinogen (UROBIL) |
| Nitrite |
| Leukocytes (WBC) |
| Formal laboratory analysis (if required) |

Urine drug screens and alcohol breath tests

If the results of the urine drug screens or alcohol breath tests are positive, subjects may be allowed to continue, or may be delayed or withdrawn according to site-specific instructions.

All subjects will be questioned about concomitant medications and use of recreational drugs. The urine drug screen may be repeated if the potential subject denies usage of any of these agents and the test result is believed to be a false positive.

Subjects testing positive for paracetamol at screening and/or inoculation day may still be eligible for study participation, at the Investigator’s discretion. Subjects requiring paracetamol on a daily basis would not be eligible to enrol in the study, as the use of any over-the-counter medication during the study is restricted and potential subjects should not discontinue their usual medications in order to participate in the study.

| **Urine Testing:** | |
| --- | --- |
| Amphetamines | Opiates |
| Methamphetamines | Phencyclidine |
| Barbiturates | Tetrahydrocannabinol (cannabis) |
| Benzodiazepines | Tricyclic antidepressants |
| Cocaine | Acetaminophen (paracetamol) |
| Methadone |  |
| **Alcohol breath test** | |

Serology

| HIV-1/HIV-2 antigen/antibody |
| --- |
| Hepatitis B (HBsAg, anti-HBc (IgG + IgM if IgG is positive)) |
| Hepatitis C (anti-HCV) |
| Syphilis EIA |
| Hepatitis A (anti-HAV) (IgM) - performed off stored sample for testing, at Investigator’s discretion |
| Hepatitis E (anti-HEV) (IgM) - performed off stored sample for testing, at Investigator’s discretion |
| EBV - performed off stored sample for testing, at Investigator’s discretion |
| CMV - performed off stored sample for testing, at Investigator’s discretion |

Assessment of blood for HMP bank ***(at screening or 1-3 days pre inoculation and again at Day 28 or Day 90; all timepoints at discretion of Investigator)***

The HMP bank questionnaire may be presented to the subject at the Investigator’s discretion at screening or the Day -3 to Day -1 visit, in order to assess eligibility to act as a donor for creation of a HMP bank (Appendix 6). If the subject is deemed suitable as a donor, additional blood (55 mL, Appendix 7) may be collected at screening or Day -3 to Day -1 and again at either Day 28±7 or Day 90±7 at the Investigator’s discretion for the tests listed below and if the whole blood volume does not exceed the maximum.

| Barmah Forest Virus IgG | Flavivirus IgM |
| --- | --- |
| Barmah Forest Virus IgM | Dengue PCR |
| River Ross Virus IgG | EBV PCR |
| River Ross Virus IgM | CMV PCR |
| Human T cell Lymphotropic Virus 1/2 | Parvo B19 PCR |
| Human Herpes Virus 6 | Human Herpes Virus 7 |

If the flavivirus IgM test is positive, tests may be performed at the Investigator’s discretion for confirmation and identification of the specific flavivirus. This may include tests for Japanese encephalitis virus, West Nile virus, Dengue virus (may include typing of 4 Dengue serotypes), and Murray Valley encephalitis virus.

Other laboratory tests

- RBC alloantibodies (Screening and Day 28±3 or Day 90±7 or early termination visit only).
- Glucose-6-phosphate dehydrogenase (G6PD) (Screening only).

Safety serum storage

Blood for serum storage as safety retention samples will be collected at Day 0 and at the final study visit.

### 7.2.2 Other Assays or Procedures

Malaria monitoring

Blood will be collected to monitor malaria paraite numbers using qPCR targeting the 18S rRNA gene. Additional blood (up to approximately 2 mL per time-point) may be collected for parasite lifecycle stage qRT-PCR at the Investigator’s discretion. This is to evaluate for the presence of sexual parasite stages (gametocytes) and other parasite lifecycle stages in the blood. This blood may also be used for research into various aspects of parasite biology e.g. gametocytes, parasite lifecycle stages, recrudescence, commitment etc. The qRT-PCR may target genes including but not limited to: the female gametocyte-specific transcript *pvs25*, the male gametocyte-specific transcript *pfMGET*, and the ring-stage transcript *pfSBP-1* as appropriate. This testing will occur between inoculation and day 28.

Microscopic examination for evidence of parasitaemia or gametocytaemia may be conducted at the Investigator’s discretion. Thick films may be prepared from blood collected at time-points coinciding with MFA.

Malaria transmission and mosquito infectivity assays

Transmission of gametocytes to *Anopheles* mosquitoes may be determined using direct skin feeding assays (DFA) and/or membrane feeding assays (MFA). Mosquitoes will be maintained in a controlled environment in the PC3 QIMR Berghofer insectary.

**DFA:** Subjects will be escorted to the insectary and asked to allow mosquitoes to feed on them at up to 2 time-points. Approximately 30 mosquitoes will be distributed into containers with gauze lids and starved prior to feeding assays. Mosquitoes will be allowed to bite on the volar surface of the forearms, thighs or calves of subjects to directly feed for approximately 15±5 minutes until fully engorged.

**MFA:** Up to approximately 80 mL blood (total) may be collected if maximum whole blood volume is not exeeded over either 1 or 2 time-points and transported at ~37°C. Mosquitoes (approximately 100) will be distributed into containers with gauze lids and starved prior to feeding assays. Mosquitoes will be allowed to feed for approximately 30 minutes on the blood through bovine caecum or parafilm membranes on water jacketed glass feeders attached to a 37°C water bath.

For both the DFA and MFA, after feeding, the number of non-engorged mosquitoes will be recorded. Following the feeds, the mosquitoes will be maintained in a controlled environment and will be provided with a sugar solution supplemented with 0.05% para-amino benzoic acid to promote the sporogonic cycle. Seven to ten days after blood feeding, mosquitoes will be dissected to quantify oocysts in midgut preparations using either malaria 18S qPCR, microscopy (oocysts will be stained with 0.1% mercurochrome) or CS ELISA.

A subset of fed mosquitoes may be kept for up to 24 days in order to investigate for development of salivary gland sporozoites. The prevalence and/or intensity of oocyst infection will be determined in the blood fed mosquitoes. The number of mosquitoes dying prior to dissection will be recorded.

Detection and evaluation of anti-blood-stage or anti-gametocyte specific antibodies

Plasma from blood samples taken for other purposes, e.g. membrane feeding assays, immunology samples etc., may be used at the Investigator’s discretion for the detection and evaluation of anti-blood-stage or anti-gametocyte specific antibodies. The plasma may be used for immunological assays including, but not limited to, enzyme-linked immunosorbent assays and indirect immunofluorescence assays.

Plasma samples and peripheral blood mononuclear cells for research on the immunology and pathophysiology of *P.vivax*

Plasma and PBMCs harvested using apheresis may be collected and stored for research on the immunology and pathophysiology of *P.vivax*, at the Investigator’s discretion. A baseline sample (20 mL) for plasma and PBMCs may also be collected on Day 0 pre-inoculation, at the Investigator’s discretion if the maximum whole blood volume is not exeeded.

### 7.2.3 Specimen Preparation, Handling, and Storage

Biological samples will be retained for the time required for assessment for analysis, and may then be discarded. Safety serum samples will be stored indefinitely with the permission of the subjects for any retrospective safety assessments.

### 7.2.4 Specimen Shipment

Samples collected will be shipped to nominated local or international laboratories for assessment. The site staff will be responsible for shipment of samples to analytical laboratories for testing. Samples must be packed securely together with completed shipment forms in shipping containers together with sufficient dry ice as per Shipper procedures.

## 7.3 Study Schedule

### 7.3.1 Screening

A screening visit will be scheduled after an initial telephone interview conducted by clinical unit staff has occurred to review background information. For the screening visit, potential subjects will be told to come to the clinical unit after an overnight fast of ≥8 hours. During this initial screening visit, the potential subject will read the Participant Information Sheet and be encouraged to ask questions. Individuals willing to be considered for inclusion may sign the screening consent form during the screening visit, or return to the clinical unit after further consideration. The subject will be given a copy of the Participant Information Sheet and signed consent form for their records. The signed and dated originals will be held on file by the CRU. Participation consent must be obtained from all eligible subjects prior to screening tests.

After providing written consent to participate, the subject will be examined by a medical officer and physical examinations, vital signs and ECG testing will be done together with collection of blood and urine samples for safety assessment. The subjects will be fully informed of the nature of the study at this time, and advised of the requirement to repeat some screening tests during the Day -3 to Day -1 safety visit (if required) and/or on the day of malaria challenge inoculum administration to determine their continuing eligibility. Subjects must confirm that they will not be living alone from Day 0 until completion of antimalarial treatment.

The pre-study screening will be conducted within 4 weeks prior to the Day 0 malaria challenge day and will include:

1. Provide the Main Participation Information Sheet and Informed Consent form and apheresis specific Participation Information Sheet and Informed Consent form and give the subject sufficient time to review the contents.
2. Explain the study via the main Participation Information Sheet and gain Informed Consent from the subject.
3. Ensure the subject has signed the main Participant Information Sheet and Informed Consent and received a signed copy.
4. The subject will then be escorted to the apheresis unit in the Cancer Care Services department at RBWH by a Q-pharm staff. A specialist in apheresis will explain the apheresis component of the study via the apheresis specific Participation Information Sheet and gain Informed Consent from the subject. The subject will also be provided with a leaflet explaining the apheresis procdure (lymphocyte collection leaflet).
5. A specialist in apheresis will ensure the subject has signed the apheresis specific Participant Information Sheet and Informed Consent and received a signed copy.
6. The subject will then be escorted back to the apheresis unit at the RBWH by Q-pharm staff.
7. A screening number will be assigned to each subject.
8. Elicit a complete medical history and use of medications.
9. Elicit a social history including alcohol and tobacco use.
10. Undertake a full physical examination.
11. Ask subject to complete the Beck Depression Inventory.
12. Ask subject to complete the HMP bank questionnaire and collect blood samples for HMP bank assessment (**at Investigator’s discretion** if the maximum whole blood volume is not exeeded) (Section 7.2.1).
13. Assessment of the cardiovascular disease risk (defined as >10%, 5 year risk when greater than 35 years of age) as determined by the Australian Absolute Cardiovascular Disease Risk Calculator ([*http://www.cvdcheck.org.au/*](http://www.cvdcheck.org.au/)). Risk factors include sex, age, systolic blood pressure (mm/Hg), smoking status, total and HDL cholesterol (mmol/L), and reported diabetes status.
14. Perform alcohol breath test.
15. Record vital signs.
16. Obtain a single 12-lead ECG.
17. Collect urine for urinalysis and urine drug screen.
18. Collect blood samples for haematology, biochemistry, RBC alloantibodies, G6PD testing, Duffy antigen, and serology (syphilis, viral hepatitis B and C and HIV).

**Blood samples must not be taken from veins located in the ante-cubital fossa.**

1. Verify subject meets inclusion/exclusion criteria.

Subjects who complete all screening procedures and satisfy all entry criteria will be considered eligible to participate in this study. To be eligible for study entry, laboratory values at screening must not be outside the range of the normal values at a level deemed to be clinically significant. For eligibility parameters a repeat may be requested to exclude laboratory error.

If screening laboratory results are abnormal, e.g. HIV testing, the volunteer will be referred for appropriate counselling. If any clinically significant abnormalities are detected during screening, the subject will be referred for follow-up tests to a general practitioner or medical specialist as appropriate.

### 7.3.2 Enrollment/Baseline

**Day -3 to Day -1 eligibility confirmation visit**

Subjects (including reserve subjects) will report to the CRU between Day -3 to Day -1 for the following baseline assessments, unless screening laboratory assessments were conducted within this period, in which case repeat sampling will not be required.

1. Ask subject to complete the HMP bank questionnaire and collect blood samples for HMP bank assessment (**at Investigator’s discretion** if the maximum whole blood volume is not exeeded).
2. Collect blood samples for haematology and biochemistry analysis.

**Blood samples must not be taken from veins located in the ante-cubital fossa.**

1. Collect urine for urinalysis.

The timing of these assessments is to ensure that results are available for review by the Investigator prior to inoculation on Day 0. Subjects with clinically significant laboratory findings at this stage will not be eligible for malaria parasite inoculation.

**Administration of Malaria Challenge Inoculum (Day 0)**

Each subject (and up to 3 reserve subjects) will report to the CRU on the morning of Day 0. The Investigator will review the subjects’ screening results prior to their enrolment into the study. The Investigator will emphasise the requirement to return for malaria drug treatment after the malaria inoculation. Subjects will be reviewed by the Investigator to confirm their continued eligibility for the study, including confirmation that they will not be living alone from Day 0 until the end of antimalarial treatment by checking housemates contact details recorded at screening visit.

On admission to the study centre, subjects will be required to undertake further screening procedures to determine whether they remain eligible to be enrolled. Subjects may have food until at least half an hour prior to inoculation. A reserve subject may be asked to replace a subject who does not continue to meet eligibility. These reserves will be compensated for the study visit even if not inoculated, as described in the Participant Information Sheet and Consent Form.

The procedures that will be undertaken prior to inoculation include:

1. Verify that all applicable eligibility criteria have been met.
2. Elicit information regarding any new medical conditions or illnesses since screening.
3. Perform alcohol breath test and urine drug screen.
4. Conduct symptom-directed physical examination.
5. Record vital signs.
6. Obtain a single 12-lead ECG.
7. Cannulate subjects with an indwelling intravenous cannula for the malaria inoculum, and record which arm is utilised.

**Cannulation must not be performed in veins located in the ante-cubital fossa.**

1. Collect blood samples for malaria 18S qPCR (parasitaemia baseline sample), parasite lifecycle stage qRT-PCR (parasite lifecycle stage baseline sample), immunology/ pathophysiology (baseline sample), and safety serum storage.

**Blood samples must not be taken from veins located in the ante-cubital fossa.**

Administration of the malaria inoculum:

1. Administer the malaria inoculum of ~1100 viable *P. vivax* infected human RBCs intravenously.
2. Observe for a minimum of 60 minutes after administration of the inoculum to evaluate for immediate adverse reactions.
3. Educate subjects on signs and symptoms of malaria (Appendix 2).
4. Emphasise to subjects the importance of returning on the nominated day for antimalarial treatment.
5. Provide subjects with diary cards and thermometers to record any temperature readings during the study in the event of symptoms of fever. Subjects will also record symptoms and concomitant medications on the diary cards during the study.
6. Record adverse events and concomitant medications.
7. Record vital signs prior to leaving the clinic (approximately 60 minutes after inoculation).
8. Record malaria clinical score prior to leaving the clinical unit (malaria clinical score baseline sample; see Section 7.1.1).

### 7.3.3 Follow-up

**Malaria monitoring via phone (Day 1 to Day 3)**

During this period, subjects are expected to be asymptomatic. A daily phone call or text message will be made to the subjects by clinic staff to monitor subject well-being and to solicit any adverse events.

**Daily visits for malaria monitoring (Day 4 until treatment day)**

Follow-up from Day 4 until the day of apheresis and rescue treatment initiation will be undertaken through daily visits (approximately 8:00 AM) to the clinical site. It may be required to increase the frequency of the visits to 3 times a day from day 9 until treatment to allow collection of blood samples for parasitaemia determination. The study investigator reserves the right to confine subjects earlier than planned for symptoms relief and observation prior to apheresis and treatment.

The following procedures will occur during these visits:

1. Perform symptom-directed physical examination when signs and symptoms of malaria are identified and it is clinically indicated at the Investigator’s discretion.
2. Record vital signs.
3. Collect blood samples for malaria 18S qPCR and parasite lifecycle stage qRT-PCR (if required). Sample may be collected up to 3 times per day from Day 9 until treatment day (at the discretion of the investigator and if maximum whole blood volume is not exeeded).

**Blood samples must not be taken from veins located in the ante-cubital fossa.**

1. Record malaria clinical score.
2. Record AEs and use of concomitant medications.
3. Advise subject that ibuprofen cannot be taken within 48 hours of apheresis.
4. Haematology and biochemistry (including calcium and magnesium) will be performed on Day 9.

**Study days for malaria transmission assays**

The transmission of parasites to mosquitoes may be measured using DFAs and/or MFAs at up to 2 time-points prior to apheresis and rescue treatment. Parasite lifecycle stage qRT-PCR will be used at the Investigator’s discretion to determine the timing of DFAs and/or MFAs. At these transmission assay time-points, vital signs will be recorded and a symptom-directed physical examination will be performed if symptoms or signs of malaria are identified and it is clinically indicated. Blood may also be collected for malaria 18S qPCR and parasite lifecycle stage qRT-PCR. Thick films may also be performed at the Investigator’s discretion.

**In-patient observation, apheresis and antimalarial treatment phase (confinement)**

Subjects will be admitted to the clinical unit for 72 hours (or less - 48h minimum - at the discretion of the investigator if the subject is deemed clinically well and the subject is happy for early release) when parasitaemia is >20,000 parasites/mL or the Malaria Clinical Score is >6 (within 24 hours of notification) or at the Investigator’s discretion.

Admission

The following procedures will occur at admission to the clinical unit (or just prior to the apheresis procedure for the blood sample collection):

1. Perform symptom-directed physical examination.
2. Record vital signs.
3. Perform alcohol breath test.
4. Collect urine for urinalysis and drug screen.
5. Collect blood samples for haematology, biochemistry, malaria 18S qPCR (pre-apheresis), and parasite lifecycle stage qRT-PCR. In the case where apheresis is not performed soon after admission but later in the day, blood samples collection will be postponed and performed just prior to the apheresis procedure.

**Blood samples must not be taken from veins located in the ante-cubital fossa.**

1. Record Malaria Clinical Score.
2. Record AEs and use of concomitant medications.

Apheresis, rescue treatment and observation

The following procedures will occur during the apheresis, rescue treatment and observation period:

1. The subject will be escorted to the apheresis unit in the Cancer Care Services department at RBWH by a staff member. The subject will then undergo the apheresis procedure as per the (SOP) (see Appendix 3) whilst being supervised by the apheresis specialist nurse. Time on the apheresis machine will be approximately 2-4 hours. The subject may have to remain in the apheresis unit slightly longer to allow connection to the apheresis apparatus. Once the procedure is over the subject will be escorted back to the clinical site (Q-Pharm). A Q-Pharm study doctor will be available by phone for the duration of the apheresis and be able to attend the subject within 10 minutes if needed. If the subject develops signs or symptoms of low calcium levels the apheresis specialist may advise the subject verbally to commence a high calcium diet.
2. Cannulate subjects with an indwelling intravenous cannula.
3. Collect blood samples for haematology, biochemistry (including calcium and magnesium) and malaria 18S qPCR (post-apheresis). A blood sample can also be collected for parasite lifecycle stage qRT-PCR (at the discretion of the investigator and if the maximum whole blood volume is not exceeded).
4. Obtain a single 12-lead ECG.
5. Administer artemether/lumefantrine treatment under direct observation.
6. Follow-up subjects as in-patients for 72 hours (or shorter - 48h mimimum - at the discretion of the investigator if the subject is deemed clinically well and the subject is happy for early release) to ensure tolerance of the therapy and adequate clinical response.
7. Perform symptom-directed physical examination when signs or symptoms of malaria are identified and it is clinically indicated at the Invesigator’s discretion.
8. Record vital signs 3 times a day whilst confined.
9. Record Malaria Clinical Score 3 times a day whilst confined.
10. Collect blood samples for malaria 18S qPCR at 12, , 24, 36, and 48 (if the subject remains in confinement for 72 h), hours following artemether/lumefantrine treatment initiation. Additional blood samples can be collected at the discretion of the investigator if required to ensure the safety of the subject.
11. Record AEs and use of concomitant medications.

Prior to exit from clinical unit

Subjects will be allowed to leave the unit 48 to 72 hours after initiation of artemether/lumefantrine treatment at the Investigator’s discretion if they are asymptomatic and have a normal examination and no clinically significant laboratory abnormalities. Subjects may be requested to stay in confinement longer than 72 hours at the Investigator’s discretion if deamed in their clinical interest.

The following procedures will occur prior to discharge from the clinical unit:

1. Perform symptom-directed physical examination.
2. Record vital signs.
3. Obtain a single 12-lead ECG.
4. Collect blood samples for haematology, biochemistry, malaria 18S qPCR. A blood sample can also be collected for parasite lifecycle stage qRT-PCR (at the discretion of the investigator and if the maximum whole blood volume is not exceeded).
5. Record AEs and use of concomitant medications.

**Chloroquine phosphate rescue treatment (if required)**

It is hypothesised that artemether/lumefantrine treatment will be curative considering that it is a registered drug used for treatment of *P. vivax* malaria infection. However, there is a possibility that resistance to artemether/lumefantrine by the *P. vivax* isolate HMPBS02-*Pv* may occur. In this case subjects will receive a standard course of therapy with chloroquine phosphate within 72 hours of administration of the first dose of artemether/lumefantrine treatment, at the discretion of the Investigator. Full details on the decision process to commence chloroquine treatment are presented in Section 6.1.5. If Chloroquine dosing is required, safety bloods (haematology and biochemistry) will be collected and assessed if not already scheduled prior to first dose and after last dose (or within 1 day of last dose). Monitoring of subjects post-chloroquine treatment will occur as presented above for artemether/lumefantrine treatment.

**Artesunate rescue treatment (if required)**

Treatment of subjects with i.v. artesunate will only occur in the event that subjects are unable to complete oral treatment with either artemether/lumefantrine or chloroquine (e.g. the subject is vomiting). Details on artesunate dosing are presented in Section 6.1.5. Subjects will be administered i.v artesunate on site and monitored as described above.

**Out-patient monitoring post-artemether/lumefantrine treatment (post confinement until safety visit)**

If the subject is released from confinement before 72h post Riamet^®^ treatment, a follow-up visit will be undertaken at 72h for clinical evaluation and blood sampling. If the 18S PCR result is positive, the subject will be followed up until a minimum of one negative qPCR is detected.

The following procedures will take place during these visits:

1. Collect blood samples for malaria 18S qPCR monitoring. Blood sampling for malaria monitoring will occur at the investigator‘s discretion if there is concern regarding the possible recrudescence of parasitaemia.
2. Collect blood samples for parasite lifecycle stage qRT-PCR at the Investigator’s discretion.
3. Collect blood samples for haematology and biochemistry, and urine for urinalysis at the Investigator’s discretion.
4. Perform symptom-directed physical examination when signs and symptoms of malaria are identified and it is clinically indicated at the Investigator’s discretion.
5. Record vital signs.
6. Record Malaria Clinical Score if vital signs are abnormal or at the Investigator’s discretion.
7. Record AEs and use of concomitant medications.

**Day 28±3**

The following procedures will occur on Day 28±3:

1. Perform full physical examination.
2. Record vital signs.
3. Obtain a single 12-lead ECG.
4. Collect urine for urinalysis.
5. Collect blood samples for haematology, biochemistry, malaria 18S qPCR and parasite lifecycle stage qRT-PCR (if required), RBC alloantibodies, and serology (at the discretion of the investigator and if required for safety reasons).
6. Collect blood samples for HMP bank assessment (**at Investigator’s discretion** and if the maximum whole blood volume is not exceeded).
7. Record AEs and use of concomitant medications.

**Follow-up phone call (Day 56±7)**

A phone call will be made on Day 56±7 to the participants by clinic staff to monitor participant well-being and to solicit any adverse events.

### 7.3.4 Final Study Visit

The following procedures will occur at the EOS visit (Day 90±7):

1. Perform symptom-directed physical examination.
2. Record vital signs.
3. Collect blood samples for haematology, biochemistry, malaria 18S qPCR and parasite lifecycle stage qRT-PCR (if required), RBC alloantibodies, and serology (if not performed on day 28 ±3).
4. Collect blood samples for safety serum storage.
5. Collect blood samples for HMP bank assessment (**at Investigator’s discretion** and if the maximum whole blood volume is not exceeded).
6. Record adverse events and use of concomitant medications.

### 7.3.5 Early Termination Visit

If withdrawal occurs at any stage of the study, the subject will be asked to complete an EOS evaluation. **In addition, subjects are informed on the essential requirement to complete the antimalarial drug treatment for their safety, via the Participant Information Sheet.**

Participation in an EOS evaluation by each subject is voluntary. Procedures during the early termination visit will include the following if withdrawal occurs prior to Day 28 visit:

1. Perform full physical examination.
2. Record vital signs.
3. Obtain a 12-lead ECG.
4. Collect urine sample for urinalysis.
5. Obtain blood for haematology, biochemistry, malaria qPCR and parasite lifecycle stage qRT-PCR, RBC alloantibodies and safety serum storage.
6. Record AEs and use of concomitant medications.

If withdrawal occurs after the Day 28 visit, the procedures outlined for the final study visit will be performed.

### 7.3.6 Unscheduled Visit

Unscheduled visits for malaria 18S qPCR or safety monitoring may be required at the Investigator’s discretion based on parasitaemia, clinical symptoms or laboratory results. Subjects will be contacted by phone to arrange these visits. Where possible, visits will be arranged at a time that is both convenient for the subject and meets any clinical urgency as determined by the Investigator. Unscheduled visits will be documented in the source documents and CRF.

### 7.3.7 Schedule of Events Table

The Schedule of Events Table summarises the procedures to be conducted as per this protocol during screening, confinement and post-confinement. Section 7.1 and 7.2 provide detailed information on the procedures.

Table 1 Schedule of Events

| Procedure | Screening | Eligibility confirmation^a^ | Malaria inoculation | Phone contact^p^ | Malaria monitoring^b^ | Apheresis and rescue treatment | Out-patient monitoring^t^ | Safety  visit | EOS visit |
| --- | --- | --- | --- | --- | --- | --- | --- | --- | --- |
|  | D-28 to D-1 | D-3 to D-1 | D0 | D1 to D3 | D4 to apheresis | Apheresis to end of confinement^c^ | Post confinement to Safey visit | D28±3 | D90±7 |
| Eligibility and safety assessments | | | | |  | | | | |
| Informed consent | X |  |  |  |  |  |  |  |  |
| Beck Depression Inventory | X |  |  |  |  |  |  |  |  |
| Medical history, eligibility & prior medications | X |  | X |  |  |  |  |  |  |
| Drug & alcohol screen | X |  | X |  |  | X^d^ |  |  |  |
| Full physical examination | X |  |  |  |  |  |  | X |  |
| Symptom-directed physical examination^e^ |  |  | X |  | X | X | X |  | X |
| Vital signs assessment | X |  | X |  | X | X | X | X | X |
| ECG | X |  | X |  |  | X^f^ |  | X |  |
| Urinalysis | X | X |  |  |  | X^d^ | X | X |  |
| Haematology & biochemistry | X | X |  |  | X^o^ | X^f^ | X | X^u^ | X^v^ |
| G6PD test & Duffy antigen | X |  |  |  |  |  |  |  |  |
| Assessment for HMP bank^i^ | X^q^ | X^q^ |  |  |  |  |  | X^r^ | X^r^ |
| Serology & RBC alloantibody | X |  |  |  |  |  |  | X^u^ | X^v^ |
| Safety serum storage |  |  | X |  |  |  |  |  | X |
| AEs & concomitant medications |  |  | X | X | X | X | X | X | X |
| Malaria clinical score |  |  | X |  | X | X | X^h^ |  |  |
| Malaria monitoring and apheresis | | | | | |  | | | |
| Malaria 18S qPCR blood sampling |  |  | X |  | X^t^ | X | X | X^u^ | X^v^ |
| Parasite lifecycle stage qRT-PCR blood sampling^i^ |  |  | X |  | X | X | X | X^u^ |  |
| Malaria transmission assays (DFA and/or MFA) |  |  |  |  | X^j^ | X^j^ |  |  |  |
| Apheresis |  |  |  |  |  | X^k^ |  |  |  |
| Immunology/pathophysiology |  |  | X^s^ |  |  | X^s^ |  |  |  |
| Rescue drug treatment | | | | | | | | |  |
| Riamet^®^ treatment |  |  |  |  |  | X^k^ |  |  |  |
| Chloroquine treatment |  |  |  |  |  | X^m^ |  |  |  |
| Artesunate treatment |  |  |  |  |  | X^n^ |  |  |  |

ECG: Electrocardiogram; G6PD: Glucose 6-phosphate dehydrogenase; RBC: Red blood cell; AEs: Adverse events; qPCR: quantitative polymerase chain reaction; qRT-PCR: quantitative reverse-transcriptase polymerase chain reaction; HMP: human malaria parasite; DFA: direct feed assay; MFA: membrane feed assay; EOS: End of study.

^a^ This visit is not required in the event that the sceening visit is conducted within this period.

^b^ Daily visits until Day 9. Up to 3 visits per day may be required, at the discretion of the investigator, between day 9 and treatment day.

^c^ Confinement is expected to start on day 9, 10, 11 or 12 and last between 48 to 72h.

^d^ At time of admission to clinical unit only.

^e^ Physical examinations should be performed prior to inoculation, upon admission to the clinical unit for apheresis and rescue treatment, and at Day 90±14/EOS. At all other times, symptom-directed physical examinations will only be performed when signs or symptoms of malaria are identified and it is clinically indicated at the Investigator’s discretion. Before exit from confinement, nursing staff must confirm with the study doctor or investigator if any subject requires symptom-driven examination pre discharge.

^f^ On day of admission (just prior to the apheresis procedure) and exit from clinical unit only. Haematology and biochemistry (including calcium and magnesium) will be taken upon return to Q-pharm immediately post apheresis.

^g^ Blood sampling for malaria may be done up to 3 times a day from Day 9 to treatment day (at the discretion of the investigator)

^h^ Only if vital signs are abnormal, or at the Investigator’s discretion.

^i^ At the Investigator’s discretion, if in doubt contact Investigator.

^j^ Transmission assays may be performed at up to 2 time points prior to apheresis and rescue treatment, at the discretion of the Investigator.

^k^ Apheresis and initiation of rescue treatment with artemether/lumefantrine will be performed on the first day of admission to the clinical unit (expected to occur on Day 10, 11 or 12).

^m^ Chloroquine will only be administered to subjects in the case of failure of artemether/lumefantrine therapy.

^n^ Intravenous artesunate treatment will only occur in the event that a subject is unable to complete oral treatment with either artemether/lumefantrine or chloroquine (e.g. the subject is vomiting).

^o^ Haematology and biochemistry (including calcium and magnesium) will be performed on Day 9 or Day 10 (day before apheresis).

^p^ A phone call will also be made on Day 56±7 to to monitor participant well-being and to solicit any adverse events.

^q^ Assessment for HMP bank at screening or Day -3 to -1 eligibility confirmation visit will include a questionnaire to be completed by each subject.

^r^ Blood for HMP bank may be taken at the Investigator’s discretion on day 28 or day 90 but not both and if the maximum whole blood volume is not exceeded.

^s^ At the Investigator’s discretion and if the maximum whole blood volume is not exceeded. If in doubt contact Investigator.

^t^ If subject is released from confinement before 72h post Riamet^®^ treatment.

^u^ At the discretion of the investigator and if required for safety reasons. Otherwise, blood sample will be collected on day 90±7 to limit the volume of vhole blood collected within 30 days of the apheresis procedure.

^v^ If not performed on day 28 ±3.

## 7.4 Justification for Sensitive Procedures

Not applicable.

## 7.5 Concomitant Medications, Treatments, and Procedures

Concomitant medications, treatments and procedures are those occurring from administration of the malaria inoculum until the end of the study (last visit). Those occurring prior to administration of the inoculum are classified as prior medications, treatments and procedures. Medications taken within 28 days before the malaria inoculation will be recorded as prior medication. Prior and concomitant medications, treatments and procedures permitted in this study are outlined in the inclusion/exclusion criteria (section 5.1 and 5.2).

On inoculation day, subjects will be questioned in relation to relevant aspects of compliance with the study protocol, including drug intake since their screening clinic visit. Details of all other drugs taken (prescription and over-the-counter, systemic and topical administration) will be recorded at this time and appropriate action taken. The Investigator may permit the use of ibuprofen up to 1.2 g/day (except during the 48 hour period prior to apheresis) or paracetamol up to 4 g/day, for treatment of headache or other pain if required. Any medication taken during the study for treatment of a medical condition or adverse event is to be recorded in the concomitant medication pages in the CRF (exact dose and timing of each dose to be specified).

### 7.5.1 Precautionary Medications, Treatments, and Procedures

Ibuprofen should not be taken in the 48 hour period prior to apheresis.

## 7.6 Prohibited Medications, Treatments, and Procedures

All concomitant medications other than those routinely used for symptom relief in IBSM trials (i.e. paracetamol, ibuprofen, ondansetron) or routine medications approved at screening (e.g. oral contraceptive) should be discussed with the Investigator before being approved unless deemed medically urgent. If the medication has already been taken it should be reviewed by the Investigator at the next opportunity and a decision should be made to continue, to stop, to switch to an alternative, or to withdraw the subject from the trial.

Subjects should not eat any poppy seeds in the 24 hours before the following time-points: screening, inoculation day, and day of admission for antimalarial treatment.

Subjects should not eat or drink any food or beverages that contain alcohol (e.g. beer, wine, and mixed drinks) from 24 hours prior to each alcohol breath test and from inoculation until the end of antimalarial treatment.

Subjects should not consume more than 400 mg caffeine per day, equivalent to more than 4 cups of coffee, from inoculation until the end of the antimalarial treatment.

## 7.7 Prophylactic Medications, Treatments, and Procedures

Not applicable.

## 7.8 Rescue Medications, Treatments, and Procedures

Rescue medications used in this study are defined in section 6 and details on their administration are given in section 7.3.

## 7.9 Subject Access to Study Agent At Study Closure

Not applicable.

# 8 ASSESSMENT OF SAFETY

## 8.1 Specification of Safety Parameters

Safety assessments to be performed in this study include recording of AEs, clinical laboratory measurements (haematology, biochemistry, urinalysis), physical examinations, vital signs recording, ECGs, and Malaria Clinical Score recording.

### 8.1.1 Definition of Adverse Events (AE)

An AE is any adverse change, i.e., any unfavourable and unintended sign (including an abnormal laboratory finding), symptom or disease that occurs in a subject during the course of the study, whether or not considered by the Investigator as related to study treatment.

AEs include:

- A new symptom, sign or medical condition.
- A disease or medical condition detected or diagnosed during the course of the study even though it may have been present prior to the start of the study.
- An exacerbation of a pre-existing medical condition or disease.
- An increase in frequency or intensity of a pre-existing episodic disease or medical condition.
- Continuous persistent disease or symptoms present at study start that worsen following the start of the study.
- An abnormal assessment (e.g. change on physical examination, ECG findings) if it represents a clinically significant finding that was not present at study start or worsened during the course of the study.
- An abnormal laboratory test result if it represents a clinically significant finding, symptomatic or not, which was not present at study start or worsened during the course of the study or led to dose reduction, interruption or permanent discontinuation of study treatment.
- Mosquito bite reactions (if DFA are performed) regardless of clinical significance.

Borderline abnormal laboratory findings and other objective assessments should NOT be routinely captured and reported as AEs, as they will be collected and analysed separately. However, abnormal laboratory findings or other objective measurements that meet the following criteria should be captured and reported in the AE section of the CRF:

- The result meets the criteria for reporting as an SAE
- The test result is associated with accompanying symptoms, and/or
- It requires additional diagnostic testing or medical/surgical intervention, and/or
- It leads to a change in trial dosing outside of protocol-stipulated dose adjustments, or discontinuation from the trial, significant additional concomitant drug treatment, or othertherapy, and/or
- It is considered by the Investigator or Sponsor to be clinically significant or represent a clinically significant change from baseline.

Merely repeating an abnormal test, in the absence of any of the above conditions, does not

constitute an AE. Any abnormal test result that is determined to be an error does not require

reporting as an AE.

If a clinical diagnosis is associated with an abnormal laboratory finding, the relevant adverse event should be recorded as the diagnosis rather than the incidental laboratory finding (e.g. “hepatitis” should be recorded rather than “elevated transaminases”).

Surgical procedures themselves are not AEs; they are therapeutic measures for conditions which

may, or may not, be AEs.

### 8.1.2 Definition of Serious Adverse Events (SAE)

A serious adverse event (SAE) is defined as an AE which fulfils at least one of the following criteria:

- Results in death
- Is life-threatening
- The term "life-threatening" in the definition of "serious" refers to an event in which the subject was at immediate risk of death at the time of the event; it does not refer to an event which hypothetically might have caused death if it was more severe.
- Requires inpatient hospitalisation or prolongs existing hospitalisation, unless this is for:
- Elective or pre-planned treatment or standard monitoring for a pre-existing condition that is unrelated to the study and has not worsened since the start of the study.
- Cosmetic surgery or for social reasons or respite care in the absence of any deterioration in the subject’s general condition.
- Results in persistent or significant disability/incapacity
- Is a congenital abnormality/birth defect
- Is considered medically important
- Medical and scientific judgement should be exercised in deciding whether other AEs are to be considered serious, such as important medical events that may not be immediately life-threatening but may jeopardise the subject or may require intervention to prevent one of the other outcomes listed in the definition above. Examples of such events are: intensive treatment in an emergency room or at home for allergic bronchospasm; blood dyscrasias; convulsions that do not result in hospitalisation; development of drug dependency or drug abuse.
- Constitutes a possible Hy’s Law case
- Hy’s Law case is defined as a subject with any value of alanine or aspartate aminotransferase greater than 3 × ULN together with an increase in total bilirubin to a value greater than 2 × ULN and not associated to an alkaline phosphatase value greater than 2 × ULN (FDA Guidance on Drug Induced Liver Injury: Premarketing Clinical Evaluation [2009]).

**A Suspected Unexpected Serious Adverse Reaction (SUSAR)** is any SAE where a causal relationship with the malaria challenge agent (*P. vivax* HMPBS02-*Pv*) or the antimalarial rescue drugs (artemether/lumefantrine, Chloroquine, or artesunate) is at least a reasonable possibility, but the event is not listed in the Investigator Brochure(s) and/or Summary of Product Characteristics.

### 8.1.3 Definition of Unanticipated Problems (UP)

Not applicable.

## 8.2 Classification of an Adverse Event

### 8.2.1 Severity of Event

In addition to determining whether an AE fulfils the criteria for a SAE or not, the severity of AEs experienced by study subjects will be graded according to the Common Terminology Criteria for Adverse Events v4.03 published 14 June 2010 (CTCAE v4.03). This guidance provides a common language to describe levels of severity, to analyse and interpret data, to scale the aggregate AE score, and to articulate the clinical significance of all AEs.

The severity of adverse events will be graded as follows:

- Grade 1: Mild; asymptomatic or mild symptoms; clinical or diagnostic observations only; intervention not indicated.
- Grade 2: Moderate; minimal, local or non-invasive intervention indicated; limiting age-appropriate instrumental activities of daily living.
- Grade 3: Severe or medically significant but not immediately life-threatening; hospitalisation or prolongation of hospitalisation indicated; disabling; limiting self-care activities of daily living.
- Grade 4: Life-threatening consequences; urgent intervention indicated.
- Grade 5: Death related to AE.

A mild, moderate, or severe AE may or may not be serious (see Section 8.1.2). These terms are used to describe the intensity of a specific event. Medical judgment should be used on a case-by-case basis.

Seriousness, rather than severity assessment, determines the regulatory reporting obligations.

### 8.2.2 Relationship to Study Agent

The Investigator will decide if AEs are related to any of the study agents or procedures. Where possible, a distinction should be made between events considered related to the malaria challenge agents or the apheresis procedure and those related to protocol-mandated procedure (including mosquito feeding, and rescue medication).

The assessment of causality will be made using the following definitions:

**Unrelated**

This category is applicable to those AEs which are judged to be clearly and incontrovertibly due to extraneous causes (disease, environment, etc.) and do not meet the criteria for the relationship listed under unlikely, possible or probable.

**Unlikely**

In general, this category is applicable to an AE which meets the following criteria (must have the first two):

1. It does not follow a reasonable temporal sequence from administration of any of the study agents.

2. It may readily have been produced by the subject’s clinical state, environment or toxic factors, or other modes of therapy administered to the subject.

3. It does not follow a known pattern of response to the study agents.

4. It does not reappear or worsen when any of the study agents are re-administered.

**Possible**

This category applies to those AEs in which the connection with any of the study agents appears unlikely but cannot be ruled out with certainty. An adverse event may be considered possible if or when (must have the first two):

1. It follows a reasonable temporal sequence from administration of any of the study agents.

2. It may have been produced by the subject’s clinical state, environment or toxic factors, or other modes of therapy administered to the subject.

3. It follows a known pattern of response to any of the study agents.

**Probable**

This category applies to those adverse events which are considered, with a high degree of certainty, to be related to the study agents. An adverse event may be considered probable if (must have the first three):

1. It follows a reasonable temporal sequence from administration of any of the study agents.

2. It cannot be reasonably explained by the known characteristics of the subject’s clinical state, environment or toxic factors, or other modes of therapy administered to the subject.

3. It disappears or decreases on cessation or reduction in dose.

4. It follows a known pattern of response to any of the study agents.

5. It reappears on re-administration.

### 8.2.3 Expectedness

An AE is regarded as an unexpected event if its nature or severity is not consistent with the applicable reference safety information (Investigator’s Brochures or approved manufacturer’s prescribing information for marketed drugs). Events that add significant information on the specificity, severity or frequency of previously described reactions, are also regarded as unexpected.

Expected AEs from the malaria infection are listed in Appendix 2 and the Investigator’s Brochure for the *P. vivax* HMPBS02-*Pv* challenge agent. Expected AEs from the antimalarial drugs used are listed in the artemether/lumefantrine and Chloroquine Consumer Medicine Information (see Appendix 4) and artesunate product insert.

## 8.3 Time Period and Frequency for Event Assessment and Follow-Up

All AEs must be documented and followed up by the Investigator until:

• the event is resolved, or

• no further medically relevant information in relation to the event can be expected, and

• the Investigator considers it justifiable to terminate the follow-up.

Events that are unresolved at the time of the subject’s last follow-up visit should continue to be

followed up by the Investigator for as long as medically indicated. The Sponsor retains the right to request additional information for any subject with ongoing AE(s)/SAE(s) at the end of the study, if judged necessary.

All AEs should be treated appropriately. The Investigator will decide upon the appropriate action to be taken in response to an AE, which may include one or more of the following:

• no action taken (i.e. further observation only)

• apheresis is withheld and the subject withdrawn from the study

• administration of a concomitant medication

• hospitalisation or prolongation of current hospitalisation (event to be reported as an SAE)

• other.

In a case of occurrence of SAEs, regardless of whether or not it is judged to be challenge agent- or antimalarial drug-related, the subject will receive appropriate care under clinical supervision until all the symptoms of the SAEs have diminished or resolved and the subject’s condition improved.

For ongoing AEs, care will be provided for a period of time as specified in the clinical site work instruction protocols. However, if the nature of the ongoing AE is determined by the Investigator as not being inoculum-, apheresis-, or antimalarial drug-associated, the subject will be advised to visit his/her own general practitioner for further clinical care that he might require.

## 8.4 Reporting Procedures

### 8.4.1 Adverse Event Reporting

It is the Investigator’s responsibility to document and report all AEs occurring in the clinical trial whether spontaneously reported by the subject, observed by the Investigator (either directly or by laboratory or other assessments), or elicited by general questioning. The period of observation for collection of AEs extends from the time of inoculation up to the end of the study. Events reported prior to this will be recorded as medical history, unless the symptoms worsen during the study.

The following information should be recorded for all AEs:

• a description of the AE

• the dates and times of onset and resolution of the event

• the duration of the event in hours

• the time of onset relative to the administration of the inoculum and/or apheresis

• the seriousness and severity of the event

• the action take in response to the event (including treatment required)

• the outcome of the event

• the relationship of the event to the study agents (causality assessment), including inoculum, apheresis, mosquito feeding, rescue medication, or any other treatment or procedure conducted during the study.

Changes in the severity of an AE will be documented to allow assessment of the duration of the event at each level of severity. AEs changing severity will be documented as separate AEs; those worsening in severity will be considered unresolved and those reducing in severity will be considered resolving. AEs characterised as intermittent require documentation of onset and duration at each episode.

All malaria-specific AEs will be tabulated and results graded according to a purpose-designed Malaria Clinical Score (Section 7.1.1).

### 8.4.2 Serious Adverse Event Reporting

The Investigator will take immediate appropriate action in response to SAEs to ensure subject safety and in an attempt to identify the causes of the event. Review and reporting of SAEs will be in accordance with the Sponsor’s and Q-Pharm’s SAE reporting procedures. The Investigator will notify the Sponsor representative (QIMR Berghofer Regulatory Affairs), the QIMR Berghofer-HREC, and the IMM of the occurrence of any SAE within 24 hous of becoming aware of the event. The notification should be in writing by email or fax, and documented on a standard SAE reporting form.

Sponsor Representative

QIMR Berghofer Regulatory Affairs

Email: clinical.trials@qimrberghofer.edu.au

QIMR Berghofer-HREC Contact

QIMR Berghofer HREC Secretary

E-mail: HREC.Secretariat@qimrberghofer.edu.au

Phone: +61 7 3362 0117

Independent Medical Monitor:

Professor Dennis Shanks

E-mail: Dennis.SHANKS@defence.gov.au

Phone: +61 7 3332 493

The Investigator will complete a follow-up SAE report within 14 days of the SAE, unless no further information is available in which case the follow-up report will be provided as soon as new information becomes available. The follow-up SAE report will be sent to QIMR Berghofer Regulatory Affairs, the QIMR Berghofer HREC, and the IMM. Other supporting documents may be requested by these parties and will be provided by the Investigator or a delegate as soon as possible.

Any SAE that meets the criteria of a SUSAR (Section 8.1.2) will be reported to the TGA by QIMR Berghofer Regulatory Affairs in accordance with the Sponsor’s reporting procedures.

### 8.4.3 Unanticipated Problem Reporting

Not applicable.

### 8.4.4 Events of Special Interest

Not applicable.

### 8.4.5 Reporting of Pregnancy

Not applicable.

## 8.5 Study Halting Rules

See Section 5.5.

## 8.6 Safety Oversight

Safety oversight will be undertaken by the Principal Investigator , the collaborating PI, and the IMM who will serve as an independent expert to advise on clinical safety specifically in the situation where expert external advice is required regarding the need for administration of alternative/rescue antimalarial treatment in the circumstance of suboptimal response.

The Safety Review Team (SRT) will be responsible for decisions related to the safety of subjects and the continuation of the study. The role and composition of the SRT is outlined in the study specific SRT Charter. The SRT will meet approximately 4 weeks after the inoculation of each subject to review safety data before proceeding with the inoculation of a subsequent subject. A final SRT will also take place at completion of the study which will review safety parameters for all the subjects involved. The SRT will be composed of the Principal Investigator, the collaborating PI, the IMM, and a physician with expertise in clinical trials or infectious diseases. The SRT will review the clinical and laboratory safety data as well as the recorded AEs and SAEs. The SRT makes recommendations to the Sponsor. These recommendations are approved by the SRT Chair who signs a letter of recommendation that is sent to the Principal Investigator and the Sponsor.

Additionally, the SRT may meet to assess any events that trigger the stopping rules (Section 5.5) or as needed to provide a recommendation and findings to QIMR Berghofer HREC and the Principal Investigator, in accordance with the approved SRT Charter.

Whether at a scheduled or unscheduled meeting, the SRT will consider safety signals to determine whether or not they can recommend that the study continue.

# 9 CLINICAL MONITORING

It will be the Sponsor’s responsibility to ensure that the study is monitored in accordance with the requirements of GCP. The conduct of the study will be reviewed internally by the clinical unit (Q-Pharm) in accordance with their standard procedures and work instructions, and GCP guidelines. The study will be monitored according to the Sponsor’s SOPs and all protocol deviations will be reported to the Sponsor (see Section 14.3 for more detail). Protocol deviations that impact subject safety or data integrity will also be reported to the QIMR Berghofer HREC.

During the study, appointed study monitor(s) (on behalf of the Sponsor) will visit the site to check completeness of subject records, accuracy of CRF entries, adherence to the protocol and to GCP, progress of enrolment, and to ensure that study agents were stored, dispensed, and accounted for according to specifications. Key study personnel are required to be available to assist the study monitor during these visits.

The Investigator will be required to give the monitor access to all relevant source documents to confirm their consistency with the CRF entries. The Sponsor will require full verification for the presence of informed consent, adherence to the inclusion/exclusion criteria, documentation of SAEs, and the recording of data that is used for all primary and safety variables. Additional checks of the consistency of the source data with the CRFs will be performed according to the study-specific monitoring plan. No information in source documents about the identity of the subjects will be disclosed.

# 10 STATISTICAL CONSIDERATIONS

## 10.1 Statistical and Analytical Plans

This study is a Phase I exploratory study to investigate the safety and plausability of apheresis as a method for harvesting parasites from healthy subjects experimentally infected with blood stage malaria. As such, no formal statistical analysis plan will be generated.

## 10.2 Statistical Hypotheses

Not applicable.

## 10.3 Analysis Datasets

The safety analysis dataset will include all subjects who receive the malaria inoculum. This population will be used to analyse all safety data as well as demographic and baseline data.

## 10.4 Description of Statistical Methods

### 10.4.1 General Approach

All measured variables and derived values will be listed. Continuous data will be summarised using descriptive statistics (mean and standard deviation, or median and interquartile range). Categorical data will be presented using N and % (using the number of subjects without missing data in the calculation).

### 10.4.2 Analysis of the Primary Efficacy Endpoint(s)

Not applicable.

### 10.4.3 Analysis of the Secondary Endpoint(s)

No formal statistical analysis of the secondary endpoints will be performed, see Section 10.4.1.

### 10.4.4 Safety Analyses

The overall number and percentage of subjects with at least one AE (and SAE) will be tabulated over the entire study period. All AE data will be summarised by MedDRA system organ class and preferred term, and maximum severity. Vital signs, routine safety laboratory data and ECG parameters will be summarised descriptively by time-point. Both absolute values and change from baseline (inoculation) will be presented.

### 10.4.5 Adherence and Retention Analyses

Not applicable.

### 10.4.6 Baseline Descriptive Statistics

Demographic data will be summarised by descriptive statistics and will include total number of observations (n), mean, standard deviation (SD) and range for continuous variables and number and percentages with characteristics for dichotomous variables.

The subject disposition will be summarised. Study completion, study withdrawals, exclusions and violations will be summarised and the reasons for withdrawal, exclusions and violations will be listed.

Medical history, current medical conditions, previous and concomitant medications, results of laboratory screening tests, drug tests and any other relevant baseline information will be listed by subject.

### 10.4.7 Planned Interim Analyses

#### 10.4.7.1 Safety Review

There will be an SRT meeting approximately 4 weeks after the inoculation of each subject to review safety data before proceeding with the inoculation of a subsequent subject. There will also be a final SRT after the last subject which will review any SAEs, AEs, blood tests, vital signs or other investigations of concern. The SRT members must agree on the safety of the trial before going forward with the inoculation of the subsequent subject.

#### 10.4.7.2 Efficacy Review

The SRT meeting undertaken approximately 4 weeks after the inoculation of the each subject will also assess the progress of the each subject in relation to the success or failure in extracting and concentrating all stages of malaria parasites by apheresis. The SRT may decide not to proceed with inoculating further subjects if they deem it futile and highly unlikely to be successful.

### 10.4.8 Additional Sub-Group Analyses

Not applicable.

### 10.4.9 Multiple Comparison/Multiplicity

Not applicable.

### 10.4.10 Tabulation of Individual Response Data

All individual subject data will be listed by measure and time point.

### 10.4.11 Exploratory Analyses

Not applicable.

## 10.5 Sample Size

This study is a exploratory study to investigate the safety and plausibility of apheresis as a method for extracting and concentrating parasites from healthy subjects experimentally infected with blood stage *P. vivax*. Therefore, statistical considerations regarding sample size do not apply. We estimate that a total of up to 8 subjects inoculated in a sequential manner will be sufficient to meet the objectives of this study. Approximately 4 weeks after the inoculation of each subject an assessment of all data collected will be performed. If it is deemed by the PI that there is no need to proceed further as all objectives of the study have been met, the study may be considered complete with fewer than 8 subjects enrolled.

## 10.6 Measures to Minimize Bias

### 10.6.1 Enrollment/ Randomization/ Masking Procedures

Treatment numbers will be allocated prior to inoculation and will serve as subject identifiers and for the purpose of distinction between cohorts, and will not correspond to a randomisation schedule.

Treatment numbers will be defined according to the CRU standard operating procedures (SOPs) and noted in a work instruction prior to study start. These numbers will be assigned to subjects on the morning of the dose administration after confirmation of their continued eligibility, in ascending, sequential order corresponding to the sequence of screening numbers for the subject in the admitted cohort. A log will be maintained at the site cross-referencing each subject’s screening number to the treatment number assigned.

After allocation, treatment numbers will be recorded in the CRFs and displayed behind each subject’s bed and on a wrist band at all times during the admission period.

### 10.6.2 Evaluation of Success of Blinding

Not applicable.

### 10.6.3 Breaking the Study Blind/Subject Code

Not applicable.

# 11 SOURCE DOCUMENTS AND ACCESS TO SOURCE DATA/DOCUMENTS

The Investigator will maintain source documents for each subject in the study. Information entered into CRFs will be traceable to these source documents in the subject's file. The Investigator must certify that the data entered into the CRFs are complete and accurate. Documentation of the apheresis procedure will occur as normal in the specialist centre (see Appendix 3) and will be retained as a source document in the subject’s file. After database lock, the Investigator will retain copies of the subject data for archiving at the investigational site.

Upon request, the Investigator(s)/institution(s) will permit direct access to source data/documents for trial-related monitoring, audits, Ethics Committee review, and regulatory inspection(s) by the Sponsor (or their appropriately qualified delegate) and Regulatory Authorities. Direct access includes examination, analysis, verification and reproduction of records and reports that are important to the evaluation of the trial.

# 12 QUALITY ASSURANCE AND QUALITY CONTROL

Data management will be performed in accordance with regulatory requirements. The data entered into the CRFs by investigational staff will be reviewed for completeness and accuracy. The site personnel will clarify any apparent erroneous entries or inconsistencies and additional information will be requested from the site as required.

Medical history/current medical conditions and adverse events will be coded using the Medical

dictionary for regulatory activities (MedDRA) terminology (version 19.0 or later).

After all data have been captured and reviewed, all queries have been resolved with the site and any protocol non-compliances that were identified during the data management processes have been confirmed by the site, the database will be declared to be complete and accurate, it will be locked and made available for data analysis. Any changes to the database after that time may only be made by the data manager, in consultation with the Sponsor and in accordance with documented database unlock and relock procedures.

Clinical monitoring will be conducted as described in Section 9.

Audits may be carried out by Sponsor quality assurance representatives, local authorities or

authorities to whom information on this study has been submitted. All documents pertinent to this study must be made available for such inspections after adequate notice of intention to audit.

# 13 ETHICS/PROTECTION OF HUMAN SUBJECTS

## 13.1 Ethical Standard

The study will be conducted in accordance with the protocol approved by QIMR Berghofer HREC and the Royal Brisbane and Women’s Hospital HREC, the principles of the Declaration of Helsinki (Recommendations guiding Medical Doctors in Biomedical Research Involving Human Subjects, Fortaleza, Brazil 2013), the NHMRC National Statement on Ethical Conduct in Human Research (2007) and the Notes for Guidance on Good Clinical Practice (GCP) (CPMP/ICH/135/95), as adopted by the Australian Therapeutic Goods Administration (2000).

The Investigator will minimise any discomfort experienced by subjects during the study. The only invasive procedures will be the intravenous inoculation of the malaria inoculum and the blood collection by cannulation/venipuncture and apheresis.

The maximum amount of blood to be collected from an individual in the study would be up to approximately 286 mL if HMP bank production is not performed (i.e. a volume which will not be more than the equivalent to a standard blood bank donation and it will be taken over at least a 4-8 week interval). See Appendix 1.

The total volume of blood drawn from each subject will not exceed 450 mL in any given 30 day period. This volume includes allowance for unscheduled safety and qPCR assessments that may be required at the discretion of the Principal Investigator or the Sponsor to ensure subject safety.

## 13.2 Ethical Review

The protocol, consent forms and participant information sheets will be reviewed by the QIMR

Berghofer-HREC and the Royal Brisbane and Women’s Hospital HREC. No study activities will be initiated prior to the approval of these documents by those committees. All amendments and addenda to the protocol will similarly be submitted to the QIMR Berghofer-HREC and the Royal Brisbane and Women’s Hospital HREC for approval prior to their implementation.

Changes to the final study protocol can only be made with the prior consent of the Principal Investigator, the Sponsor and the HREC. All such changes must be attached to, or incorporated into, the final protocol, and communicated to all relevant members of Q-Pharm staff and, if appropriate, to study subjects. All deviations from this study protocol will be included in the trial master file and included in the CSR. An assessment of the significance of each protocol deviation will be given in the CSR. All deviations/amendments will be reported to the Sponsor. The different types of amendments are discussed below.

Non-substantial amendment

Administrative or logistical minor changes require a non-substantial amendment. Such changes include but are not limited to changes in study staff or contact details (e.g., Sponsor instead of CRO monitors) or minor changes in the packaging or labelling of study drug. An amendment deemed to be non-substantial must have no ethical implications.

The implementation of a non-substantial amendment may be done without notification to the HREC. It does not require their approval or to be signed by the Investigator. The HREC will be notified for these non-substantial changes in the next submission round, with the annual study report or study close out report which ever comes sooner that will be submitted to HREC.

Substantial amendment

Significant changes require a substantial amendment. Significant changes include but are not limited to: new data affecting the safety of subjects, change of the objectives/endpoints of the study, eligibility criteria, dose regimen, study assessments/procedures, treatment or study duration, with or without the need to modify the Participant Information Sheet and Informed Consent.

Substantial amendments are to be approved by the HREC. The implementation of a substantial amendment can only occur after formal approval by the HREC and must be signed by the Investigator.

Urgent amendment

An urgent amendment might become necessary to preserve the safety of the subjects included in the study. The requirements for approval should in no way prevent any immediate action being taken by the Investigator or the Sponsor in the best interests of the subjects. Therefore, if deemed necessary, an Investigator can implement an immediate change to the protocol for safety reasons. This means that, exceptionally, the implementation of urgent amendments will occur before submission to and approval by the HREC.

In such cases, the Investigator must notify the Sponsor within 24 hours. A related substantial amendment will be written within 10 working days and submitted to the HREC, together with a description of the steps that have already been taken in regard to implementation of this amendment.

HREC approval of future research

In the event that the Principal Investigator or the Sponsor want to perform testing on the samples that is not described in the protocol, additional HREC approval will be sought. This may be done if a subject consents to blood storage for use in future research (Section 13.3.1).

## 13.3 Informed Consent Process

### 13.3.1 Consent/assent and Other Informational Documents Provided to Subjects

Subjects will be fully informed of the nature of the study, the properties and adverse effects of the inoculum, apheresis procedure, Chloroquine treatment and potential rescue treatment with artemether/lumefantrine and all relevant aspects of study procedures in the ‘Participant Information Sheet’.

The Participant Information Sheet and informed Consent Form describes in detail the study agents, study procedures, and risks. Subjects will also receive an Informed Consent for Blood Storage and an option to grant permission to be contacted about future studies involvement.

Subjects will also receive the Consumer Medicine Information for artemether/lumefantrine. Subjects may also receive the Consumer Medicine Information for chloroquine (Appendix 4), and the product insert for artesunate if required.

### 13.3.2 Consent Procedures and Documentation

During the initial screening visit/recruitment, potential subjects will read the Participant Information Sheet. The Investigator or clinical unit staff will explain the study via the Participant Information Sheet and the potential subjects will be encouraged to ask questions. Individuals willing to be considered for inclusion in the study will sign and date the informed Consent Form in the presence of an Investigator. Subjects will be given a copy of their signed informed Consent Form. Once the subject has consented to the study, the trial-specific screening activities may commence. See Section 7.3.1 for further details.

## 13.4 Subject and data Confidentiality

Subjects will be informed that their data will be held on file by Q-Pharm and that these data may be viewed by staff of Q-Pharm (including, where necessary, staff of Q-Pharm other than the named Investigators).

Upon request, the Investigator(s)/institution(s) will permit direct access to source data/documents for trial-related monitoring, audits, Ethics Committee review, and regulatory inspection(s) by the Sponsor (or their appropriately qualified delegates) and Regulatory Authorities (see Section 11).

Subjects will be informed that a report of the study will be submitted to the Sponsor and may also be submitted to government agencies and perhaps for publication, but that they will only be identified in such reports by their study identification number, and their gender and age. The Investigators undertake to hold all personal information in confidence.

Subjects will be informed that samples collected for the purposes described in the protocol will be sent to Sponsor’s nominated national or international laboratory for assessment.

### 13.4.1 Research Use of Stored Human Samples,Specimens or Data

Samples and data collected during this study will be used to achieve the study objectives. Samples and data will be stored according to Q-Pharm and QIMR Berghofer SOPs, and access will be limited to authorised personnel. Biological samples will be retained for the time required for assessment for analysis, and may then be discarded.

## 13.5 Future Use of Stored Specimens

As part of the study, safety serum samples will be stored indefinitely at Q-Pharm/QIMR Berghofer for retrospective safety assessments that may later be indicated. Subjects consent to this storage and the use of the sample for safety assessments, when they sign the informed Consent Form for the study.

For all other samples, consent must be obtained from the subjects to store and use their samples for future research. This is done via the Informed Consent for Blood Storage that subjects receive during recruitment/screening. Subjects can decide if they want their samples to be used for future research or have their samples destroyed at the EOS. A subject’s decision can be changed at any time prior to the EOS by notifying the study doctors or nurses in writing. However, if a subject consents to future use and some of their blood has already been used for research purposes, the information from that research may still be used.

Any future research using the stored samples that is beyond the current study will be reviewed by the QIMR Berghofer HREC (Section 13.2). All samples will be stored at QIMR Berghofer in accordance with the laboratory SOPs. The Investigator will ensure that confidentiality will be maintained continuously in all future research that involves use of these samples. The vials containing the samples of the consented subjects will be coded and the identifying information will not be released to any unauthorised third party. The subjects can also choose (via the Informed Consent for Blood Storage Form) for the samples to be re-labelled with only the study number, malaria strain and visit. No genetic testing will be performed on the stored samples without obtaining consent from the subjects. The stored samples will not be sold or used directly for production of any commercial product. There are no benefits to subjects in the collection, storage and subsequent research use of their samples. Reports about future research done with subject samples will NOT be kept in their health records, but a subject’s samples may be kept with the study records or in other secure areas.

# 14 DATA HANDLING AND RECORD KEEPING

## 14.1 Data Collection and Management Responsibilities

Each subject will have a clinical file (source data) and case report form (CRF, for protocol specific data) into which relevant data will be recorded. All recording will be done only in black ink. Corrections will only be made by drawing a single line through the incorrect entry, writing the correction in the nearest practicable space, and initialing and dating the correction. Correction fluids are not allowed.

A log of names, signatures and initials of all staff authorised to enter data into a subject’s Clinic File and CRF will be kept. Upon completion of each study visit, all CRFs will be reviewed internally by the clinic for omissions or apparent errors so that these can be corrected without delay. Any corrections made after the review and signature of the Principal Investigator will be noted in the audit trail and will required reauthorisation (electronic sign off) by the Principal Investigator.

## 14.2 Study Records Retention

All source data, clinical records and laboratory data relating to the study will be retained in the

archive of the clinical unit (Q-Pharm) for a minimum of 15 years after the completion of the study. Data will be available for retrospective review or audit by arrangement with the Chief Executive Officer of the clinical unit (Q-Pharm). Written agreement from the Sponsor must precede destruction of the same.

## 14.3 Protocol Deviations

A protocol deviation is any change, divergence or departure from the study design or procedures defined in the protocol.

All protocol deviations will be documented in the trial master file and included in the CSR. An assessment of the significance of each protocol deviation will be discussed in the CSR.

All protocol deviations will be reported to the Sponsor. Protocol deviations that are not approved by the Sponsor and QIMR Berghofer HREC prior to implementation should be reported to the Sponsor by the clinical unit. Protocol deviations should be recorded on a protocol deviation log by the clinical unit staff. Protocol deviation logs should be submitted to the Sponsor and QIMR Berghofer HREC via inclusion with the annual report.

Protocol deviations that impact subject safety or data integrity will be reported to both the Sponsor and the QIMR Berghofer HREC in a timely manner. A protocol deviation report form will be used for this purpose.

## 14.4 Publication and Data Sharing Policy

The data management, statistical and medical writing team appointed by the Sponsor will collaborate to provide a detailed CSR upon conclusion of the study. This will include appendices of all tables and listings generated during the analyses of data. The Sponsor undertakes to ensure that all safety observations made during the conduct of the trial are documented in this report.

Publication and reporting of results and outcomes of this trial will be accurate and honest, undertaken with integrity and transparency and in accordance with the relevant clauses outlined in the QIMR Berghofer Policy on Criteria for Authorship. QIMR Berghofer and the Principal Investigator have a responsibility to ensure that results of scientific interest arising from the clinical trials are appropriately published and disseminated. Publication of results will be subjected to fair peer-review. Authorship will be given to all persons providing significant input into the conception, design, and execution or reporting of the research according to the QIMR Berghofer Policy on Criteria of Authorship. No person who is an author, consistent with this definition, will be excluded as an author without his/her permission in writing. Authorship will be discussed between researchers prior to study commencement (or as soon as possible thereafter) and reviewed whenever there are changes in participation. Joint publications between QIMR Berghofer and the Royal Brisbane & Women’s Hospital are envisaged. Acknowledgment will be given to collaborating institutions and hospitals and other individuals and organisations providing finance or facilities. All conflicts arising through disputes about authorship will be reviewed by the QIMR Berghofer Director.

Data will not be released publicly until the manuscript is accepted for publication. In the case of no publication, information will only be released to the public and media in accordance with the QIMR Berghofer Corporate Media Strategy Policy. However, the Investigator undertakes not to make any publication or release pertaining to the study and/or results of the study without the Sponsor’s prior written consent, being understood that the Sponsor will not unreasonably withhold its approval. The Sponsor has the right to publish the results of the study at any time.

The Investigator shall not use the name(s) of the Sponsor and/or of its employees in advertising or promotional material or publication without the prior written consent of the Sponsor. The Sponsor shall not use the name(s) of the Investigator and/or the collaborators in advertising or promotional material or publication without having received his/her and/or their prior written consent(s).

QIMR Berghofer will ensure that the key design elements of this protocol are posted in a publicly accessible database such as Australian New Zealand Clinical Trials Registry (ANZCTR) or Clinicaltrials.gov. In addition, upon study completion and finalisation of the study report the results of this trial will be either submitted for publication in an open access journal and/or posted in a publicly accessible database of clinical trial results.

# 15 STUDY ADMINISTRATION

## 15.1 Study Leadership

See Section 1 for key roles.

## 15.2 Liability/Indemnity/Insurance

The study Sponsor will ensure sufficient insurance is available to enable it to indemnify and hold the investigator(s) and relevant staff as well as any hospital, institution, Ethics Committee or the like, harmless from any claims for damages for unexpected injuries, including death, that may be caused by the participant’s participation in the study but only to the extent that the claim is not caused by the fault or negligence of the subjects or investigator(s). The Sponsor adheres to the guidelines of Medicines Australia for injury resulting from participation in a company sponsored trial, including the provision of ‘No-fault clinical trial insurance’.

# 16 CONFLICT OF INTEREST POLICY

No conflicts of interest are applicable in this study.

# 17 LITERATURE REFERENCES

1. WHO. World Malaria Report 2014. 2014.
2. Sauerwein RW, Roestenberg M, Moorthy VS: Experimental human challenge infections can accelerate clinical malaria vaccine development. Nat Rev Immunol 2011, 11:57–64.
3. McCall MB, Netea MG, Hermsen CC, Jansen T, Jacobs L, Golenbock D, van der Ven AJ, Sauerwein RW: *Plasmodium falciparum* infection causes proinflammatory priming of human TLR responses. J Immunol 2007, 179:162–171.
4. Kamau E. Alemayehu S, Feghali KC *et al* Measurement of parasitological data by quantitative real-time PCR from controlled human malaria infection trials at the Walter Reed Army Institute of Research, Malaria Journal, 2014, 13: 288, 1-8.
5. Rockett R.J., Tozer S.J., Peatey C., Bialasiewicz S., Whiley D.M., Nissen M.D., Trenholme K., McCarthy J.S., Sloots T.P. A real-time, quantitative PCR method using hydrolysis probes for the monitoring of *Plasmodium falciparum* load in experimentally infected human subjects. Malaria Journal 2011, 10:48.
6. Ward D. Conventional apheresis therapies: a review. Journal of clinical apheresis 2011, 26:230-238.
7. Muylle L, Taelman, Moldenhauer R, Brabant R, Petermans M. Usefulness of apheresis to extract microfilaria in management of loasis. British Medical Journal 1983, 287:519-520.
8. Kern P, Wind P, Dietrich M. Continuous-flow apheresis of microfilariae in loa loa infestations. Plasma Therapy and Transfusion Technology 1988, 9:95-98.
9. Zhao Y, Alexander B, Bailey J, Welch L, Greene M, Vauthrin M et al. Therapeutic apheresis using a mononuclear cell program to lower the microfilaria burden of a 23-year-old African woman with loiasis. J Clin Apher. 2016, 10:1002
10. World Health Organization: Severe falciparum malaria. Transactions of Royal Society Tropical Medicine and Hygiene. 2000, 94: S1-S90.
11. Shaw-Saliba K, et al. Infection of laboratory colonies of Anopheles mosquitoes with Plasmodium vivax from cryopreserved clinical isolates. International Journal of Parasitology. 2016, 46:679-83.
12. Ramachandran SD, Schirmer K, Munst B, Heinz S, Ghafoory S, Wolfl S et al. In vitro generation of functional liver organoid-like structures using adult human cells. PLoS ONE 2015, 10:10
13. Vaughan AM, Kappe SH, Ploss A, Mikolajczak SA. Development of humanized mouse models to study human malaria parasite infection. Future Microbiology. 2012, 7:5.
14. McCarthy J, Sekuloski S, Griffin PM, Elliott, S, Douglas N, Peatey C, Rockett R, O'Rourke P, Marquart L, Hersen, C, Duparc S, Moehrle, J, Trenholme KR., and Humberstone AJ. A exploratory randomised trial of induced blood-stage *Plasmodium falciparum* infections in healthy subjects for testing efficacy of new antimalarial drugs. PLoS One 2011.
15. Pombo DJ, Lawrence G, Hirunpetcharat C, Rzepczyk C, Bryden M, Cloonan N, et al. Immunity to malaria after administration of ultra-low doses of red cells infected with *Plasmodium falciparum*. Lancet. 2002 Aug 24: 360(9333):610-7
16. Sanderson F, Andrews L, Douglas AD, Hunt-Cooke A, Bejon P, Hill AV. Blood-stage challenge for malaria vaccine efficacy trials: a exploratory study with discussion of safety and potential value. The American journal of tropical medicine and hygiene. 2008;78(6):878-83.
17. ANZCTR Trial ID: ACTRN12611001203943
18. ANZCTR Trial ID: ACTRN12612000323820
19. ANZCTR Trial ID: ACTRN12612000814875
20. ANZCTR Trial ID: ACTRN12613000698774
21. ANZCTR Trial ID: ACTRN12613000565741
22. ANZCTR Trial ID: ACTRN12613001040752
23. Griffin P., Pasay C., Elliott S., *et al*., Experimental Vivax Transmission to Anopheles (EVITA); A Clinical trial to assess mosquito transmissibility from subjects inoculated with blood stage *Plasmodium vivax*, *Manuscript in preparation*., (2015) (ANZCTR Trial ID: ACTRN12613001008718).
24. ANZCTR Trial ID: ACTRN12614000930684
25. Sighinolfi, L., Libanore, M., Bicocchi, R., Reverberi, R., Bedetti, A., & Ghinelli, F. (1990). Treatment of cerebral malaria by erythrocyte exchange. *Recenti progressi in medicina.* 1990, 81:804-805.
26. Santana-Cabrera L, Fernández Arroyo M, Rodríguez González F, Sánchez Palacios M. Erythrocytapheresis in the emergency management of severe falciparum malaria. *Journal of Emergencies, Trauma and Shock*. 2010, 3: 206.
27. Files JC, Case CJ, Morrison FS. Automated erythrocyte exchange in fulminant falciparum malaria. Ann Intern Med. 1984;100:396.
28. Lercari G, Paganini G, Malfanti L, Rolla D, Machi AM, Rizzo F, et al. Apheresis for severe malaria complicated by cerebral malaria, acute respiratory distress syndrome, acute renal failure, and disseminated intravascular coagulation. J Clin Apher. 1992;7:93-6.
29. Mainwaring CJ, Leach MJ, Nayak N, Green ST, Jones DA, Winfield DA. Automated exchange transfusion for life-threatening Plasmodium falciparum malaria-lessons relating to prophylaxis and treatment. J Infect. 1999, 39:231-3.
30. Macallan DC, Pocock M, Bishop E, Bevan DH, Parker-Williams J, Harrison T, et al. Automated erythrocytapheresis in the treatment of severe falciparum malaria. J Infect. 1999;39: 233-236
31. Tejura B, Sass DA, Fischer RA, Daskal I, Eiger G. Transfusion-associated falciparum malaria successfully treated with red blood cell exchange transfusion. Am J Med Sci. 2000;320: 337-341
32. Zhang Y, Telleria L, Vinetz JM, Yawn D, Rossmann S, Indrikovs AJ. Erythrocytapheresis for Plasmodium falciparum infection complicated by cerebral malaria and hyperparasitemia. J Clin Apher. 2001;16:15-8.
33. Deshpande A, Kalgutkar S, Udani S. Red cell exchange using cell separator (therapeutic erythrocytapheresis) in two children with acute severe malaria. J Assoc Physicians India. 2003, 51: 925-926.
34. Nieuwenhuis JA, Meertens JH, Zijlstra JG, Ligtenberg JJ, Tulleken JE, Van der Werf TS. Automated erythrocytapheresis in severe falciparum malaria: a critical appraisal. Acta Trop. 2006;98: 201-206.
35. Auer-Hackenberg L, Staudinger T, Bojic A, et al. Automated red blood cell exchange as an adjunctive treatment for severe *Plasmodium falciparum* malaria at the Vienna General Hospital in Austria: a retrospective cohort study. *Malaria Journal*. 2012,11:158.
36. Lercari, G, Paganini G, Malfanti L, Rolla D, Machi AM, Rizzo F et al. Apheresis for severe malaria complicated by cerebral malaria, acute respiratory distress syndrome, acute renal failure, and disseminated intravascular coagulation. *Journal of clinical apheresis* 1992,7:93-6.
37. Chung, HS, Peck KR, Kim DW. Two Case Reports of Successful Therapeutic Erythrocytapheresis as an Adjunctive Therapy in Severe Falciparum Malaria. *Therapeutic Apheresis and Dialysis*. 2010, 14: 230–233.
38. Santana-Cabrera L, Fernández Arroyo M, Rodríguez González F, Sánchez Palacios M. Erythrocytapheresis in the emergency management of severe falciparum malaria. *Journal of Emergencies, Trauma and Shock*. 2010,3:206.
39. Calvo-Cano A, Gómez-Junyent J, Lozano M, Castro P, Cid J, Nicolas JM et al. The role of red blood cell exchange for severe imported malaria in the artesunate era: a retrospective cohort study in a referral centre. *Malaria Journal*. 2016;15:216
40. Hickey B, Lumsden J, Reyes S, Sedegah M, Hollingdale M, Frellich D et al. Mosquito bite immunization with radiation-attenuated *Plasmodium falciparum* sporozoites: safety, tolerability, protective efficacy and humoral immunogenicity (2016). *Malaria Journal*, 15:377. DOI 10.1186/s12936-016-1435-y.
41. Wang R, Arevalo-Herrera M, Gardner M, Bonela A, Carlton J, Gomez A et al. Immune responses to *Plasmodium vivax* pre-erythrocytic stage antigens in naturally exposed Duffy-negative humans: a potential model for identification of liver stage antigens (2005). *European Journal of Immunology.* 35:1859-1868.
42. S Chong. Australian Haemovigilance report Data 2013-2014.National blood authority and haemovigilance advisory committee Australia.2016
43. Lee G, Arepally GM. Anticogulation Techniques in Apheresis from Heparin to citrate and beyond. Journal of clinical Apheresis.2012;27:117-125. DOI 10.1002/jca.21222
44. Hölig, K., Kramer, M., Kroschinsky, F., Bornhäuser, M., Mengling, T., Schmidt, A. H., Rutt, C., & Ehninger, G. Safety and efficacy of hematopoietic stem cell collection from mobilized peripheral blood in unrelated volunteers: 12 years of single-center experience in 3928 donors. Blood. 2009;114(18),3757-3763. DOI 10.1182/blood-2009-04-218651
45. Hutton D, Harrison J, Carter M, Morgan S, Edner A, Bianco C et al. Recommendations on apheresis donation in young and first time donors. JPAC 09-16
46. Eder AF, DY BA, Kennedy JM, Notari Iv EP, Strupp A, Wissel ME et al. The American Red Cross donor hemovigilance program: complications of blood donation reported in 2006. *Transfusion*;2008.48(9):1809-19.
47. McCarthy, J. S., Griffin, P. M., Sekuloski, S., Bright, A. T., Rockett, R., Looke, D., Elliott S., Whiley D., Sloots T., Winzeler E. A., Trenholme, K. R. (2013). Experimentally Induced Blood-Stage *Plasmodium vivax* Infection in Healthy Volunteers. *The Journal of Infectious Diseases*, *208*(10), 1688–1694.
48. ANZCTR Trial ID: ACTRN1261600017448
49. Infanti, L. Red cell apheresis: pros and cons, *ISBT Science Series* (2018) 13, 16–22

# APPENDIX

**Appendix 1: Total whole blood volume in addition to Apheresis procedure**

| **Procedure** | **Sample** | **Volume per sample (mL)** | **No. samples per subject for the first 30 days** | **Total volume per subject (mL) for the first 30 days** |
| --- | --- | --- | --- | --- |
| Laboratory Safety Assessment | Haematology  (including G6PD) | 2 | 4 | 8 |
|  |  | 4 | 1 | 4 |
|  | Biochemistry (including LFT) | 5 | 5 | 25 |
|  |  |  |  |  |
|  | Serology | 3.5 | 0 | 0 |
|  | Safety Serum storage | 5 | 1 | 5 |
| Cannulation | Discard | 2 | 8 (+2 if required) | 16 (+4 if required) |
| Malaria Monitoring | Malaria qPCR (18S) | 2 | 15 (+5 if required) | 30 (+10 if required) |
|  | Microscopy | 2 | 0 | 0 |
|  | Parasite life-cycle stage qRT-PCR | 2 | 5 (+3 if required) | 10 (+6 if required) |
| Malaria Research | Immunology/pathophysiology sample | 20 | 0 | 0 |
| MFA (in selected subjects but not those selected for HMP bank production) |  | Up to 80 | Up to 1 | 80 (total over either 1 or 2 time-points) |
| HMP bank blood borne infection testing  (Appendix 7)  (in selected subjects but not those selected for MFA) |  | 55 | Up to 1 | 55 |
| **Study Total (mL) no MFA no HMP Bank** | | | | 98 (+20 if required) |
| **Study Total (mL) MFA no HMP Bank** | | | | 178 (+20 if required) |
| **Study Total (mL) HMP Bank no MFA** | | | | up to - 153 (+20 if required) |

Most subjects (not involved into membrane feeding or HMP bank procedures) will have 118 mL or less of whole blood taken during the first 30 days of the study (post screening) in addition to the 386 mL of red blood cells collected during the Apheresis procedureAn additional 20 mL may be collected on day 90±7.

Subjects involved in membrane feeding or HMP bank procedure will have 200 mL of less of whole blood taken during the first 30 days of the study (post screening) in addition to the 386 mL of red blood cells collected duringt the Apheresis procedure. An additional 75 mL may be collected on day 90±7.

**Appendix 2: Symptoms and Signs of Malaria**

Following challenge via the intravenous malaria challenge inoculation and during the post-challenge period, the following signs and symptoms of malaria will be monitored:

**Signs of Malaria**

Fever (≥38°C)

Chills/Shivering/Rigors

Tachycardia

Hypotension

**Symptoms of Malaria**

Headache

Myalgia (muscle ache)

Arthralgia (joint ache)

Fatigue/lethargy

Malaise (general discomfort/uneasiness)

Sweating/hot spells

Anorexia

Nausea

Vomiting

Abdominal discomfort

**Appendix 4: Antimalarial Rescue drugs approved manufacturer’s prescribing information**

**(Provided as separate documents)**

Chloroquine Product information

Riamet^®^ TGA July 2012

- Product Information
- Consumer Medicine Information

**Appendix 6: Tubes for HMP bank blood borne infection testing**

| **Tube Type and**  **Number of Tubes** | **Tube Lot**  **Number** | **Tube Expiry**  **Date** | **Collection Time and**  **Date** | **Operator initials** |
| --- | --- | --- | --- | --- |
| 5 x 4 mL EDTA |  |  |  |  |
| 3 x 5 mL SST  3 x 5 mL SST |  |  |  |  |
| 4 x 5 mL PPT |  |  |  |  |

**Appendix 7: Version History**

| Version | Date | Author(s)/Reviewer(s) | Significant Revisions |
| --- | --- | --- | --- |
| 1.0 | 29 Aug 2017 | 1. Odedra | Initial version |
| 1.1 | 17 Oct 2017 | R. Watts/A. Odedra | Changed Principal Investigator to Prof. James McCarthy. Made changes in response to the RBWH HREC comments. See summary of changes document for more details. |
| 2.0 | 1 Nov 2017 | R. Watts/A. Odedra | Added an additional exploratory objective for antibody research. Added a baseline blood sample at Day 0 for immunology and pathophysiology malaria research. Changed blood volume for membrane feeding assays to 80 mL total over 1-2 time-points. See summary of changes document for more details. |
| 2.1 | 22 March 2018 | E. Rossignol/ A. Odedra | Addition of an additional co-principal investigator |
| 3.0 | 15 May 2018 | E. Rossignol/ A. Odedra | - Added the possibility to reduce confinement to 48h if the subjects are deemed clinically well by the investigator and are happy to be released early.  - Clarified that apheresis may occur on a day other than day 10. Earlier cohorts results have shown that, 24h before day 10, parasite levels may be much below the 20,000 parasites/mL threshold and the malaria score much below 6.  - Added the possibility to collect samples more frequently for 18s PCR analysis from Day 9 to treatment day.  - Added the possibility to confine subjects from day 9 until treatment day.  - Clarified that the duration of stay at the apheresis unit may be longer than 4h (to allow preparation for the procedure).  - Clarified that immunology exploratory endpoints are optional.  - Modified the inclusion criteria to exclude females. A very good venous access is required for the apheresis procedure and females often fail this criteria. It is deemed inappropriate to invite females for screening, considering the low likelihood that they may be included in the study.  -Corrected the intravenous artesunate treatment duration.  - Clarified that, before exit from confinement, nursing staff must confirm with the study doctor or investigator if any subject requires symptom-driven examination pre discharge. |
| 3.1 | 9 July 2018 | E. Rossignol/ A. Odedra | Addition of an additional co-principal investigator |
| 4.0 | 22 October 2018 | E. Rossignol/ A. Odedra | Modification of the apheresis procedure and increase of the volume of red blood cells collected. |
