# Supplementary material for: Safety and feasibility of apheresis to harvest and concentrate parasites from subjects with induced blood stage Plasmodium vivax infection
Source: Malar J. 2021 Jan 14;20:43. doi: 10.1186/s12936-021-03581-w (PMC7807416; doi:10.1186/s12936-021-03581-w)
Supplement: Supplementary file 2 — Additional file 2. Apheresis Cohort 1 standard operating procedure. [file 12936_2021_3581_MOESM2_ESM.docx]

Apheresis Procedure: QIMR study cohort 1

**Bone Marrow Transplant Program**

**Metro North Health Hospital and Health Service**

**Royal Brisbane & Women’s Hospital**

**Children's Health Queensland Hospital and Health Service**

**Lady Cilento Children’s Hospital**

# Purpose

## This SOP describes how to perform a continuous mononuclear cell (CMNC) procedure. It is intended to be used in conjunction with:

### Spectra Optia Essentials

### Spectra Optia Continuous Mononuclear Cell Collection (CMNC) Procedure Guide

# Scope

## The target audience for this resource are the registered nurses and medical officers who care for patients in the Apheresis Unit, Royal Brisbane & Women’s Hospital (RBWH), who are involved and trained in apheresis.

## Patient safety takes priority during a CMNC collection, therefore only competent nurses can perform this procedure.

## All apheresis procedures will comply with 05009/WUG: Apheresis

## All deviations and associated corrective actions shall be documented in the patient medical record.

# Principle / Background

## Apheresis refers to a number of procedures involving the removal of whole blood, separating it into the various blood components through centrifugation, removing a desired component (white/red cells, platelets and plasma with or without replacement fluids) and returning the rest of the components. Access to the intravascular compartment is required and efficacy of the treatment will depend on the volume of blood processed.

## All patients requiring a CMNC collection are under the direct care of a Haematologist.

## The RBWH Apheresis Unit is accredited by:

### Australian Bone Marrow Donor Registry (ABMDR)

### National Association of Testing Authorities (NATA)

### Foundation for the Accreditation of Cellular Therapies (FACT)

## The Royal Brisbane & Women’s Hospital (RBWH) is accredited by the Australian Council on Healthcare Standards (ACHS)

## The Disaster Plan (Section 11) addresses those elements most considered to be at risk and provides guidelines to cover some of the potential serious emergencies that may arise. All staff involved in apheresis procedures are responsible for following this plan as directed by senior personnel.

# Objectives / Endpoints

## All apheresis procedures shall be performed safely and appropriately.

# Documentation

## **Policy and Standard/s**

### ACHS EQUiP National

### Blood and Blood Products, Management of PROC103 Doc 110/15 (MNHHS)

### Clinical Incident and Disclosure Management PROC007 Document 13/14 (MNHHS)

## **Procedures, Guidelines, Protocols**

### Australian Guidelines for the Prevention and Control of Infection in Healthcare (2010)

### COLGDE001: RBWH Guidelines in the Management of Potential Adverse Events in Apheresis

### 02002/Proc: Medications Management

### 21605/Proc: Blood collection (Adult)

### 05009/Proc: Apheresis

### 05450/Proc: Peripheral Intravenous Cannulation, Venepuncture and Infusions – Adult and Paediatrics

### 74100/Proc: Documentation in the Patient Record

### 81000/Proc: Aseptic Non Touch Technique

### 000342/Proc: Standard Precautions

### 003139/Proc: Cleaning and Decontamination – patient environment and clinical equipment

## **Forms and Templates**

### CLIFRM013: RBWH Donor Consent for Bone Marrow Harvest or Apheresis Procedure

### COLFRM009: RBWH Apheresis Patient Checklist label

### COLFRM028: RBWH Apheresis Service Request

### COLFRM030: RBWH Apheresis Product Collection Form

### COLFRM053: RBWH Apheresis Procedure Run Sheet: Spectra Optia

# Definitions and Abbreviations

## AIM Automated interface management

## ABMDR Australian Bone Marrow Donor Registry

## ACD-A Acid citrate dextrose - solution A

## BTS Biomedical Technical Services

## CMNC Continuous mononuclear cell

## CMV Cytomegalovirus

## CP Collection preference

## ELFT Electrolyte & liver function tests

## FBC Full blood count

## Hb Haemoglobin

## HBV Hepatitis B virus

## HCV Hepatitis C virus

## HIV Human immunodeficiency virus

## HTLV Human T-lymphotropic virus

## IDM Infectious disease marker

## IV Intravenous

## MNC Mononuclear cell

## MNHHS Metro North Hospital and Health Service

## NUM Nurse Unit Manager

## ODTU Oncology Day Therapy Unit

## RBWH Royal Brisbane & Women’s Hospital

## RN Registered nurse

## SOP Standard operating procedure

## TBV Total blood volume

# Materials

## **Equipment**

### Spectra Optia Apheresis System (version 11.3) – CMNC Collection.

### IDL filler

### Electronic blood pressure monitor

### Volumetric infusion pump

### Heat sealer

## **Disposables and Reagents**

### Spectra Optia IDL Set (Ref: 10310).

### ACD-A anticoagulant (750ml)

### 0.9% normal saline (1000ml)

### Alcohol swab

### 3 way taps (2)

### IV cannulation or central line equipment. Refer to procedures: 05450/Proc: Peripheral Intravenous Cannulation, Venepuncture and Infusions – Adult and Paediatrics; 05501/Proc: Cannulation of Haemodialysis Access; 05600/Proc: Central Venous Access Devices (CVADs), Management – Adult. Ensure use of sufficient gauge cannulas (backeye needles are preferable for inlet venous access) to allow for adequate flow required for the procedure (preferably at least 16G for inlet and 18G for return)

### Blood collection tubes

### Intravenous giving set

### Burette

# Special Considerations

## This procedure describes how to perform a CMNC procedure.

## Ensure haematocrit is accurate, as this is used to calculate:

### The limit for collect volumes

### The plasma pump flow rate before the AIM system manages the concentration of cells in the collect port

### Changing the haematocrit **after** the interface has been established will not change the interface position

## The packing factor whilst collecting MNC defaults to 4.5

## No plasma is to be collected

## There **MUST** be FBC and ELFT blood test results available (taken within the previous 24hrs) **PRIOR** to commencement of procedure.

## The minimum FBC criteria required in order to proceed with the procedure is described below.

| **QIMR volunteer** | Platelet > 100 x 10^9^/L |
| --- | --- |
|  | Hb > 100G/L |

## There is a risk of deranged electrolytes due to the effects of the procedure. This effect is usually transient and resolves quickly upon cessation of CMNC collection. Electrolyte replacement may be required. Calcium gluconate is the most frequently required electrolyte replacement and the amount and rate is dependent on symptoms of hypocalcaemia during the procedure. Magnesium and potassium replacement can also be required, below is a guide for when supplementation may be required:

| **Pre-Procedure Electrolyte** | **Replacement guide** |
| --- | --- |
| Magnesium < 60 mmol/L | 10 – 20 mmols IV magnesium sulphate may be required during procedure |
| Potassium between 3.0 – 3.5 mmol/L | Oral potassium may be required e.g 1-2 chlorvescent tablets |
| Potassium < 3.0 mmol | IV potassium chloride 20 mmols may be required during the procedure and / or 3 oral chlorvescent |

# Procedure

## Confirm the following forms are current and valid:

### CLIFRM013: RBWH Donor Consent for Bone Marrow harvest or Apheresis Procedure

### COLFRM028: RBWH Apheresis Service Request

## Prior to commencement of procedure, ensure patient has had appropriate IDMs taken.

### HIV serology

### Hepatitis B serology

### Hepatitis C serology

### Syphilis

## All stock used to collect the product must be visually examined prior to use for signs of damage and contamination, as per COLSOP004: Apheresis Inventory Control Procedure. Record expiry date and lot number of all stock used on COLFRM030: RBWH Apheresis Product Collection form. Check expiry dates and lot numbers of procedure kit and intravenous fluid bags with 2^nd^ RN and record on COLFRM053: RBWH Apheresis Procedure Run Sheet: Spectra Optia

### Confirm that all consumables and reagents used during the procedure are satisfactory for use; inspect for damage or evidence of contamination and mark the appropriate check box on COLFRM030: RBWH Apheresis Product Collection form

## Set up, load and prime Spectra Optia IDL Set (Ref: 10310) as per on screen instructions

## Follow machine prompts and enter patient data.

## Educate patient of signs and symptoms of potential adverse reactions, emphasising importance of informing nursing staff if he/she experiences any such reactions

## Perform baseline observations (temperature, pulse, respirations and blood pressure) and record on run sheet

## Ensure COLFRM009: RBWH Apheresis Patient Checklist has been completed

## Perform Cannulation according to 05450/Proc: Peripheral Intravenous Cannulation, Venepuncture and Infusions – Adult and Paediatrics

## Collect blood samples if required.

## Follow machine prompts to commence procedure

## Review run screen to ensure pump flow rates are appropriate for the procedure. Monitor the AC infusion rate, ensuring that it does not exceed 0.9 ml/min

## The Spectra Optia system will attempt to establish the interface at commencement of the procedure, during this time, the collect valve will remain closed.

## The AIM system monitors the collect port and changes the plasma pump flow rate to manage the concentration of cells that flow through the collect port. When the AIM system detects cells in the collect port, the collect valve opens and starts collecting. **Ensure the collect flow rate is set at 3ml/min**

## The CP defaults to 50 at commencement of procedure. The apheresis nurse can set the CP to collect lighter or darker from the buffy coat. Use the collection preference tool as a guide for the appropriate CP.

### **See appendix 1 for the appropriate CP, using the collection preference tool.**

### A higher CP results in collecting lighter in the buffy coat

### A lower CP results in collecting darker in the buffy coat

## Monitor and record patient’s vital signs and run parameters q30 minutes or as clinically indicated. Monitor for signs of adverse reactions and record on procedure run sheet, reporting adverse events on PRIME.

## Ensure patient comfort and safety throughout the procedure.

## Apply a patient ID label to the collection bag. ***THE LABELS MUST BE APPLIED TO THE COLLECT BAG BEFORE THE PATIENT OR THE COLLECT BAG ARE DISCONNECTED FROMTHE PROCEDURE KIT.***

## At the end of the procedure, follow the system prompts to rinseback

## Seal the collection bags using a heat sealer prior to disconnection of the patient. ***Ensure that there is approximately 20cm of sterile dockable tubing remaining after heat sealing.***

## The RN performing the collections shall record the date, time of completion and sign the collection bag label. Verify the identity of the patient with the patient ID label.

## Contact QIMR to collect the cells. The time of collection completion shall be recorded on COLFRM030: RBWH Apheresis Product Collection form. The collection bags must be placed in a sealed plastic bag for transport to QIMR.

## Release of the product from the Apheresis nurse to the QIMR scientist is recorded on COLFRM030: RBWH Apheresis Product Collection form. The Apheresis nurse releasing the collected product, must sign (stating time and date) that the product has been released, the QIMR scientist accepting the collected product must sign (stating date and time) that they have accepted product quarantine. Glove must be worn by all staff handling the product.

## On completion of rinseback, collect routine blood samples (usually FBC and ELFT).

## Perform final set of observations and record on procedure run sheet

## Remove venous access as appropriate

## Record final values on COLFRM053: RBWH Apheresis Procedure Run Sheet and complete COLFRM030: RBWH Apheresis Product Collection form.

## Once paperwork is complete, scan / photocopy all procedural paperwork and send to QIMR.

## Unload set and dispose of waste appropriately

## Decontaminate Spectra Optia as per protocol (see COLSOP005: RBWH Apheresis Equipment Cleaning Procedure). Decontaminate all other equipment involved in performing the procedure, in accordance with 003139/Proc: Cleaning and Decontamination – patient environment and clinical equipment. Once all equipment used during the procedure has been cleaned, tick appropriate area at the end of the run sheet, confirming completion of cleaning tasks.

## Document all care given in patient medical notes. Include all completed paperwork with patient’s medical notes.

## Record relevant statistical information in: G:\Oncology\Apheresis\Log and Statistics

# End Points

## A maximum of 500mls is to be collected, unless clinically indicated

# Disaster Plan

## This section describes the actions to be taken in the event of serious problems which may arise in the Apheresis Unit which may impact on either:

### The ability to perform the CMNC collection to the required specification

### The quality of the collected product

## **Air-Conditioning System Failure** – in the event that the air-conditioning system malfunctions, contact hospital maintenance and arrange for urgent repairs (ext. 67963).

## **Loss of Power** – All critical equipment used to perform apheresis procedures should be connected to red emergency power points. These power points are supplied with the electricity from the emergency backup power generators, and in the event of a power failure, are automatically activated.

### Critical equipment used within the apheresis unit includes:

- - - 1. Cell separators
      2. Blood warmers
      3. Electronic blood pressure monitors
      4. Intravenous volumetric pumps
      5. Heat sealers

### In the event of an interruption to normal power supply, there may be a temporary loss of power before the emergency generator is activated. If there is an interruption of the power supply:

- - - 1. Close clamps on collect line immediately
      2. If the emergency power supply is activated, power will resume, and the Spectra Optia system will reset. After system resets, a screen will appear with instructions for how to proceed.
      3. To restart the procedure, follow onscreen instructions
      4. To abort procedure, press ***RINSEBACK*** (if appropriate) or ***DISCONNECT*** and follow on screen instructions

### In the event of a complete loss of power with no emergency power supply:

- - - 1. The Spectra Optia cell separator will shut down completely. Close clamps on collect line immediately. Seal the collection bags using a heat sealer (these should still be functional if battery charged)
      2. Contact the patient physician and inform him of events and that the procedure may be aborted
      3. Consult with the Director of the Haematology & BMT
      4. The extracorporeal volume in the kit will be 297ml
      5. The patient may be disconnected without performing ***RINSEBACK*** or perform ***MANUAL RINSEBACK*** as per instructions in ***Spectra Optia Essentials Guide, pp.73-76*** (in the event that a blood prime was performed, ***RINSEBACK*** is not necessary)

## **Immediate evacuation** – in the event that an immediate evacuation is required:

### Close clamps on collect line if possible. NURSE AND PATIENT SAFETY IS PARAMOUNT, therefore do not complete this step if completion may endanger the health and safety of the nurse or the patient

### Disconnect patient immediately and evacuate as per [RBWH Code Orange Evacuation procedure](http://hi.bns.health.qld.gov.au/medical/emergencies/default.htm). **N.B.** since RINSEBACK has not been performed, the patient will have a 297ml fluid deficit and may become hypotensive, monitor patient accordingly

## **Structural damage (e.g. fire, water / flood damage)** – in the event of structural damage, relocate product, material and patient files as required, to another area of ODTU if possible. Depending on the scale of the problem, a final decision on a temporary location may need to be determined on a case-by-case assessment by a structural engineer and the Director of the BMT program

### All critical equipment and/or consumables which may have incurred damage should be discarded and replaced as soon as possible

### Contact hospital maintenance (ext. 67963) and organise for them to inspect critical equipment as soon as possible

### Suspend all operational activities until repairs are complete and the facility is signed off as safe to reoccupy

## **Critical equipment failure –** Perform troubleshooting as per relevant equipment manuals. If unable to resolve problem, contact BTS or manufacturer representative.

## **Critical consumable shortage –** if a critical consumable shortage occurs, contact the following Apheresis Units to source consumables:

### Greenslopes Private Hospital (Speed dial *2521).

### Lady Cilento Childrens’ Hospital (Jo Ritchie – 3068 5686, or Jill Shergold – 3068 5767)

### Once alternative supply has been sourced, organise transport of the required consumables. Liaise with the NUM, ODTU to organise payment of delivery e.g. courier service

## **Critical staff shortage –** The Apheresis Nurse Practitioner shall liaise with the NUM, ODTU regularly to ensure staffing requirements of the apheresis unit are met. In the event that key personnel are unavailable to perform critical procedures:

### Contact Apheresis Nurse Practitioner (pager# 26299) and NUM, ODTU (ext. 68754 / 73991) informing them of the situation

### The NUM / Apheresis Nurse Practitioner will attempt to adequately staff the Apheresis Unit

### If appropriate, the situation can be escalated to the Director of the BMT program and the Nursing Director, CCS

### If adequate staffing cannot be accomplished, the Apheresis Nurse Practitioner shall liaise with the Director of the BMT Program to discuss requesting the assistance of other apheresis units in the area. Alternative units may be affiliated with the RBWH e.g. Greenslopes Private Hospital, or other similarly accredited Apheresis Units e.g. Princess Alexandra Hospital or the Mater Public Hospital

# Responsibility

|  | Final Approval | Implement | Quality Assurance | Review | Perform Procedure |
| --- | --- | --- | --- | --- | --- |
| Apheresis Nurse Practitioner |  | X | X | X | X |
| Medical Director | X | X |  |  |  |
| Nursing Staff |  |  |  |  | X |

# Occupational Health and Safety

## Standard precautions shall be maintained throughout the procedure. Refer to 000342/Proc: Standard Precautions.

# Risk

## Low to Medium (case dependant).

# Consultation

## Director Bone Marrow Transplant – Glen Kennedy

## Apheresis Nurse Practitioner Kari Mudie

## **Appendix 1 – Collection preference guide**


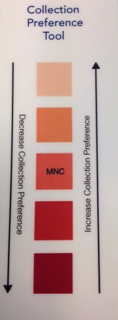


**1%**

**3%**

**7.5%**

**2%**

**5%**

**END OF PROCEDURE**
