# Supplementary material for: Safety and feasibility of apheresis to harvest and concentrate parasites from subjects with induced blood stage Plasmodium vivax infection
Source: Malar J. 2021 Jan 14;20:43. doi: 10.1186/s12936-021-03581-w (PMC7807416; doi:10.1186/s12936-021-03581-w)
Supplement: Supplementary file 6 — Additional file 6. Supplementary Material v1.0. [file 12936_2021_3581_MOESM6_ESM.docx]

**Safety and feasibility of apheresis to harvest and concentrate parasites in subjects with induced blood stage *Plasmodium vivax* infection supplementary material**

**Fig. 1 Design of apheresis study**


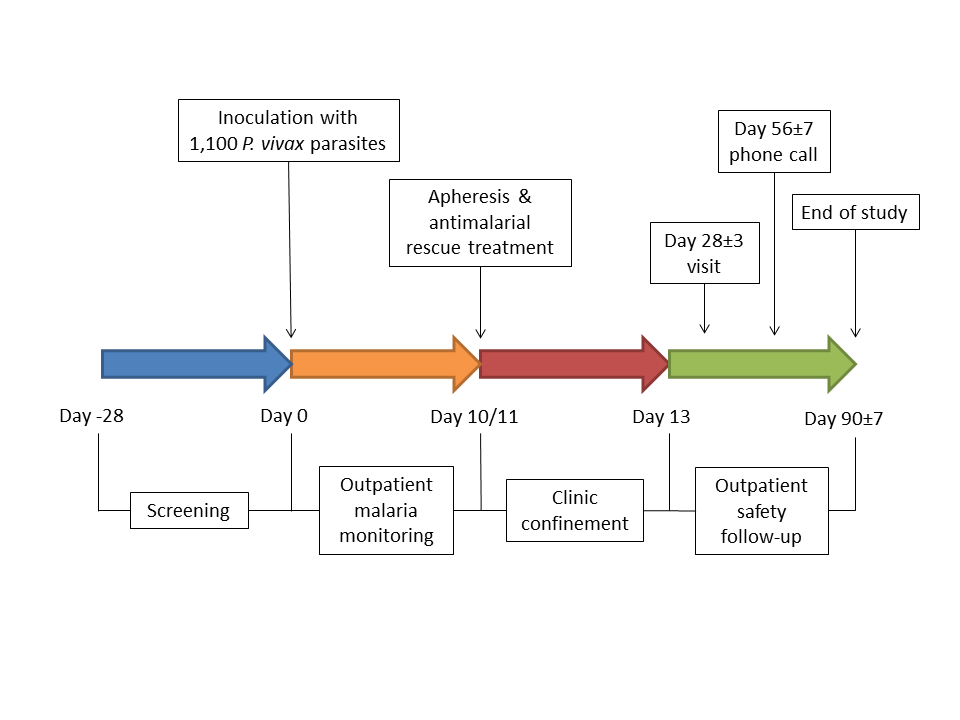


Schematic illustrating the main study activities.

**Fig. 2 Fold enrichment of *P. vivax* parasites determined by 18S qPCR/RBC**

Fold enrichment of *P. vivax* parasites corrected for red blood cell counts as determined by 18S qPCR in samples collected using apheresis compared to the pre-apheresis sample in subjects 1, 2 and 3 (A) and subject 4 (B).

**Figure 3. Schematic of cohort 4 sampling**


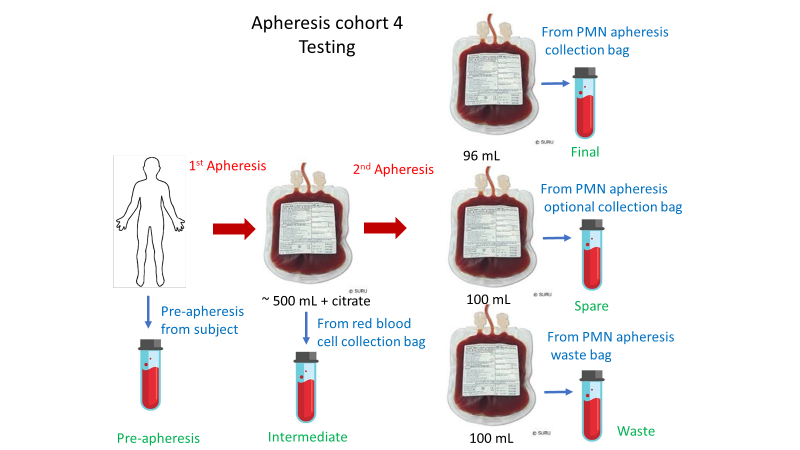


Schematic illustrating the origins of the pre-apheresis, intermediate, final, spare and waste samples collected during cohort 4. Cohort 4 involved two consecutive apheresis procedures. The first was a red blood cell depletion protocol resulting in production of the intermediate bag (~ 500 ml 64% HCT). A polymorphonuclear (PMN) collection was then conducted on this red blood cell collection bag and involved sampling of ~ 96 ml of the lowest HCT layers of the sample (final bag 3% HCT) followed by ~ 100 ml of the subsequent lowest HCT layers (spare bag 5% HCT) and then the remainder ~ 100 ml (waste bag 42% HCT). PMN; polymorphonuclear.

**Fig. 4 Fold Enrichment female *P. vivax* gametocytes determined by *pvs25/*RBC.**

Fold enrichment of female *P. vivax* gametocytes corrected for RBC counts determined by *pvS25* collected using apheresis compared to the pre-apheresis sample in subjects 1 , 2 and 3 (A) and subject 4 (B).

**Fig. 5 Fold Enrichment of *P. falciparum* parasites in pre-clinical experiment.**

Fold enrichment of parasites seen in hematocrit layer samples taken during apheresis compared to the pre-apheresis sample. 5A Fold enrichment of asexual *P. falciparum* parasites as measured by 18S qPCR, 5B Fold enrichment of female *P. falciparum* gametocytes as measured by *pfs25* qRT PCR/ml, and 5C Fold enrichment of male *P. falciparum* gametocytes as measured by *pfMGET* qRT PCR.

**Table 1. Pre-clinical experiment *P. falciparum* asexual parasites/ml**

| **Sample Type** | **asexual parasites (18S qPCR/ml)** | **Fold enrichment asexual parasites compared to pre-apheresis** |
| --- | --- | --- |
| **Pre-apheresis** | 9,547,013,966 | 1 |
| **1% HCT** | 12,150,153,926 | 1.27 |
| **2% HCT** | 3,907,293,793 | 0.41 |
| **3% HCT** | 2,061,766,413 | 0.22 |
| **5% HCT** | 1,153,158,327 | 0.12 |
| **7% HCT** | 689,601,796 | 0.07 |

Fold enrichment of asexual parasites as measured by 18S qPCR/ml seen in hematocrit layer samples taken during apheresis compared to the pre-apheresis sample for asexual *P. falciparum* parasites.

**Table 2. Pre-clinical experiment *P. falciparum* female gametocytes/ml**

| **Sample Type** | **female Gametocytes (*pfS25*/ml)** | **fold enrichment female gametocytes compared to pre-apheresis** |
| --- | --- | --- |
| **Pre-apheresis** | 3,626,818 | 1 |
| **1% HCT** | 13,247,518 | 3.65 |
| **2% HCT** | 5,669,575 | 1.56 |
| **3% HCT** | 2,652,747 | 0.73 |
| **5% HCT** | 1,446,435 | 0.40 |
| **7% HCT** | 719,056 | 0.20 |

Fold enrichment of female gametocytes as measured by *pfS25*/ml seen in hematocrit layer samples taken during apheresis compared to the pre-apheresis sample for *P. falciparum* female gametocytes.

**Table 3. Pre-clinical experiment *P. falciparum* male gametocytes/ml**

| **Sample Type** | **Male Gametocytes (*pfMGET*/ml)** | **fold enrichment male gametocytes compared to pre-apheresis** |
| --- | --- | --- |
| **Pre-apheresis** | 1,795,051 | 1 |
| **1% HCT** | 14,370,397 | 8.01 |
| **2% HCT** | 4,265,936 | 2.38 |
| **3% HCT** | 1,917,837 | 1.07 |
| **5% HCT** | 1,114,766 | 0.62 |
| **7% HCT** | 508,801 | 0.28 |

Fold enrichment of male gametocytes as measured by *pfMGET*/ml seen in hematocrit layer samples taken during apheresis compared to the pre-apheresis sample for *P. falciparum* male gametocytes.

**Figure 6. *P. falciparum* pre-clinical experiment.**

6A microscopy of samples taken during apheresis compared to the pre-apheresis sample. Microscopy is split by *P. falciparum* asexual parasites and gametocytes. 6B gametocyte stages identified during microscopy of samples taken during apheresis. In the 2% HCT, 7% HCT and pre-apheresis samples gametocytes were not observed by microscopy.

**Table 4. Pre-clinical experiment *P. falciparum* parasitemia**

| **Sample** | **Gametocytemia** | | **Asexual parasitemia** | |
| --- | --- | --- | --- | --- |
|  | **%** | **Fold enrichment compared to pre-apheresis** | **%** | **Fold enrichment compared to pre-apheresis** |
| **Pre-apheresis** | 0.01 | N/A | 0.1 | N/A |
| **1% HCT** | 0.76 | 76 | 0.06 | 0.6 |
| **2% HCT** | 0 | N/A | 0.27 | 2.7 |
| **3% HCT** | 0.17 | 17 | 0.03 | 0.3 |
| **5% HCT** | 0.1 | 10 | 0.1 | 1 |
| **7% HCT** | 0 | N/A | 0.1 | 1 |

Microscopy of samples taken during apheresis compared to the pre-apheresis sample. Microscopy is split by *P. falciparum* asexual parasitemia and gametocytemia as defined by % of infected red blood cells.

**Table 5. Pre-clinical experiment *P. falciparum* parasitemia**

| **Sample** | **% Stage 1** | **% Stage 2** | **% Stage 3** | **% Stage 4** |
| --- | --- | --- | --- | --- |
| **1% HCT** | 37.5 | 25 | 25 | 12.5 |
| **2% HCT** | N/A | N/A | N/A | N/A |
| **3% HCT** | 0 | 0 | 100 | 0 |
| **5% HCT** | 0 | 100 | 0 | 0 |
| **7% HCT** | N/A | N/A | N/A | N/A |

Microscopy staging of *P. falciparum* gametocytes identified insamples taken during apheresis. In the samples collected pre-apheresis and from the 2% HCT, 7% HCT layers gametocytes were not observed by microscopy.

**Table 6. Haematocrit and Red cell counts**

| **Subject** | **Sample Type** | **Actual HCT** | **Red Cell Count (× 10^6^/L)** |
| --- | --- | --- | --- |
| **1** | **Pre-apheresis** | 0.38 | 4.35 × 10^3^ |
|  | **1% HCT** | 0.01 | 1.40 × 10^2^ |
|  | **2% HCT** | 0.02 | 1.90 × 10^2^ |
|  | **3% HCT** | 0.03 | 3.00 × 10^2^ |
|  | **5% HCT** | 0.04 | 4.30 × 10^2^ |
|  | **7% HCT** | 0.05 | 5.90 × 10^2^ |
| **2** | **Pre-apheresis** | 0.41 | 4.81 × 10^3^ |
|  | **1% HCT** | 0.01 | 2.00 × 10^2^ |
|  | **2% HCT** | 0.03 | 3.50 × 10^2^ |
|  | **3%HCT** | 0.03 | 2.60 × 10^2^ |
|  | **5% HCT** | 0.03 | 4.30 × 10^2^ |
|  | **7% HCT** | 0.05 | 5.00 × 10^2^ |
| **3** | **Pre-apheresis** | 0.44 | 5.34 × 10^3^ |
|  | **0.5% HCT** | 0.01 | 7.00 × 10 |
|  | **1% HCT** | 0.01 | 1.40 × 10^2^ |
|  | **2% HCT** | 0.01 | 1.50 × 10^2^ |
|  | **3% HCT** | 0.03 | 3.50 × 10^2^ |
|  | **5% HCT** | 0.04 | 4.40 × 10^2^ |
|  | **7% HCT** | 0.07 | 7.70 × 10^2^ |
|  | ***8% HCT** | 0.11 | 1.23 × 10^3^ |
|  | **2-3% HCT** | 0.02 | 2.60 × 10^2^ |
|  | **5-7% HCT** | 0.05 | 6.10 × 10^2^ |
|  | **0.5-8%* HCT** | 0.04 | 4.50 × 10^2^ |
| **4** | **Pre-apheresis** | 0.46 | 5.22 × 10^3^ |
|  | **Intermediate sample** | 0.64 | 7.18 × 10^3^ |
|  | **Final sample** | 0.03 | 2.90 × 10^2^ |
|  | **Spare sample** | 0.05 | 4.80 × 10^2^ |
|  | **Waste sample** | 0.42 | 3.94 × 10^3^ |

*8% HCT was subsequently renamed as 11% HCT given the Sysmex HCT result

Haematocrit and red cell count results for samples taken pre-apheresis and during apheresis for subjects 1 to 4.

**Fig. 7 RBC/WBC ratio in apheresis samples**

7A RBC/WBC ratio results for samples taken pre-apheresis and from the 1% HCT, 2% HCT, 3% HCT, 5% HCT and 7% HCT layers during apheresis from subjects 1 to 3. The apheresis HCT layers selected for the figure were those that were sampled across all 3 subjects. Subject 4 was not included as the composition of samples differed significantly from subjects 1 to 3. 7B RBC/WBC ratio results for samples taken pre-apheresis and during apheresis from subject 4.

**Table 7. *P. vivax* asexual parasites/ml and asexual parasites/RBC apheresis clinical study**

| **Subject** | **Sample Type** | **Asexual parasites/ml** | **fold enrichment asexual parasites compared to pre-apheresis** | **Red Cell Count** | **asexual parasites/RBC** | **fold enrichment asexual parasites/RBC compared to pre-apheresis** |
| --- | --- | --- | --- | --- | --- | --- |
| **1** | **Pre-apheresis** | 11,497 | 1 | 4.35E+09 | 2.64E-06 | 1.00 |
|  | **1% HCT** | 27,394 | 2.38 | 1.40E+08 | 1.96E-04 | 74.03 |
|  | **2% HCT** | 37,064 | 3.22 | 1.90E+08 | 1.95E-04 | 73.81 |
|  | **3% HCT** | 41,945 | 3.65 | 3.00E+08 | 1.40E-04 | 52.90 |
|  | **5% HCT** | 46,105 | 4.01 | 4.30E+08 | 1.07E-04 | 40.57 |
|  | **7% HCT** | 56,180 | 4.89 | 5.90E+08 | 9.52E-05 | 36.03 |
| **2** | **Pre-apheresis** | 24,100 | 1 | 4.81E+09 | 5.01E-06 | 1.00 |
|  | **1% HCT** | 21,409 | 0.89 | 2.00E+08 | 1.07E-04 | 21.36 |
|  | **2% HCT** | 27,022 | 1.12 | 3.50E+08 | 7.72E-05 | 15.41 |
|  | **3%HCT** | 34,406 | 1.43 | 2.60E+08 | 1.32E-04 | 26.41 |
|  | **5% HCT** | 38,646 | 1.6 | 4.30E+08 | 8.99E-05 | 17.94 |
|  | **7% HCT** | 43,480 | 1.8 | 5.00E+08 | 8.70E-05 | 17.36 |
| **3** | **Pre-apheresis** | 25,475 | 1 | 5.34E+09 | 4.77E-06 | 1.00 |
|  | **0.5% HCT** | 46,009 | 1.81 | 7.00E+07 | 6.57E-04 | 137.77 |
|  | **1% HCT** | 46,212 | 1.81 | 1.40E+08 | 3.30E-04 | 69.19 |
|  | **2% HCT** | 50,077 | 1.97 | 1.50E+08 | 3.34E-04 | 69.98 |
|  | **3% HCT** | 68,441 | 2.69 | 3.50E+08 | 1.96E-04 | 40.99 |
|  | **5% HCT** | 51,600 | 2.03 | 4.40E+08 | 1.17E-04 | 24.58 |
|  | **7% HCT** | 80,024 | 3.14 | 7.70E+08 | 1.04E-04 | 21.78 |
|  | **11% HCT** | 100,951 | 3.96 | 1.23E+09 | 8.21E-05 | 17.20 |
|  | **2/3% HCT** | 90,662 | 3.56 | 2.60E+08 | 3.49E-04 | 73.09 |
|  | **5/7% HCT** | 95,699 | 3.76 | 6.10E+08 | 1.57E-04 | 32.89 |
|  | **0.5-11% HCT** | 94,785 | 3.72 | 4.50E+08 | 2.11E-04 | 44.15 |
| **4** | **Pre-apheresis** | 94,965 | 1 | 5.22E+09 | 1.82E-05 | 1.00 |
|  | **Intermediate** | 108,041 | 1.15 | 7.18E+09 | 1.50E-05 | 0.83 |
|  | **Final** | 107,251 | 1.14 | 2.90E+08 | 3.70E-04 | 20.33 |
|  | **Spare** | 74,149 | 0.79 | 4.80E+08 | 1.54E-04 | 8.49 |
|  | **Waste** | 36,059 | 0.38 | 3.94E+09 | 9.15E-06 | 0.50 |

*P. vivax* asexual parasites/ml and asexual parasites/RBC apheresis clinical study data from subjects 1 to 4 including fold enrichments as measured by 18S qPCR.

**Table 8. *P. vivax* gametocytes/ml and gametocytes/RBC apheresis clinical study**

| **Subject** | **Sample** | **gametocytes/ml** | **fold enrichment gametocytes/ml compared to pre-apheresis** | **Red Cell Count** | **gametocytes/RBC** | **fold enrichment gametocytes/RBC compare to pre-apheresis** |
| --- | --- | --- | --- | --- | --- | --- |
| **1** | **Pre-apheresis** | 26,458 | 1.00 | 4.35E+09 | 6.08E-06 | 1.00 |
|  | **1% HCT** | 454 | 0.02 | 1.40E+08 | 3.24E-06 | 0.53 |
|  | **2% HCT** | 952 | 0.04 | 1.90E+08 | 5.01E-06 | 0.82 |
|  | **3% HCT** | 1,186 | 0.04 | 3.00E+08 | 3.95E-06 | 0.65 |
|  | **5% HCT** | 1,388 | 0.05 | 4.30E+08 | 3.22E-06 | 0.53 |
|  | **7% HCT** | 1,146 | 0.04 | 5.90E+08 | 1.94E-06 | 0.32 |
|  | **Percoll** | 9,396 | 0.36 | N/A | N/A | N/A |
| **2** | **Pre-apheresis** | 88,670 | 1.00 | 4.81E+09 | 1.84E-05 | 1.00 |
|  | **1% HCT** | 3,216 | 0.04 | 2.00E+08 | 1.61E-05 | 0.87 |
|  | **2% HCT** | 2,871 | 0.03 | 3.50E+08 | 8.20E-06 | 0.44 |
|  | **3%HCT** | 2,246 | 0.03 | 2.60E+08 | 8.64E-06 | 0.47 |
|  | **5% HCT** | 2,429 | 0.03 | 4.30E+08 | 5.65E-06 | 0.31 |
|  | **7% HCT** | 3,907 | 0.04 | 5.00E+08 | 7.81E-06 | 0.42 |
|  | **Percoll** | 2,064,260 | 23.28 | N/A | N/A | N/A |
| **3** | **Pre-apheresis** | 114,495 | 1.00 | 5.34E+09 | 2.14E-05 | 1.00 |
|  | **0.5% HCT** | 9,283 | 0.08 | 7.00E+07 | 1.33E-04 | 6.19 |
|  | **1% HCT** | 12,573 | 0.11 | 1.40E+08 | 8.98E-05 | 4.19 |
|  | **2% HCT** | 10,983 | 0.10 | 1.50E+08 | 7.32E-05 | 3.42 |
|  | **3% HCT** | 9,390 | 0.08 | 3.50E+08 | 2.68E-05 | 1.25 |
|  | **5% HCT** | 8,100 | 0.07 | 4.40E+08 | 1.84E-05 | 0.86 |
|  | **7% HCT** | 10,999 | 0.10 | 7.70E+08 | 1.43E-05 | 0.67 |
|  | **11% HCT** | 13,472 | 0.12 | 1.23E+09 | 1.10E-05 | 0.51 |
|  | **2/3% HCT** | 11,035 | 0.10 | 2.60E+08 | 4.24E-05 | 1.98 |
|  | **5/7% HCT** | 9,097 | 0.08 | 6.10E+08 | 1.49E-05 | 0.70 |
|  | **0.5-11% HCT** | 15,054 | 0.13 | 4.50E+08 | 3.35E-05 | 1.56 |
|  | **Percoll** | 3,618,500 | 44.8 | N/A | N/A | N/A |
| **4** | **Pre-apheresis** | 547,975 | 1.00 | 5.22E+09 | 1.05E-04 | 1.00 |
|  | **Intermediate** | 794,329 | 1.45 | 7.18E+09 | 1.11E-04 | 1.05 |
|  | **Final** | 102,192 | 0.19 | 2.90E+08 | 3.52E-04 | 3.36 |
|  | **Spare** | 304,977 | 0.56 | 4.80E+08 | 6.35E-04 | 6.05 |
|  | **Waste** | 90,743 | 0.17 | 3.94E+09 | 2.30E-05 | 0.22 |

*P. vivax* gametocytes/ml and gametocytes/RBC apheresis clinical study data from subjects 1 to 4 including fold enrichments as measured by *pvS25* qPCR.

**Fig. 8 *P. vivax* asexual parasites and gametocytes pre and post apheresis.**

Change in *P. vivax* asexual parasites/ml (8A) and gametocytes/ml (8B) from whole blood samples taken from subjects pre and post-apheresis, as measured by 18S qPCR/ml and *pvS2* qPCR respectively.

**Fig. 9 Flow cytometry in Subject 1.**

Flow cytometry single staining for SYBR green (9A) and CD45 (9B) in subject 1.

**Fig. 10 Subject 2 flow cytometry findings CD45 and SYBR green stains**

**Pre-apheresis**

**1% HCT**


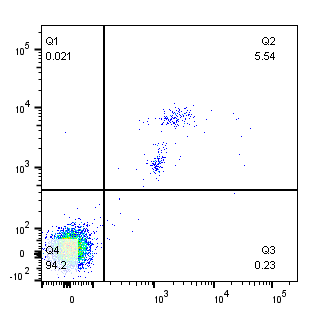

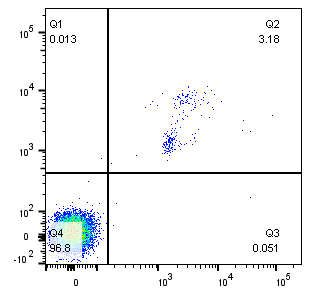

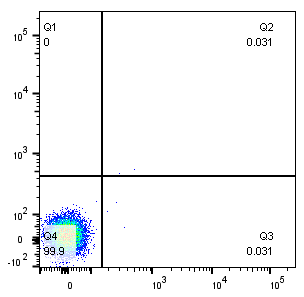


**2% HCT**

**CD45 (405-50 nm)**


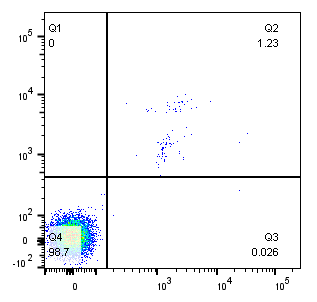

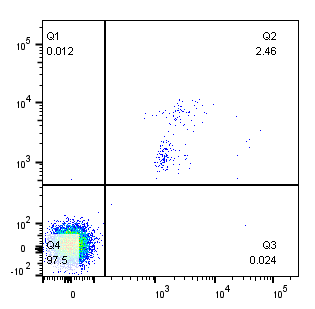

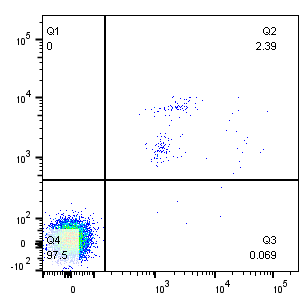


**3% HCT**

**5% HCT**

**7% HCT**

**SYBR green (530-30 nm)**

**Fig. 11 Subject 3 flow cytometry findings CD45 and SYBR green stains**


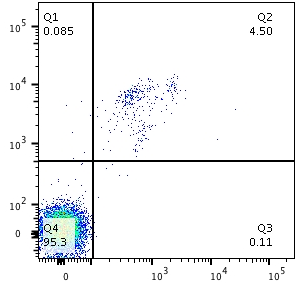

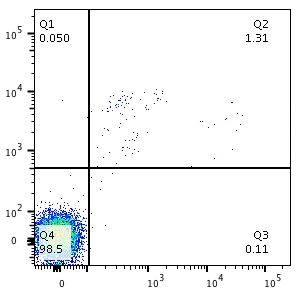

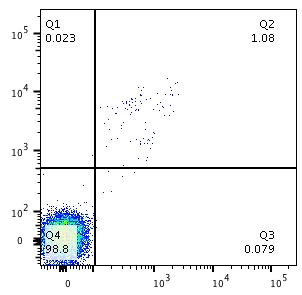

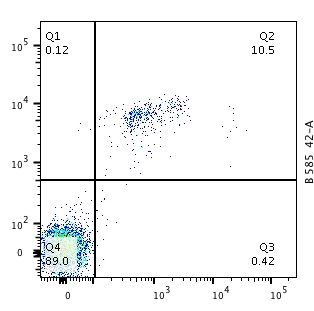

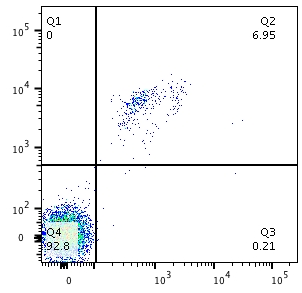

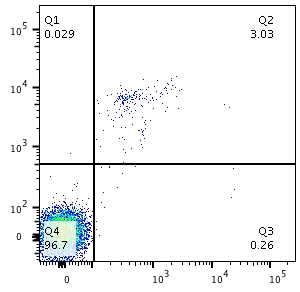

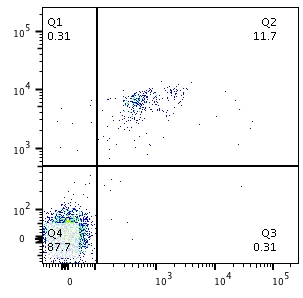


**1% HCT**

**2% HCT**

**3% HCT**

**0.5% HCT**

**CD45 (405-50 nm)**

**11% HCT**

**7% HCT**

**5% HCT**

**SYBR green (530-30 nm)**

**Pre-apheresis**

**Uninfected RBCs**


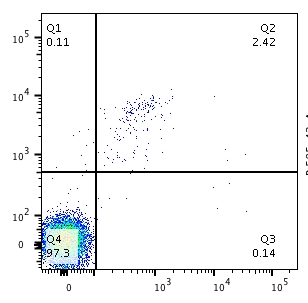

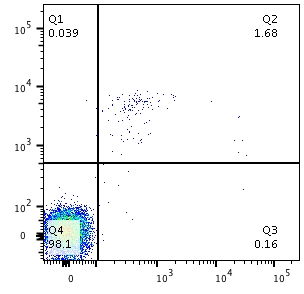

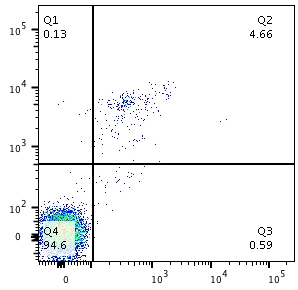

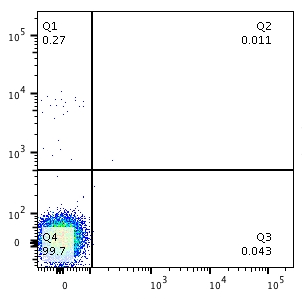

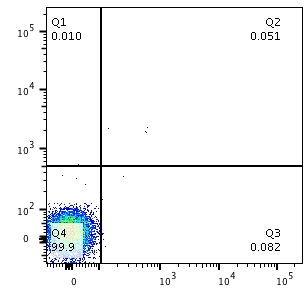


**CD45 (405-50 nm)**

**Pool 0.5-11% HCT**

**Pool 5-7% HCT**

**Pool 2-3% HCT**

**SYBR green (530-30 nm)**

**Fig. 12 Subject 4 flow cytometry findings CD45 and SYBR green stains**

**Intermediate bag (64% HCT)**

**Pre-apheresis (46% HCT)**

**Uninfected RBCs**


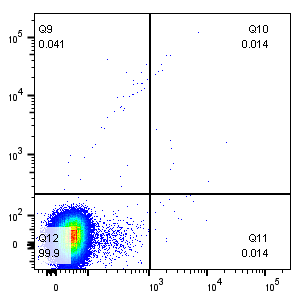

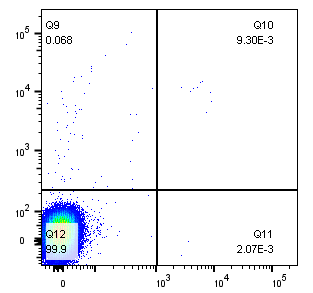

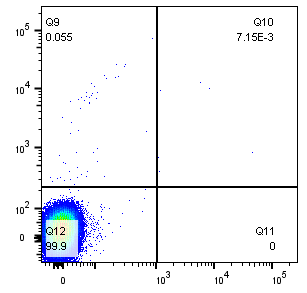

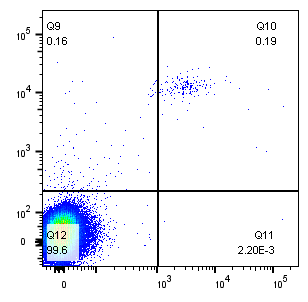


**CD45 (405-50 nm)**


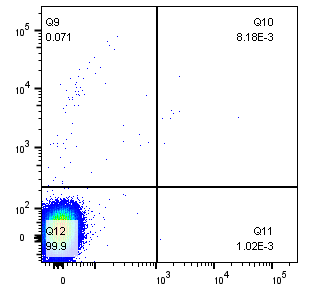

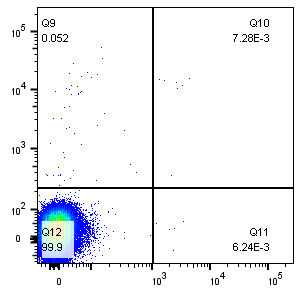


**Waste bag (42% HCT)**

**Spare bag (5% HCT)**

**Final Bag (3% HCT)**

**SYBR green (530-30 nm)**

**Table 9. *P. vivax* microscopy findings for subjects 1 to 3**

| **Sample** | **Subject 1** | | **Subject 2** | | | | ***Subject 3** | |
| --- | --- | --- | --- | --- | --- | --- | --- | --- |
|  |  | | **Reader 1** | | **Reader 2** | |  | |
|  | **Thick film** | **Thin film** | **Thick film** | **Thin film** | **Thick film** | **Thin film** | **Thick film** | **Thin film** |
| **Pre-apheresis** | unreadable | 2 rings | 2 rings | 0 | 3 rings | 0 | 17 | 0 |
| **Percoll** | unreadable | 1 ring | n/a | 0 | n/a | 0 | 70 | unreadable |
| **0.5% HCT** | unreadable | n/a | n/a | n/a | n/a | n/a | unreadable | unreadable |
| **1% HCT** | unreadable | 2 rings | 8 rings | 0 | 12 rings | 1 ring | unreadable | unreadable |
| **2% HCT** | unreadable | 1 ring | 1 ring | 1 ring | 2 rings | 0 | unreadable | 1 |
| **3% HCT** | unreadable | 0 | 0 | 1 ring | 0 | 0 | unreadable | 7 |
| **5% HCT** | unreadable | 0 | 1 ring | 0 | 1 ring | 0 | unreadable | 4 |
| **7% HCT** | unreadable | 0 | 1 ring | 0 | 1 ring | 0 | unreadable | 1 |
| **11% HCT** | unreadable | n/a | n/a | n/a | n/a | n/a | unreadable | 0 |
| **Pool 2-3%** | unreadable | n/a | n/a | n/a | n/a | n/a | unreadable | 12 |
| **Pool 5-7%** | unreadable | n/a | n/a | n/a | n/a | n/a | unreadable | 6 |
| **Pool 0.5-11%** | unreadable | n/a | n/a | n/a | n/a | n/a | unreadable | 0 |

Two microscopists reviewed thick and thin films from subject 2. Subjects 1 and 3 involved only one microscopists who reviewed both thick and thin films.

*microscopist did not specifically document the type of *P. vivax* parasites in each sample but simply commented there were “rings and trophozoites and a single gametocyte”. *P. vivax* parasite denominators can be found in supplementary material.

**Table 10. *P. vivax* microscopy findings for subject 4.**

| **Sample (HCT)** | **Reader 1 thick film** | **Reader 2 thick film** | **Reader 3 thin film** |
| --- | --- | --- | --- |
| **Pre-apheresis (46%)** | 7 rings | 6 trophozoites 2 female gametocytes | 1 trophozoite |
| **Intermediate (64%)** | 3 trophozoites | 6 trophozoites | n/a |
| **Final (3%)** | n/a | 4 trophozoites | n/a |
| **Spare (5%)** | n/a | n/a | n/a |
| **Waste (42%)** | 3 trophozoites | 2 rings 8 trophozoites | 1 trophozoite |

Two microscopists reviewed the thick films and one the thin films from subject 4. *P. vivax* parasite denominators can be found in supplementary material.


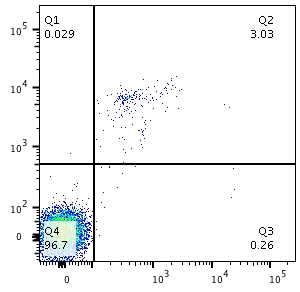
**Fig. 13 Apheresis single use cassette used during *P. vivax* apheresis clinical study**

**
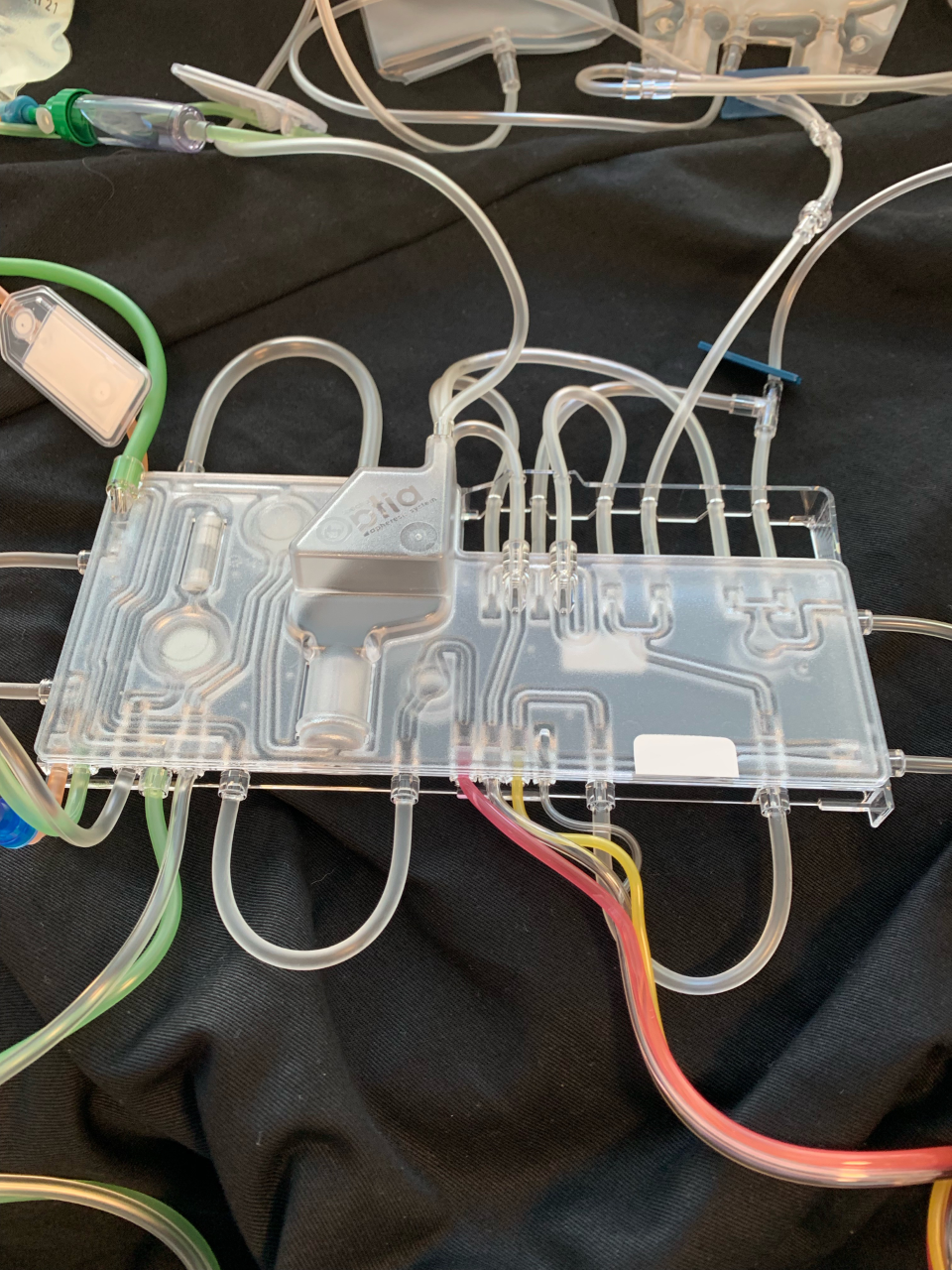
**

Magnet 2

Silo

Magnet 1

Example of an intermediate density layer apheresis single use casette used during apheresis. The 2 magnets and silo structure, where blood clots were found following subject 3’s apheresis procedure, are identified. Blood clots subsequently underwent 18S qPCR testing.

**Fig. 14** **Subject 3 additional testing *P. vivax* asexual parasites/ml**

Fold enrichment of *P. vivax* asexual parasites/ml compared to pre-apheresis, of blood clots around magnet 1, magnet 2, and silo in within the apheresis cassette and of post-centrifugation plasma originating from the collection bag. Asexual parasites were measured using 18S qPCR.

**Table 11. Subject 3 additional testing *P. vivax* asexual parasites/ml**

| **Sample** | **asexual parasites/ml** | **Fold enrichment asexual parasites/ml compared to pre-apheresis** |
| --- | --- | --- |
| **Pre-apheresis** | 25,475 | 1 |
| **0.5% post-centrifugation plasma** | 2,515 | 0.10 |
| **1% post-centrifugation plasma** | 1,168 | 0.05 |
| **2% post-centrifugation plasma** | 1,044 | 0.04 |
| **3% post-centrifugation plasma** | 3,345 | 0.13 |
| **5% post-centrifugation plasma** | 143 | 0.04 |
| **7% post-centrifugation plasma** | 2,300 | 0.09 |
| **11% post-centrifugation plasma** | 2,160 | 0.08 |
| **Apheresis cassette magnet 1** | 7,710 | 0.30 |
| **Apheresis cassette magnet 2** | 36,790 | 1.44 |
| **Apheresis cassette silo** | 31,401 | 1.23 |

Subject 3 additional testing *P. vivax* asexual parasites/ml and fold enrichment compared to pre-apheresis. Asexual parasites were measured using 18S qPCR.

**Table 12. Apheresis HMP bank if equivalent of HMP-013 *P. vivax* bank**

|  | **Number of parasite vials** |
| --- | --- |
| **Maximum** | 107 |
| **Required for Sterility (10%)** | 11 |
| ***Current stability testing** | 24 |
| ****Dose testing** | 9 |
| **Vials Remaining** | 63 |
| **Trials Possible of 1 patient** | 21 |

* Assuming a minimum of three years use under the current testing frequency of 8 tests per year

**3 vials required in triplicate

Table outlining the potential usage of a HMP bank produced by apheresis if that bank was equivalent in parasite concentration and volume to the HMP-013 *P. vivax* bank. Calculations based on experience from current HMP bank.

## **Human malaria parasite bank formation**

The maximum level of asexual parasite enrichment per ml of sample compared to pre-apheresis was 4.9-fold. Percoll concentration increases the theoretical level of enrichment to 15.5-fold compared to pre-apheresis.

The HMP-013 *P. vivax* parasite bank produces vials each of which contains 2.08 × 10^6^ parasites. The following formulas were used to calculate the viability of apheresis as a method to concentrate parasites. Maximum enrichment values were used as the purpose was to assess the possibility of producing a HMP bank using apheresis rather than the practicality.

Parasites/ml of enriched blood = Pre-apheresis whole blood 18S-qPCR/ml × Fold enrichment derived from percoll × Maximum fold enrichment from apheresis

Parasites/ml of packed blood (based on bank HCT of 0.39) = Parasites/ml of enriched blood/0.39

Number of parasites/vial compared based on 248 ul = (Parasites/ml of packed blood/1000) × 248

Parasites/ml of enriched blood = 25,475 × 3.17 × 4.89 = 3.95 × 10^5^

Parasites/ml of packed blood (based on bank HCT of 0.39) = 3.95 × 10^5^/0.39 = 1.01 × 10^6^

Number of parasites/vial based on 248ul = (1.01 × 10^6^/1000) × 248 = 2.51 × 10^5^

Hence apheresis and percoll cannot concentrate parasites enough to create HMP bank vials equivalent to those used to infect subjects in this study (2.51 × 10^5^ compared to 2.08 × 10^6^). If only apheresis is used to concentrate parasites the vials would contain a maximum of 7.92 × 10^4^ parasites.
