# Supplementary material for: Safety and feasibility of apheresis to harvest and concentrate parasites from subjects with induced blood stage Plasmodium vivax infection
Source: Malar J. 2021 Jan 14;20:43. doi: 10.1186/s12936-021-03581-w (PMC7807416; doi:10.1186/s12936-021-03581-w)
Supplement: Supplementary file 8 — Additional file 8. Apheresis Cohort 2 laboratory standard operating procedure. [file 12936_2021_3581_MOESM8_ESM.docx]

| Clinical Tropical Medicine, QIMR | | |
| --- | --- | --- |
| **CTM QIMR** | **Malarial Enrichment via Apheresis - Form** | Date Effective: |
| Version: | Status: | Review Date: Two years after review date |

**Apheresis laboratory master SOP: Cohort 2**

**Pre-processing**

1. Record all lot and catalogue numbers on QIMR- QF-75-B Malaria Parasite Isolation from Apheresis Product for volumes.
2. Spray or wipe labelled transfer bags, exchange couplers, CPDA-1 blood bags and necessary consumables into the hood using 80% v/v Ethanol.
3. Insert exchange coupler into CPDA-1 blood bag and aliquot citrate into labelled 50mL tubes.
4. Using an exchange coupler and pipette prefill labelled transferred bags with the correct citrate volumes.
   1. Refer to QIMR- QF-75-B Malaria Parasite Isolation from Apheresis Product for volumes.
5. Record times on QIMR-QF-75-B of when transfer bags containing citrate are prewarmed.

**Collection and Waterbath setup for maintaining temperature of transfer bags at Apheresis unit**

1. Refer to “Waterbath setup for blood bags”.
   1. Refer to and ensure to QIMR- QF-75-B Malaria Parasite Isolation from Apheresis Product for volumes is filled out for all fractions being collected.

**Transfer of transfer bags from Apheresis unit to CTM**

1. **“**Refer to Apheresis Thermos Blood Collection Method”.
2. Ensure “Apheresis_Thermos_Transport_Form” is filled out for each transfer of fraction of blood collected.

**Processing of Apheresis Blood Fractions**

1. **NB Ensure QIMR-QF-75-A Apheresis Fraction Processing is filled in .**
2. Ensure centrifuge is preheated using program 9 – 4500rpmr/HOLD/40°C.
3. Once blood bag has been transported to the waterbath in CTM and process is ready to continue, fill two large silver thermos’ with preheated water. Refer to “Appendix 1 – Table of Correct Water Volumes” for volume of water to add. Prepare one thermos for holding the blood bag and one thermos for holding 50ml falcons at temperature.
4. Check temperature of water and replace if below 38°C.
5. Place blood bag into thermos and move into BSC.
6. Move 50/250ml (for 1 and 2% HCT) collection tube into BSC and hold in thermos.
7. Use spike adaptor on sterile port of blood bag.
   1. **Note: Do not remove blood bag from thermos or lift above water level.**
8. Grab a 30/50ml preheated syringe from prewarming incubator.
9. Unscrew white cap of spike adaptor and place to the side.
10. Connect syringe to leur lock of spike adaptor.
11. Carefully syringe blood out of blood bag and dispense into 50ml/250ml collection tube.
12. Repeat process as necessary until all blood has been removed from blood bag.
13. Prepare a labelled 50ml falcon for sampling into a 50ml tube rack.
14. Mix collection tube well, either by gentle swirling or aspiration using a 25ml strippette.
15. Measure volume of HCT fraction received.
16. Aspirate an appropriate volume of sample into sampling tube. Sampling tube can be moved into another BSC for aliquoting during process.
    1. Refer to Master Sample list for details regarding subsampling
17. Spin 50ml/250ml collection tube in a preheated centrifuge at 1455g/5-15min/40°C
18. Replace water in thermos just before centrifuge stops spinning.
19. Collect collection tube from centrifuge with thermos and set centrifuge to preheat. (Program 9 – 4500rpm, HOLD, 40°C)
20. Place thermos back into BSC.
21. Use a strippette to remove plasma and place into a labelled collection tube.
22. Resuspend pellet and measure.
    1. For 1%, 2% or 3% HCT: Resuspend 650uL of pellet in 650uL of prewarmed AB serum for “Feed Mix”. Remainder is to be used for subsampling.
       - 1. Refer Master Sample list for details regarding subsampling.
       1. Prepare small thermos for transportation.
       2. Place FM into floatie and transport in small thermos to PC3.
       3. Conduct Membrane Feed as per CTM QIMR SOP 15 –Infection of Mosquitoes’ with plasmodium by membrane feeding
    2. For 5% and 7% HCT: Resuspend pellet in equal volume of RPMI and prepare thin and thick films.

**Appendix**

*Appendix 1*

Volume of Water to Add to Thermos:

1. Preeparing Cell pellets for storage prior to cryopreservation (per HCT Fraction)
   1. Transfer remaining pellet to prepared 25mL aliquots of pre-warmed (37°C) AB complete media.
   2. Add additional media until total volume is 30mL.
   3. Store at 37°C until shortly prior to cryopreservation.
   4. Record details on Apheresis Fraction Processing CTM QIMR QF 75A
2. Prechillng of cell solutions for cryopreservation (per HCT Fraction)
   1. Immediately before pre chilling solutions, prepare a dry ice/ethanol slurry in a plastic beaker
   2. Whilst rapidly mixing, prechill a dummy tube of WFI for a period of 50 seconds within slurry
   3. Measure temperature of dummy tube.
   4. If within 3-8°C, proceed with chilling of HCT Fraction cell suspension in the same manner as the dummy tube.
   5. If not alter slurry until desired specifications can be rechecked using fresh dummy tubes prior to proceeding with rapid chilling
   6. Wipe chilled cell suspension thoroughly with 80% v/v ethanol and transfer to ice slurry within clean biohazard hood.
   7. Evenly split cell suspension into two labelled tubes and transfer to fridge for storage at 2-8°C
   8. Record details on Apheresis Fraction Processing CTM QIMR QF 75A
3. Addition of Glycerolyte to RBCs/WBCS for Cryopreservation (per HCT Fraction)
   1. Calculate the total volume of glycerolyte needed (the pooled volume of the parasitised RBCs multiplied by 2.2).
   2. Record glycerolyte lot number expiry date and volume required on Section
   3. Centrifuge cell suspension at 530g/5-15min/ 2-8°C
   4. Remove and discard supernatant
   5. Agitate pellet (or gently pipette) to resuspend pellet.
   6. Slowly add dropwise 1/5 of the total volume of glycerolyte to the pooled volume RBCs, whilst gently shaking the tubes to mix the content.
   7. Note: Rapid introduction of glycerolyte can cause damage to RBCs. therefore glycerolyte should be added slowly and should be mixed well after addition.
   8. Incubate at room temperature (18-24°C) for 5 minutes.
   9. Add dropwise the remaining volume of glycerolyte.
   10. Add 1ml volumes to prechilled labelled nunc tubes.
   11. On completion of aliquting, ensure tubes are tightly sealed and transfer to a preconditioned Mr Frosty.
   12. Carefully transfer Mr Frosty to -80°C freezer for a minimum of twelve hours
   13. Record details on Apheresis Fraction Processing CTM QIMR QF 75A
4. Addition of Cryostor CS2 to WBC/RBC for Cryopreservation (per HCT Fraction)
   1. Remove a 50µL sample from the cell suspension tube stored at 2-8°C
   2. Perform total cell count (Refer to CTM QIMR SOP 38 – RBC Counting using a Haemocytometer) and record details of CTM QIMR QF 38A – RBC Calcualtion Form.
   3. Calculate the volume of Cryostor CS2 that the pellet would have to be resuspended in to give a concentration of 10^6^c/mL.
   4. Centrifuge cell suspension at 530g/5-15min/ 2-8°C
   5. Remove and discard supernatant
   6. Agitate pellet (or gently pipette) to resuspend pellet.
   7. Slowly add required volume of prechilled Cryostor CS2
   8. Add 1ml volumes to prechilled labelled nunc tubes.
   9. On completion of aliquting, ensure tubes are tightly sealed and transfer to a preconditioned Mr Frosty.
   10. Carefully transfer Mr Frosty to -80°C freezer for a minimum of twelve hours
   11. Record details on Apheresis Fraction Processing CTM QIMR QF 75A
