# Supplementary material for: Safety and feasibility of apheresis to harvest and concentrate parasites from subjects with induced blood stage Plasmodium vivax infection
Source: Malar J. 2021 Jan 14;20:43. doi: 10.1186/s12936-021-03581-w (PMC7807416; doi:10.1186/s12936-021-03581-w)
Supplement: Supplementary file 9 — Additional file 9. Apheresis Cohort 3 laboratory standard operating procedure. [file 12936_2021_3581_MOESM9_ESM.docx]

| Clinical Tropical Medicine, QIMR | | |
| --- | --- | --- |
| **CTM QIMR** | **Malarial Enrichment via Apheresis - Form** | Date Effective: |
| Version: | Status: | Review Date: Two years after review date |

**Pre-processing**

1. Record all lot and catalogue numbers on QIMR- QF-75-B Malaria Parasite Isolation from Apheresis Product for volumes.
2. Spray or wipe labelled transfer bags, exchange couplers, CPDA-1 blood bags and necessary consumables into the hood using 80% v/v Ethanol.
3. Insert exchange coupler into CPDA-1 blood bag and aliquot citrate into labelled 50mL tubes.
4. Using an exchange coupler and pipette prefill labelled transferred bags with the correct citrate volumes.
   1. Refer to QIMR- QF-75-B Malaria Parasite Isolation from Apheresis Product for volumes.
5. Record times on QIMR-QF-75-B of when transfer bags containing citrate are prewarmed.

**Processing of Apheresis Blood Fractions – Above 1%, 1%, 2%, 3%, 5% 7%, below 7% -20mL volumes**

1. **NB Ensure QIMR-QF-75-A Apheresis Fraction Processing is filled in .**
2. Ensure centrifuge is preheated using program 9 – 4500rpmr/HOLD/40°C.
3. Following wiping down with 80% v/v Ethanol, place blood bag into BSC.
4. Move 50mL collection tube into BSC.
5. Use spike adaptor on sterile port of blood bag.
6. Unscrew white cap of spike adaptor and place to the side.
7. Dispense fractions into 50ml collection tube.
8. Repeat process as necessary until all blood has been removed from blood bag.
9. Prepare a labelled 50ml falcon for sampling into a 50ml tube rack.
10. Mix collection tube well, either by gentle swirling or aspiration using a 25ml strippette.
11. Measure volume of HCT fraction received.
12. Aspirate an appropriate volume of sample into sampling tube. Sampling tube can be moved into another BSC for aliquoting during process.
    1. Refer to Master Sample list for details regarding subsampling
13. Spin 50ml collection tube in a preheated centrifuge at 1455g/5-15min/RT
14. Use a strippette to remove plasma and place into a labelled collection tube
    1. Refer to Master Sample list for details regarding subsampling and slide manufacture

**Pooling fractions for sampling– Above 1%, 1%, 2%, 3%, 5% 7%, below 7%: 2 mL volumes**

**2%,3% Pool – 5mL each, 5%,7% Pool – 5mL each**

1. From each of the fractions, sample the appropriate voilumes and pool into 50mL tubes
   - 1. Refer to Master Sample list for details regarding subsampling and slide manufacture
