# Supplementary material for: Safety and feasibility of apheresis to harvest and concentrate parasites from subjects with induced blood stage Plasmodium vivax infection
Source: Malar J. 2021 Jan 14;20:43. doi: 10.1186/s12936-021-03581-w (PMC7807416; doi:10.1186/s12936-021-03581-w)
Supplement: Supplementary file 10 — Additional file 10. Apheresis Cohort 4 laboratory standard operating procedure. [file 12936_2021_3581_MOESM10_ESM.docx]

| Clinical Tropical Medicine, QIMR | | |
| --- | --- | --- |
| **CTM QIMR** | **Malarial Enrichment via Apheresis - Form** | Date Effective: |
| Version: | Status: | Review Date: Two years after review date |

**Apheresis laboratory master SOP: Cohort 4**

**EXPERIMENTAL PLAN**

**APHERESIS DAY 1**

**TIME POINTS AND TASKS**

**PRE-APHERESIS T=0hr**

- Blood collection (Q-Pharm; 1 x 6 mL EDTA, 2 x 10mL LiHep tubes)
- Pre-aliquot 19,600 µL and 39,200 µL of McCoy media into 12 x PRE-APHERESIS T75 (T=8hr, T=16hr, T=32hr, T=40hr and T=48hr (=/- NAG) and 3 x PRE-APHERESIS T225 flasks (T=4hr, T=24hr, T=48hr) respectively.
- 1 x 6mL EDTA TUBE
  - Aliquot samples for testing (18S SAF, pvs25, Sysmex)
  - Aliquot samples for and prepare thick/thin films
  - Aliquot sample for FLOW cytometry, laser capture microdissection slide (LCS)
- 1 x 10mL LIHEP TUBE:
  - Prepare feed mix and perform MFA
  - Set up 3 x T225 MFA culture flasks (T=4hr, T=24hr, T=48hr)
- 1x10mL LiHEP TUBE:
  - Set up 12 x T75 asexual/sexual culture flasks (+/- NAG) (T=8hr, T=16hr, T=24hr, T=32hr, T=40hr, T=48hr)
  - Aliquot sample for pLDH

**INTERMEDIATE T=0hr, FINAL T=0hr, SPARE T=0hr (*optional*), WASTE T=0hr**

- Intermediate, Final, Spare (*optional*)and Waste sample collection (Apheresis Unit)
- Aliquot samples for testing (18S SAF, pvs25, Sysmex)
- Aliquot samples for and prepare thick/thin films
- Aliquot sample for FLOW cytometry
- Prepare feed mix and perform MFA
- Set up 3 x T225 MFA cultures (T=4hr, T=24hr, T=48hr)
- Set up 4 x T75 Asexual/Sexual cultures (+/- NAG) (T=24hr, T=48hr)
- Aliquot sample for pLDH

**PRE-APHERESIS T=4hr**

- Prepare feed mix from 1 x T=4hr T225 flask and perform MFA
- Aliquot sample for and prepare thin films from T=4hr T225 flask
- Aliquot sample for DNA, RNA, FLOW cytometry from T=4hr T225 flask

**INTERMEDIATE T=4hr**

- Prepare feed mix from 1 x T=4hr T225 flask and perform MFA
- Aliquot sample for and prepare thin film from T=4hr T225 flask
- Aliquot sample for DNA, RNA, FLOW cytometry from T=4hr T225 flask

**PRE-APHERESIS T=8hr**

- Aliquot samples for pLDH from 2 x T=8hr T75 flasks (+/- NAG)
- Aliquot samples for and prepare thin film from 2 x T=8hr T75 flasks (+/- NAG)
- Aliquot samples for DNA, RNA, FLOW cytometry from 2 x T=8hr T75 flasks (+/- NAG)

**PRE-APHERESIS T=16hr**

- Aliquot samples for pLDH from 2 xT=16hr T75 flasks (+/- NAG)
- Aliquot sample for and prepare thin films from 2x T=16hr T75 flasks (+/- NAG)
- Aliquot sample for DNA, RNA, FLOW cytometry from 2 x T=16hr T75 flasks (+/- NAG)

**APHERESIS DAY 2**

**TIME POINTS AND TASKS**

**PRE-APHERESIS T=24hr; INTERMEDIATE T=24hr; FINAL T=24hr; WASTE T=24hr**

**For each time point sample type:**

- Prepare feed mix from T=24hr T225 flask and perform MFA
- Aliquot sample for and prepare thin film from T=24hr T225 flask
- Aliquot sample for DNA, RNA from T=24hr T225 flask
- Aliquot sample for pLDH from 2 x T=24hr T75 flasks (+/- NAG)
- Aliquot sample for and prepare thin films from 2 x T=24hr T75 flasks (+/- NAG)
- Aliquot sample for DNA, RNA, FLOW cytometry from 2 x T=24hr T75 flasks (+/- NAG)

**PRE-APHERESIS T=32hr**

- Aliquot sample for pLDH from 2 x T=32hr T75 flasks (+/- NAG)
- Aliquot sample for and prepare thin film from 2 x T=32hr T75 flasks (+/- NAG)
- Aliquot sample for DNA, RNA, FLOW cytometry from 2 x T=32hr T75 flasks (+/- NAG)

**PRE-APHERESIS T=40hr**

- Aliquot sample for pLDH from 2 x T=40hr T75 flasks (+/- NAG)
- Aliquot sample for and prepare thin film from 2 x T=40hr T75 flasks (+/- NAG)
- Aliquot sample for DNA, RNA, FLOW cytometry from 2 x T=40hr T75 flasks (+/- NAG)

**MEDIA CHANGES**

- Perform 50% media change on remaining T=48hr T225 flasks
- Perform 50% media change on remaining T=32hr; T=40hr; T=48hr T75 flasks

**APHERESIS DAY 3**

**TIME POINTS AND TASKS**

**PRE-APHERESIS T=48hr; INTERMEDIATE T=48hr; FINAL T=24hr;** **SPARE (*optional*) T=24hr; WASTE T=24hr**

**For each time point sample type:**

- Prepare feed mix from T=48hr T225 flask and perform MFA
- Aliquot sample for and prepare thin/thick films from T=48hr T225 flask
- Aliquot sample for DNA, RNA, FLOW cytometry from T=48hr T225 flask
- Aliquot sample for pLDH from 2 x T=48hr T75 flasks (+/- NAG)
- Aliquot sample for and prepare thin film from 2 x T=48hr T75 flasks (+/- NAG)
- Aliquot sample for DNA, RNA, FLOW cytometry from 2 x T=48hr T75 flask (+/- NAG)

**PROCEDURE**

**APHERESIS DAY 1**

**PRE-APHERESIS T=0hr BLOOD COLLECTION** (Q-Pharm; **1 x 6mL EDTA tube, 2 x10mL LiHep tubes**)

**PRE-BLOOD COLLECTION PROCESS**

1. Turn on water baths in the CTM general lab (set 38.5°C) and PC3 (set 40°C).
2. Turn on heat blocks in TC (set 40°C).
3. Configure Mediheat unit for sample transport.
4. Collect apheresis sampling labels, Mediheat unit, esky, APH kit and take over to Apheresis Ward.
5. Once at the ward, set up the Mediheat unit as per SOP and label collection bags.
6. Return to CTM lab and prepare for pre-apheresis blood sample transport from Q-Pharm.
7. Clean BSC with 80% ethanol.
8. Pre-aliquot 19,600 μL and 39,200 μL of McCoy media into pre-labelled 12 x PRE-APHERESIS T75 (T=8hr, T=16hr, T=32hr, T=40hr and T=48hr (+/- NAG) and 3 x PRE-APHERESIS T225 flasks (T=4hr, T=24hr, T=48hr) respectively.
9. Following phone call from Q-Pharm, prepare for blood collection.
10. Fill required number of thermos flasks with a pre-measured volume (650mL) of pre-heated water. Ensure a spare thermos flask is also filled.
11. Place no more than 2 thermos flasks per esky. Bring a spare esky for collection of blood tubes and beakers.
12. Head to Q-Pharm, Level 5 CBCRC and gain entry from reception. Collect pre-heated blood tubes and beakers. Transport to Level 5 CBCRC.
13. Place a maximum of 4 x 10mL tubes per thermos flask. Place a maximum of 5 x 6mL tubes per thermos flask.
14. ***Note: Tubes will need to be inverted upon collection from Q-Pharm staff. Invert tubes 3-4 times.***
15. **Record blood draw and collection time on transport form** QIMRB CTM QF-15-C.
16. Transport tubes to Tissue Culture lab on level 13 Bancroft and place blood tubes in waterbath set to 38.5°C.

**ALIQUOT PRE-APHERESIS T=0hr SAMPLES FROM EDTA TUBE**

1. Wipe blood tube into BSC with 80% ethanol.
2. Invert blood collection tubes to mix, then transfer the contents of the EDTA blood collection tube into a 50mL tube and the contents of the two LiHep blood collection tubes into two separate 50mL tubes.
3. Aliquot samples for **18S, pvs25, Sysmex, thin/thick films, laser capture (LCS) and FLOW** from EDTA 50 mL tube according to **Table 1**, mixing well between aliquots. **Record, Initial and date.**

| **Sample Source** | **Sample Type** | **No. Aliquots** | **Sample**  **Volume (µL)** | **Test** | **Buffer Vol. (µL)** | **Initial & Date** |
| --- | --- | --- | --- | --- | --- | --- |
| Pre-Apheresis | QPID | **3** | **250** | 18S_SAF | 400 |  |
| Pre-Apheresis | QPID | **3** | **250** | pvs25 | 1250 |  |
| Pre-Apheresis | Sysmex | **1** | **1000** | Blood count | - |  |
| Pre-Apheresis | Thin Film | **1** | **50** | Parasitemia | - |  |
| Pre-Apheresis | Thick Film | **1** | **50** | Parasitemia | - |  |
| Pre-Apheresis | Flow | **1** | **500** | Parasitemia | - |  |
| Pre-Apheresis | LCS | **1** | **1500** | Molecular | - |  |

1. Store samples as per **Table 2**. **Record time stored, initial/date.**
2. Prepare 3 x thick and 3 x thin blood films from EDTA sub-sample tube. Refer to SOP-MBE-1.
3. Store films as per **Table 2**. **Record time stored, initial/date.**
4. Prepare blood films for LCS according to Experimental Plan (GR).

| **Table 2. PRE-APHERESIS T=0hr Sample Storage** | | | | | | |
| --- | --- | --- | --- | --- | --- | --- |
| **Sample Source** | **Sample Type** | **Storage condition** | **Storage location** | **Storage location details (e.g. KT freezer, Shelf 1, Box 1)** | **Time stored** | **Initial & Date** |
| Pre-Apheresis | QPID | -80°C | -80°C freezer |  |  |  |
| Pre-Apheresis | QPID | -80°C | -80°C freezer |  |  |  |
| Pre-Apheresis | Sysmex | 4-8°C | TC blood fridge |  |  |  |
| Pre-Apheresis | Flow | 4-8°C | Flow room fridge |  |  |  |
| Pre-Apheresis | Thin Films | Ambient | Labelled Slide Box |  |  |  |
| Pre-Apheresis | Thick Films | Ambient | Labelled Slide Box |  |  |  |
| Pre-Apheresis | LCS | Ambient | Labelled Slide Box |  |  |  |

**PRE-APHERESIS T=0hr MEMBRAME FEED ASSAY (MFA) ~ 9:30AM**

**Note:** Keep sample heated throughout process.

1. Centrifuge 1 x 50mL LiHep tube at 530xg for 5 mins at 40°C in a pre-heated centrifuge.
2. While sample is spinning, aliquot 1.3mLof AB serum into labelled 2mL eppendorf tube.
3. Remove supernatant from sample and discard.
4. Aliquot 650µl of packed infected red blood cells (RBCs) into eppendorf tube and mix.
5. Fill small thermos flask with pre-heated water.
6. Place feed mix tube in blue floaty and transport to PC3 in thermos flask.
7. Perform MFA as per CTM QIMR SOP 15.

**Day 1 NF54 CONTROL MFA**

**Note:** Prepare NF54 feed-mix at the same time as PRE-APHERESIS MFA process above.

1. Remove 15mL of culture from gametocyte plate into pre-warmed 50mL tube.
2. Centrifuge at 530xg for 5 mins at 40°C in a pre-warmed centrifuge.
3. Aliquot 1.3mL of AB serum into a 2mL Eppendorf tube.
4. Fill small thermos flask to 1/2 - 2/3 total volume with pre-heated water.
5. Once sample has been centrifuged, remove supernatant.
6. Add 650µL of packed infected red blood cells into 2mL tube and mix well.
7. Place 2mL eppendorf tube into blue floaty and then into small thermos flask.

**PRE-APHERESIS T=0hr T225 MFA CULTURE SET-UP**

1. After the feed-mix is prepared, set up 3 x Pre-Apheresis T225 MFA Culture flasks (T=4hr, T=24hr and T=48hr).
2. Mix the RBC pellet in the same 50mL tube used to prepare the feed mix and transfer 800µL packed RBCs into each of the T225 flasks (Pre-Apheresis T225 MFA Culture flask T=4hr, T=24hr and T=48hr).
3. Gas each flask for 1 min at ≥ 10L/min.
4. Wipe flasks with 80% ethanol before placing in the incubator.
5. **Place the T=48hr flask in the vertical position.**
6. **Record the volume of packed red cells and the actual culture set-up time/date in Table 3, Appendix I.**

**PRE-APHERESIS T=0hr T75 ASEXUAL/SEXUAL PARASITE CULTURE SET-UP**

1. Set up 12 x PRE-APHERESIS T75 (T=8hr, T=16hr, T=32hr, T=40hr and T=48hr (+/- NAG).
2. Centrifuge the second 50mL LiHep tube at 530xg for 5 mins at 40°C in a pre-heated centrifuge.
3. Remove supernatant from sample and discard.
4. Mix pellet and transfer 400µL of packed red blood cells into each of the twelve T75 flasks pre-filled with media (T=8hr, T=16hr, T=24hr, T=32hr,T=40hr, T=48hr +/- NAG).
5. Gas each flask for 30sec at 7-10L/min..
6. Wipe flasks with 80% ethanol before placing in the incubator.
7. **Place T=32hr, T=40hr and T=48hr flask +/- NAG in vertical position** (6 T75 flasks in total).
8. **Record the volume of packed red cells and the actual culture set-up time/date in Table 4, Appendix I.**
9. Aliquot sample for pLDH. **Record in Table 4, Appendix I.**

**INTERMEDIATE T=0hr SUB-SAMPLE COLLECTION (Apheresis Ward)**

***Note: A Red Cell Depletion process will be run by the Apheresis Nurse on an apheresis unit (RBWH; Apheresis Ward). A sub-sample (INTERMEDIATE sample; 20-25mL) will be harvested from the collection bag. Lab staff from CTM Bancroft 13 lab will be called to retrieve the sample.***

1. Using pre-heated water, fill a thermos flask of appropriate size with appropriate insert and place in an esky. Transport the esky to the Apheresis ward.
2. Transport the INTERMEDIATE sub-sample to CTM Bancroft Level 13 lab in the thermos flask within the esky.

***Note: Citrate may be sterilely injected into bag after process if concerns of clotting arise.* The Apheresis Nurse will transfer Red Blood Cell Depletion to a Bone Marrow Processing accessory set for the PMN apheresis process (i.e for collection of FINAL AND WASTE samples).**

**DETERMINE SAMPLE VOLUME AND THE HAEMATOCRIT OF THE INTERMEDIATE T=0hr SUB-SAMPLE**

1. Following wiping down with 80% Ethanol, place sample into BSC.
2. Prepare a labelled 50mL falcon tube for sampling into a 50mL tube rack.
3. Move labelled 50mL collection tube into BSC.
4. Mix sample tube well, either by gentle swirling or aspiration using a strippette.
5. Measure volume of sample received using a strippette and **record in Table 5.**
6. Dispense the sample aseptically into 50mL collection tube.

| **Table 5. Sample volume received following apheresis process** | | | | |
| --- | --- | --- | --- | --- |
| **Sample Source** | **Time Received** | **Total volume of sample (mL)** | **Date** | **Initials** |
| Intermediate |  |  |  |  |
| Final |  |  |  |  |
| Spare |  |  |  |  |
| Waste |  |  |  |  |

1. Mix collection tube well, either by gentle swirling or aspiration using a strippette.
2. Aliquot 100µL of sample into 1.5mL eppendorf tube for Coulter Counter sampling.
3. Perform Coulter Counter analysis on Level 9 CBCRC.
4. Once haematocrit is determined, contact either Anand (0411 041 391) or Kari (0438 736 371) to inform of the result.

**ALIQUOT INTERMEDIATE T=0hr SAMPLES FOR TESTING**

1. Aliquot samples for **18S, pvs25, Sysmex, thin/thick films, FLOW cytometry, MFA/T225 MFA culture set up, T75 Asexual/Sexual culture set up** according to **Table 6**, mixing well between aliquots. **Record Initial and date.**

| **Sample Source** | **Sample Type** | **No. Aliquots** | **Sample Volume (µL)** | **Test** | **Buffer Volume (µL)** | **Initial & Date** |
| --- | --- | --- | --- | --- | --- | --- |
| Intermediate | QPID | **3** | **250** | 18S_SAF | 400 |  |
| Intermediate | QPID | **3** | **250** | pvs25 | 1250 |  |
| Intermediate | Sysmex | **1** | **1000** | Blood count | - |  |
| Intermediate | Thin Film | **1** | **50** | Parasitemia | - |  |
| Intermediate | Thick Film | **1** | **50** | Parasitemia | - |  |
| Intermediate | Flow | **1** | **500** | Parasitemia | - |  |
| Intermediate | Asexual/Sexual Culture | **1** | **10,000** | Growth | - |  |
| Intermediate | MFA | **1** | **10,000** | Transmission | - |  |

1. Store samples as per **Table 7**.
2. Prepare 3 x thick and 3 x thin blood films. Refer to SOP-MBE-1.
3. Store blood films as per **Table 7**.

| **Table 7. INTERMEDIATE T=0hr Sample Storage** | | | | | |
| --- | --- | --- | --- | --- | --- |
| **Sample Source** | **Sample Type** | **Storage condition** | **Storage location** | **Time stored** | **Initial & Date** |
| Intermediate | QPID | -80°C | -80°C freezer |  |  |
| Intermediate | QPID | -80°C | -80°C freezer |  |  |
| Intermediate | Sysmex | 4-8°C | TC blood fridge |  |  |
| Intermediate | Flow | 4-8°C | Flow room fridge |  |  |
| Intermediate | Thin Films | Ambient | Labelled Slide Box |  |  |
| Intermediate | Thick FIlms | Ambient | Labelled Slide Box |  |  |

**INTERMEDIATE T=0hr MFA ~ 12:00PM**

***Note:*** *Keep sample heated throughout process.*

1. Centrifuge 10mL of INTERMEDIATE sub-sample (in 50mL tube) at 530xg for 5 mins at 40°C in a pre-heated centrifuge.
2. While sample is spinning, aliquot 1.3mLof AB serum into labelled 2mL eppendorf tube.
3. Remove supernatant from sample and discard.
4. Aliquot 650µl of packed infected red blood cells into eppendorf tube and mix.
5. Fill small thermos flask with pre-heated water.
6. Place feed mix tube in blue floaty and transport to PC3 in thermos flask.
7. Perform MFA as per CTM QIMR SOP 15.

**INTERMEDIATE T=0hr T225 MFA CULTURE SET-UP**

1. After the feed-mix is prepared, aliquot 39,200 µL McCoy media into 3 x INTERMEDIATE T225 MFA Culture flasks (T=4hr, T=24hr and T=48hr).
2. Mix the RBC pellet in the same 50mL tube used to prepare the feed mix and transfer 800µL packed RBCs into each of the 3 x INTERMEDIATE T225 flasks (T=4hr, T=24hr and T=48hr).
3. Gas each flask for 1 min at ≥ 10L/min.
4. Wipe flasks with 80% ethanol before placing in the incubator.
5. **Place the T=48hr flask in the vertical position.**
6. **Record the volume of packed red cells and the actual culture set-up time/date in Table 3, Appendix I.**

**INTERMEDIATE T=0hr T75 ASEXUAL/SEXUAL PARASITE CULTURE SET-UP**

1. Aliquot 19,600 µL McCoy media (+/- NAG) into 4 x INTERMEDIATE T75 flasks (T=24hr, T=48hr +/- NAG).
2. Centrifuge 10mL of INTERMEDIATE sub-sample at 530xg for 5 mins at 40°C in a pre-heated centrifuge.
3. Remove supernatant from sample and discard.
4. Mix pellet and transfer 400µL of packed red blood cells into 4 xT75 flasks pre-filled with media (T=24hr, T=48hr +/- NAG).
5. Gas each flask for 30 sec at 7-10L/min..
6. Wipe flasks with 80% ethanol before placing in the incubator.
7. **Record the volume of packed RBCs and the actual culture set-up time/date in Table 4, Appendix I.**
8. **Place the T=48hr flasks (+/- NAG) in the vertical position** (2 T75 flasks in total).
9. Aliquot sample for pLDH. **Record in Table 4, Appendix I.**

**PRE-APHERESIS T=4hr MFA AND SAMPLING (T225 FLASK) ~ 1:30PM**

***Note:*** *One hour prior to preparing feed mix, pre-warm the centrifuge, thaw an aliquot of AB serum, rack up pre-labelled tubes for sampling (DNA, RNA, pLDH, FLOW cytometry) and have slides available for preparing blood films in the work station.*

1. Remove the PRE-APHERESIS T225 T=4hr flask from incubator.
2. Wipe all the surfaces with 80% ethanol.
3. Transfer the entire contents of the flask into a 50 mL tube.
4. Centrifuge at 530xg for 5 mins at 40°C in a pre-warmed centrifuge.
5. While sample is spinning, aliquot 1.3mL of AB serum into labelled 2mL eppendorf tube.
6. Remove 90% of the supernatant from sample and discard.
7. Aliquot 650µl of packed infected red blood cells into eppendorf tube and mix.
8. Fill a small thermos flask with pre-heated water.
9. Place feed mix tube in blue floaty and transport to PC3 in thermos flask.
10. Perform membrane feed assay as per CTM QIMR SOP 15.
11. Aliquot the remaining packed RBC transferring 100µL in DNA tubes (containing 400 µL lysis buffer) and 100µL in RNA tubes (containing 1000 µL RNACell protect) and 50µL for FLOW cytometry.
12. Store samples as per **Table 12, Appendix I**. **Record volume, initial date.**
13. Aliquot 10µL for preparing thin blood film.
14. Prepare thin blood film. **Store blood film as per Table 12, Appendix I.**
15. **Record pRBC volume, actual date and time in Table 12, Appendix I.**

**FINAL, SPARE *(optional)* & WASTE T=0hr SAMPLE COLLECTION (Apheresis Ward)**

1. Label BMP accessories set bag with “WASTE” label, ensure label is over existing label.
2. Place collection bags in Mediheat transport unit.

***Note:*** *While collecting sample, make sure to frequently mix the bag. The Apheresis Nurse will run PMN collection process on apheresis machine and collect 100mL of first fraction based on collection colour into a bag labelled “FINAL”. A decision will be made to collect a further 100mL if possible, into a bag labelled “SPARE”. Bags will be heat sealed prior to removal from Apheresis Ward.*

1. Place all bags including BMP accessories set/WASTE bag in Mediheat for transport to CTM Bancroft Level 13.

**FINAL**, **SPARE *(optional)* & WASTE T=0hr SUB-SAMPLING FOR TESTING**

1. Following wiping down with 80% v/v Ethanol, place bags into BSC.
2. Prepare a labelled 50mL falcon tubes for sampling into a 50mL tube rack.
3. Move labelled 50mL tubes into BSC.
4. Mix sample bag well.
5. Dispense sample aseptically into 250mL tube using a sterile spike adaptor.
6. Measure volume of sample received using a strippette/pipette and record in **Table 8.**

| **Table 8. Sample volume received following apheresis process** | | | | |
| --- | --- | --- | --- | --- |
| **Sample Source** | **Time Received** | **Total volume of sample (mL)** | **Date** | **Initials** |
| Intermediate |  |  |  |  |
| Final |  |  |  |  |
| Spare |  |  |  |  |
| Waste |  |  |  |  |

1. For each sample source, aliquot samples for **18S, pvs25, Sysmex, thin/thick films, FLOW cytometry, MFA/T225 MFA culture set up, T75 Asexual/Sexual culture set up according to Table 9**, mixing well between aliquots. **Initial and date.**

| **Table 9. FINAL BAG, SPARE BAG (optional) and WASTE BAG T=0hr Sampling** | | | | | | |
| --- | --- | --- | --- | --- | --- | --- |
| **Sample Source** | **Sample Type** | **No. Aliquots** | **Sample Volume (µL)** | **Test** | **Buffer Volume (µL)** | **Initial & Date** |
| Final | QPID | **3** | **250** | 18S_SAF | 400 |  |
| Final | QPID | **3** | **250** | pvs25 | 1250 |  |
| Final | Sysmex | **1** | **1000** | Blood count | - |  |
| Final | Thin Film | **1** | **50** | Parasitemia | - |  |
| Final | Thick Film | **1** | **50** | Parasitemia | - |  |
| Final | Flow | **1** | **500** | Parasitaemia | - |  |
| Final | T75 Asexual/Sexual Culture | **1** | **10,000** | Growth | - |  |
| Final | MFA/T225 MFA Culture | **1** | **10,000** | Transmission | - |  |
| Spare | QPID | **3** | **250** | 18S_SAF | 400 |  |
| Spare | QPID | **3** | **250** | pvs25 | 1250 |  |
| Spare | Sysmex | **1** | **1000** | Blood count | - |  |
| Spare | Thin Film | **1** | **50** | Parasitemia | - |  |
| Spare | Thick Film | **1** | **50** | Parasitemia | - |  |
| Spare | Flow | **1** | **500** | Parasitaemia | - |  |
| Waste | QPID | **3** | **250** | 18S_SAF | 400 |  |
| Waste | QPID | **3** | **250** | pvs25 | 1250 |  |
| Waste | Sysmex | **1** | **1000** | Blood count | - |  |
| Waste | Thin Film | **1** | **50** | Parasitemia | - |  |
| Waste | Thick Film | **1** | **50** | Parasitemia | - |  |
| Waste | Flow | **1** | **500** | Parasitaemia | - |  |
| Waste | T75 Asexual/Sexual Culture | **1** | **10,000** | Growth | - |  |
| Waste | MFA/T225 MFA Culture | **1** | **10,000** | Transmission | - |  |

1. Store samples as per **Table 10**.
2. For each sample source, prepare 3 x thick and 3 x thin blood films. Refer to SOP-MBE-1.
3. **Store films as per Table 10. Record time stored, initial/date.**

| **Table 10. FINAL,**  **SPARE (optional) AND WASTE T=0hr SAMPLE STORAGE** | | | | | |
| --- | --- | --- | --- | --- | --- |
| **Sample Source** | **Sample Type** | **Storage condition** | **Storage location** | **Time stored** | **Initial & Date** |
| **FINAL** | | | | | |
| Final | QPID 18S_SAF | -80°C | -80°C freezer |  |  |
| Final | QPID pvs25 | -80°C | -80°C freezer |  |  |
| Final | Sysmex | 4-8°C | TC blood fridge |  |  |
| Final | Flow | 4-8°C | Flow room fridge |  |  |
| Final | Thin Film | Ambient | Labelled Slide Box |  |  |
| Final | Thick Film | Ambient | Labelled Slide Box |  |  |
| **WASTE** | | | | | |
| Waste | QPID 18S_SAF | -80°C | -80°C freezer |  |  |
| Waste | QPID pvs25 |  |  |  |  |
| Waste | Sysmex | 4-8°C | TC blood fridge |  |  |
| Waste | Flow | 4-8°C | Flow room fridge |  |  |
| Waste | Thin Film | Ambient | Labelled Slide Box |  |  |
| Waste | Thick Film | Ambient | Labelled Slide Box |  |  |
| ***OPTIONAL SPARE*** | | | | | |
| Spare | QPID 18S_SAF | -80°C | -80°C freezer |  |  |
| Spare | QPID pvs25 | -80°C | -80°C freezer |  |  |
| Spare | Sysmex | 4-8°C | TC blood fridge |  |  |
| Spare | Flow | 4-8°C | Flow room fridge |  |  |
| Spare | Thin Film | Ambient | Labelled Slide Box |  |  |
| Spare | Thick Film | Ambient | Labelled Slide Box |  |  |

**FINAL & WASTE T=0hr MFA ~ 2:00PM**

***Note****: Keep sample heated throughout process.*

1. Centrifuge 10mL of FINAL, SPARE (optional) and WASTE sub-sample at 530xg for 5 mins at 40°C in a pre-heated centrifuge.
2. While sample is spinning, aliquot 1.3mL of AB serum into labelled 2mL eppendorf tube.
3. Remove supernatant from sample and discard.
4. Aliquot 650µl of packed infected RBCs into eppendorf tubes and mix.
5. Fill small thermos flask with pre-heated water.
6. Place feed mix tubes in blue floaty and transport to PC3 in thermos flask.
7. Perform MFA as per CTM QIMR SOP 15.

**FINAL & WASTE T=0hr T225 MFA CULTURE SET-UP**

1. After the feed-mix is prepared, aliquot 39,200 µL McCoy media into 4 x FINAL and WASTE T225 MFA Culture flasks (T=24hr and T=48hr).
2. Mix the RBC pellet in the same 50mL tube used to prepare the feed mix and transfer 800µL packed RBCs into each of the T225 flasks (FINAL and WASTE T225 MFA Culture flask T=24hr and T=48hr).
3. Gas each flask for 1 min at 10L/min.
4. Wipe flasks with 80% ethanol before placing in the incubator.
5. **Place the T=48hr flask in the vertical position.**
6. **Record the volume of packed red cells and the actual culture set-up time/date in Table 3, Appendix I.**

**FINAL, SPARE (optional) & WASTE T=0hr T75 ASEXUAL/SEXUAL PARASITE CULTURE SET-UP**

1. Aliquot 19,600 µL McCoy media (+/- NAG) into 8 x FINAL and WASTE T75 Asexual/Sexual Culture flasks (T=24hr, T=48hr +/- NAG).
2. Centrifuge 10mL of FINAL and WASTE sub-sample and centrifuge at 530xg for 5 mins at 40°C in a pre-heated centrifuge.
3. Remove supernatant from sample and discard.
4. Mix pellet and transfer 400µL of packed red blood cells into each of the 8 x T75 flasks pre-filled with media (T=24hr, T=48hr +/- NAG).
5. Gas each flask for 30 sec at 7-10L/min.
6. Wipe flasks with 80% ethanol before placing in the incubator.
7. **Record the volume of packed RBCs and the actual culture set-up time/date in Table 4, Appendix I.**
8. **Place the T=48hr flasks (+/- NAG) in the vertical position (2 T75 flasks in total).**
9. Aliquot sample for pLDH**. Record in Table 4, Appendix I.**

**DELIVER SYSMEX SAMPLES TO PATHOLOGY QUEENSLAND**

1. Once all samples are collected for SYSMEX analysis, place relevant samples (with completed sample request form) in a bag in an esky and deliver to Pathology QLD no later than 4:00pm.

**INTERMEDIATE T=4hr MFA (T225 FLASK) ~ 4:00PM**

***Note:*** *One hour prior to preparing feed mix, pre-warm the centrifuge, thaw an aliquot of AB serum, rack up pre-labelled tubes for sampling (DNA, RNA, pLDH, FLOW cytometry) and have slides available for preparing blood films in the work station.*

1. Remove the INTERMEDIATE T225 T=4hr MFA flask from incubator.
2. Wipe all the surfaces with 80% ethanol.
3. Transfer the entire contents of the flask into a 50 mL tube.
4. Centrifuge at 530xg for 5 mins at 40°C in a pre-warmed centrifuge.
5. While sample is spinning, aliquot 1.3mL of AB serum into labelled 2mL eppendorf tube.
6. Remove 90% of the supernatant from sample and discard.
7. Aliquot 650µl of packed infected red blood cells into eppendorf tube and mix.
8. Fill a small thermos flask with pre-heated water.
9. Place feed mix tube in blue floaty and transport to PC3 in thermos flask.
10. Perform membrane feed assay as per CTM QIMR SOP 15.
11. Aliquot the remaining packed RBC transferring 100µL in DNA tubes (containing 400 µL lysis buffer) and 100µL in RNA tubes (containing 1000 µL RNAcell protect). Record in **Table 12, Appendix I.**
12. Store samples as per **Table 12, Appendix I.**
13. Aliquot 10µL for preparing blood films.
14. Prepare thin blood slide. **Store blood films as per Table 12. Record pRBC volume, actual date and time in Table 12, Appendix I.**

**PRE-APHERESIS T=8hr T75 SAMPLING ~ 5:30PM**

**Note:** Sampling will be performed every 8 hours after culture set up (T=8hr, T=16hr, T=24hr, T=32hr, T=40hr and T=48hr). **Table 13** will be generated with actual times to harvest at the end of APHERESIS DAY 1 after all samples have been received.

1. Ten minutes before the set-up time, prepare/rack up sampling tubes (DNA, RNA, Flow and pLDH) and have slides available for preparing blood films in the work station.
2. Verify time-point and the type of media in the label of the 2 X T75 flasks.
3. Remove the 2 x PRE-APHERESIS T=8hr T75 flasks (+/- NAG) from incubator.
4. Wipe all the surfaces with 80% ethanol.
5. Transfer the entire contents of each flask into a pre-labeled 50mL tube.
6. Centrifuge tubes at 530xg for 5 mins at 40°C and remove and discard 90% of supernatant
7. For each flask, aliquot packed RBCs transferring 150µL in pLDH tubes (O-Ring tubes empty) 100µL in DNA tubes (containing 400 µL lysis buffer) and 100µL in RNA tubes (containing 1000 µL RNAcell protect) and 50µL for FLOW cytometry**. Record in Table 13, Appendix I.**
8. Store samples as per **Table 13, Appendix I.**
9. Aliquot 10µL for preparing blood films.
10. Prepare 2 x thin blood films. **Store blood films as per Table 13, Appendix I.**
11. **Record pRBC volume, actual date and time in Table 13, Appendix I.**

**PRE-APHERESIS T=16hr T75 SAMPLING ~ 1:30AM**

1. Ten minutes before the set-up time, prepare/rack up sampling tubes (DNA, RNA, flow and pLDH) and have slides available for preparing blood films in the work station.
2. Verify time-point and the type of media in the label of the 2 X T75 flasks.
3. Remove the 2 x PRE-APHERESIS T=16hr T75 flasks (+/- NAG) from incubator.
4. Wipe all the surfaces with 80% ethanol.
5. Transfer the entire contents of each flask into a pre-labeled 50mL tube.
6. Centrifuge tubes at 530xg for 5 mins at 40°C and remove and discard 90% of supernatant
7. For each flask, aliquot packed RBCs transferring 150µL in pLDH tubes (O-Ring tubes empty) 100µL in DNA tubes (containing 400 µL lysis buffer) and 100µL in RNA tubes (containing 1000 µL RNAcell protect) and 50µL for FLOW cytometry. **Record in Table 13, Appendix I.**
8. Store samples as per **Table 13, Appendix I.**
9. Aliquot 10µL for preparing blood films.
10. Prepare 2 x thin blood films. **Store blood films as per Table 13, Appendix I.**

**APPENDIX I**

**TABLES**

| **Table 3. T225 MFA Culture Set-up Times** | | | | |
| --- | --- | --- | --- | --- |
| **SAMPLE SOURCE** | **T225 FLASK** | **TIME POINT** | **Packed RBC (vol)**  **(µL)** | **CULTURE SETUP TIME AND DATE** |
| **PRE-APHERESIS** | | | | |
| Pre-Apheresis | MFA culture T225 | T=4hr |  | __/Mar ; __:__ |
| Pre-Apheresis | MFA culture T225 | T=24hr |  | __/Mar ; __:__ |
| Pre-Apheresis | MFA culture T225 | T=48hr |  | __/Mar ; __:__ |
| **INTERMEDIATE** | | | | |
| Intermediate | MFA culture T225 | T=4hr |  | __/Mar ; __:__ |
| Intermediate | MFA culture T225 | T=24hr |  | __/Mar ; __:__ |
| Intermediate | MFA culture T225 | T=48hr |  | __/Mar ; __:__ |
| **FINAL BAG** | | | | |
| Final Bag | MFA culture T225 | T=24hr |  | __/Mar ; __:__ |
| Final Bag | MFA culture T225 | T=48hr |  | __/Mar ; __:__ |
| **WASTE BAG** | | | | |
| Waste Bag | MFA culture T225 | T=24hr |  | __/Mar ; __:__ |
| Waste Bag | MFA culture T225 | T=48hr |  | __/Mar ; __:__ |
| Note: Flasks with time point highlighted in red will be placed in the incubator in a vertical position | | | | |

| **Table 4. T75 Asexual/Sexual Culture Set-Up Times and pLDH Sampling** | | | | | | |  |
| --- | --- | --- | --- | --- | --- | --- | --- |
| **SAMPLE SOURCE** | **T75 FLASK** | **TIME POINT** | **Packed RBC (vol)**  **(µL)** | **CULTURE SETUP TIME AND DATE** | **pLDH (150µL RBC pellet) STORE -80°C** | **THIN SLIDE (10µL)** | |
| **PRE-APHERESIS** | **Not Applicable** | **T=0h** |  |  |  |  | |
| Pre-Apheresis | Asexual culture T75 | T=8h |  | __/Mar ; __:__ |  |  | |
| Pre-Apheresis | Asexual culture T75 | T=16h |  | __/Mar ; __:__ |  |  | |
| Pre-Apheresis | Asexual culture T75 | T=24h |  | __/Mar ; __:__ |  |  | |
| Pre-Apheresis | Asexual culture T75 | T=32h |  | __/Mar ; __:__ |  |  | |
| Pre-Apheresis | Asexual culture T75 | T=40h |  | __/Mar ; __:__ |  |  | |
| Pre-Apheresis | Asexual culture T75 | T=48h |  | __/Mar ; __:__ |  |  | |
| Pre-Apheresis | NAG-culture T75 | T=8h |  | __/Mar ; __:__ |  |  | |
| Pre-Apheresis | NAG-culture T75 | T=16h |  | __/Mar ; __:__ |  |  | |
| Pre-Apheresis | NAG-culture T75 | T=24h |  | __/Mar ; __:__ |  |  | |
| Pre-Apheresis | NAG-culture T75 | T=32h |  | __/Mar ; __:__ |  |  | |
| Pre-Apheresis | NAG-culture T75 | T=40h |  | __/Mar ; __:__ |  |  | |
| Pre-Apheresis | NAG-culture T75 | T=48h |  | __/Mar ; __:__ |  |  | |
| **INTERMEDIATE** | **Not Applicable** | **T=0h** |  |  |  |  | |
| Intermediate | Asexual culture T75 | T=24h |  | __/Mar ; __:__ |  |  | |
| Intermediate | Asexual culture T75 | T=48h |  | __/Mar ; __:__ |  |  | |
| Intermediate | NAG-culture T75 | T24h |  | __/Mar ; __:__ |  |  | |
| Intermediate | NAG-culture T75 | T=48h |  | __/Mar ; __:__ |  |  | |
| **FINAL BAG** | **Not Applicable** | **T=0h** |  |  |  |  | |
| Final Bag | Asexual culture T75 | T24h |  | __/Mar ; __:__ |  |  | |
| Final Bag | Asexual culture T75 | T=48h |  | __/Mar ; __:__ |  |  | |
| Final Bag | NAG-culture T75 | T24h |  | __/Mar ; __:__ |  |  | |
| Final Bag | NAG-culture T75 | T=48h |  | __/Mar ; __:__ |  |  | |
| **WASTE BAG** | **Not Applicable** | **T=0h** |  |  |  |  | |
| Waste Bag | Asexual culture T75 | T24h |  | __/Mar ; __:__ |  |  | |
| Waste Bag | Asexual culture T75 | T=48h |  | __/Mar ; __:__ |  |  | |
| Waste Bag | NAG-culture T75 | T24h |  | __/Mar ; __:__ |  |  | |
| Waste Bag | NAG-culture T75 | T=48h |  | __/Mar ; __:__ |  |  | |
| Note: Flasks with time point highlighted in red will be placed in the incubator in a vertical position | | | | | | | |

| **Table 12. T225 MFA Culture Sampling** | | | | | | | | | | | |  |
| --- | --- | --- | --- | --- | --- | --- | --- | --- | --- | --- | --- | --- |
| **Sample Source** | **Flask** | **Time Point** | **pRBC volume (µL)** | **Time to collect &**  **MFA** | **Actual Date and Time** | **Vol into Feed-Mix (650µL)** | **Thin Slide (10µL) Store - Ambient** | **Vol into DNA (100µL) Store -80°C** | **Vol into RNA (100µL) Store -80°C** | **Vol into Flow (50µL) Store 4°C** | **50% Media Change (Tick)** | **Initial** |
| Pre-Apheresis | MFA culture T225 | T=4hr |  | 13:30:00 | __/Mar ; __:__ |  |  |  |  |  |  |  |
| Intermediate | MFA culture T225 | T=4hr |  | 16:00:00 | __/Mar ; __:__ |  |  |  |  |  |  |  |
| Pre-Apheresis | MFA culture T225 | T=24hr |  | 9:30:00 | __/Mar ; __:__ |  |  |  |  |  |  |  |
| Intermediate | MFA culture T225 | T=24hr |  | 12:00:00 | __/Mar ; __:__ |  |  |  |  |  |  |  |
| Final Bag | MFA culture T225 | T=24hr |  | 14:00:00 | __/Mar ; __:__ |  |  |  |  |  |  |  |
| Waste Bag | MFA culture T225 | T=24hr |  | 14:00:00 | __/Mar ; __:__ |  |  |  |  |  |  |  |
| Pre-Apheresis | MFA culture T225 | T=48hr |  | 9:30:00 | __/Mar ; __:__ |  |  |  |  |  |  |  |
| Intermediate | MFA culture T225 | T=48hr |  | 12:00:00 | __/Mar ; __:__ |  |  |  |  |  |  |  |
| Final Bag | MFA culture T225 | T=48hr |  | 14:00:00 | __/Mar ; __:__ |  |  |  |  |  |  |  |
| Waste Bag | MFA culture T225 | T=48hr |  | 14:00:00 | __/Mar ; __:__ |  |  |  |  |  |  |  |

| **Table 13. T75 Asexual/Sexual Culture Sampling** | | | | | | | | | | | |
| --- | --- | --- | --- | --- | --- | --- | --- | --- | --- | --- | --- |
| **Sample Source** | **Flask** | **Time Point** | **Packed RBC volume (µL)** | **Time to harvest** | **Actual Date and Time** | **Pellet into pLDH (150µL) Store -80°C** | **Thin Slide (10µL) Store - Ambient** | **Pellet into DNA (100µL) Store -80°C** | **Pellet into RNA (100µL) Store -80°C** | **Pellet into Flow (50µL) Store -4°C** | **50% Media Change on Day 2(Tick)** |
| Pre-Apheresis | Asexual culture T75 | T=8hr |  | 12/03/2019 17:30 | __/Mar ; __:__ |  |  |  |  |  |  |
| Pre-Apheresis | NAG-culture T75 | T=8hr |  | 12/03/2019 17:30 | __/Mar ; __:__ |  |  |  |  |  |  |
| Pre-Apheresis | Asexual culture T75 | T=16hr |  | 13/03/2019 1:30 | __/Mar ; __:__ |  |  |  |  |  |  |
| Pre-Apheresis | NAG-culture T75 | T=16hr |  | 13/03/2019 1:30 | __/Mar ; __:__ |  |  |  |  |  |  |
| Pre-Apheresis | Asexual culture T75 | T=24hr |  | 13/03/2019 9:30 | __/Mar ; __:__ |  |  |  |  |  |  |
| Pre-Apheresis | NAG-culture T75 | T=24hr |  | 13/03/2019 9:30 | __/Mar ; __:__ |  |  |  |  |  |  |
| Intermediate | Asexual culture T75 | T=24hr |  | 13/03/2019 12:00 | __/Mar ; __:__ |  |  |  |  |  |  |
| Intermediate | NAG-culture T75 | T=24hr |  | 13/03/2019 12:00 | __/Mar ; __:__ |  |  |  |  |  |  |
| Final Bag | Asexual culture T75 | T=24hr |  | 13/03/2019 14:00 | __/Mar ; __:__ |  |  |  |  |  |  |
| Final Bag | NAG-culture T75 | T=24hr |  | 13/03/2019 14:00 | __/Mar ; __:__ |  |  |  |  |  |  |
| Waste Bag | Asexual culture T75 | T=24hr |  | 13/03/2019 14:00 | __/Mar ; __:__ |  |  |  |  |  |  |
| Waste Bag | NAG-culture T75 | T=24hr |  | 13/03/2019 14:00 | __/Mar ; __:__ |  |  |  |  |  |  |
| Pre-Apheresis | Asexual culture T75 | T=32hr |  | 13/03/2019 17:30 | __/Mar ; __:__ |  |  |  |  |  |  |
| Pre-Apheresis | NAG-culture T75 | T=32hr |  | 13/03/2019 17:30 | __/Mar ; __:__ |  |  |  |  |  |  |
| Pre-Apheresis | Asexual culture T75 | T=40hr |  | 14/03/2019 1:30 | __/Mar ; __:__ |  |  |  |  |  |  |
| Pre-Apheresis | NAG-culture T75 | T=40hr |  | 14/03/2019 1:30 | __/Mar ; __:__ |  |  |  |  |  |  |
| Pre-Apheresis | Asexual culture T75 | T=48hr |  | 14/03/2019 9:30 | __/Mar ; __:__ |  |  |  |  |  |  |
| Pre-Apheresis | NAG-culture T75 | T=48hr |  | 14/03/2019 9:30 | __/Mar ; __:__ |  |  |  |  |  |  |
| Intermediate | Asexual culture T75 | T=48hr |  | 14/03/2019 12:00 | __/Mar ; __:__ |  |  |  |  |  |  |
| Intermediate | NAG-culture T75 | T=48hr |  | 14/03/2019 12:00 | __/Mar ; __:__ |  |  |  |  |  |  |
