# Supplementary material for: Safety and feasibility of apheresis to harvest and concentrate parasites from subjects with induced blood stage Plasmodium vivax infection
Source: Malar J. 2021 Jan 14;20:43. doi: 10.1186/s12936-021-03581-w (PMC7807416; doi:10.1186/s12936-021-03581-w)
Supplement: Supplementary file 11 — Additional file 11. Spectra Optia Brochur System Overview. [file 12936_2021_3581_MOESM11_ESM.pdf]

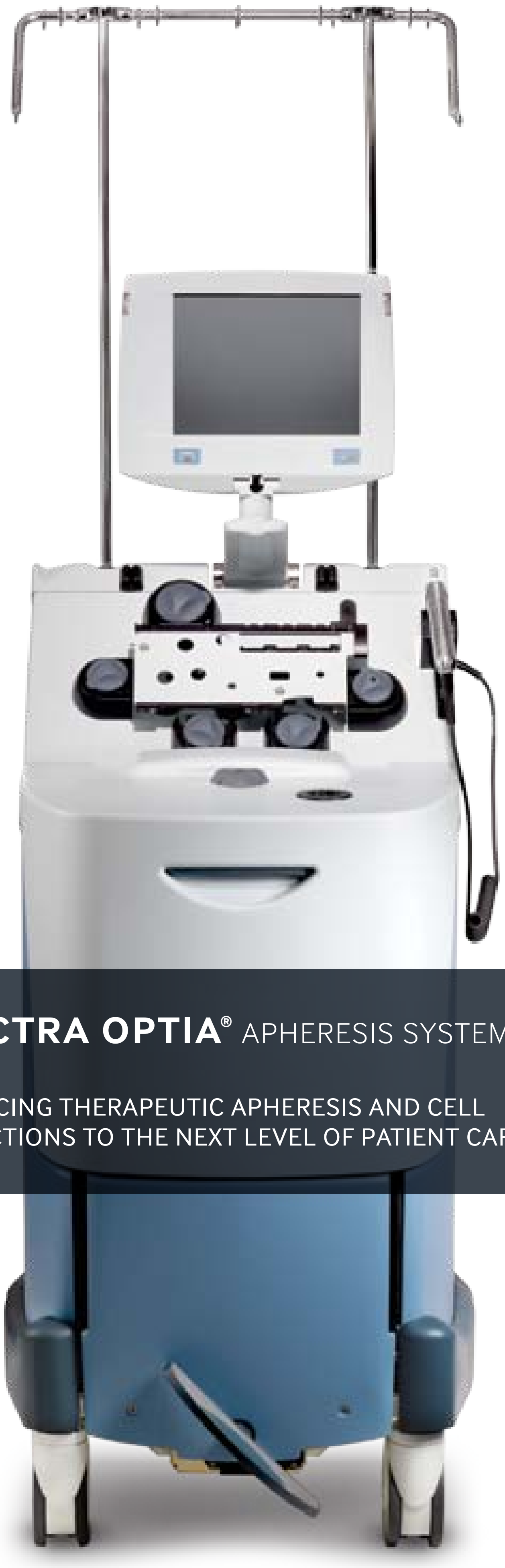

TERUMOBCT
Unlocking the Potential of Blood

SPECTRA OPTIA® APHERESIS SYSTEM

ADVANCING THERAPEUTIC APHERESIS AND CELL COLLECTIONS TO THE NEXT LEVEL OF PATIENT CARE

ADVANCED TECHNOLOGY THAT GIVES YOU CHOICE AND PRECISION

A SINGLE PLATFORM THAT DELIVERS UNPRECEDENTED VERSATILITY

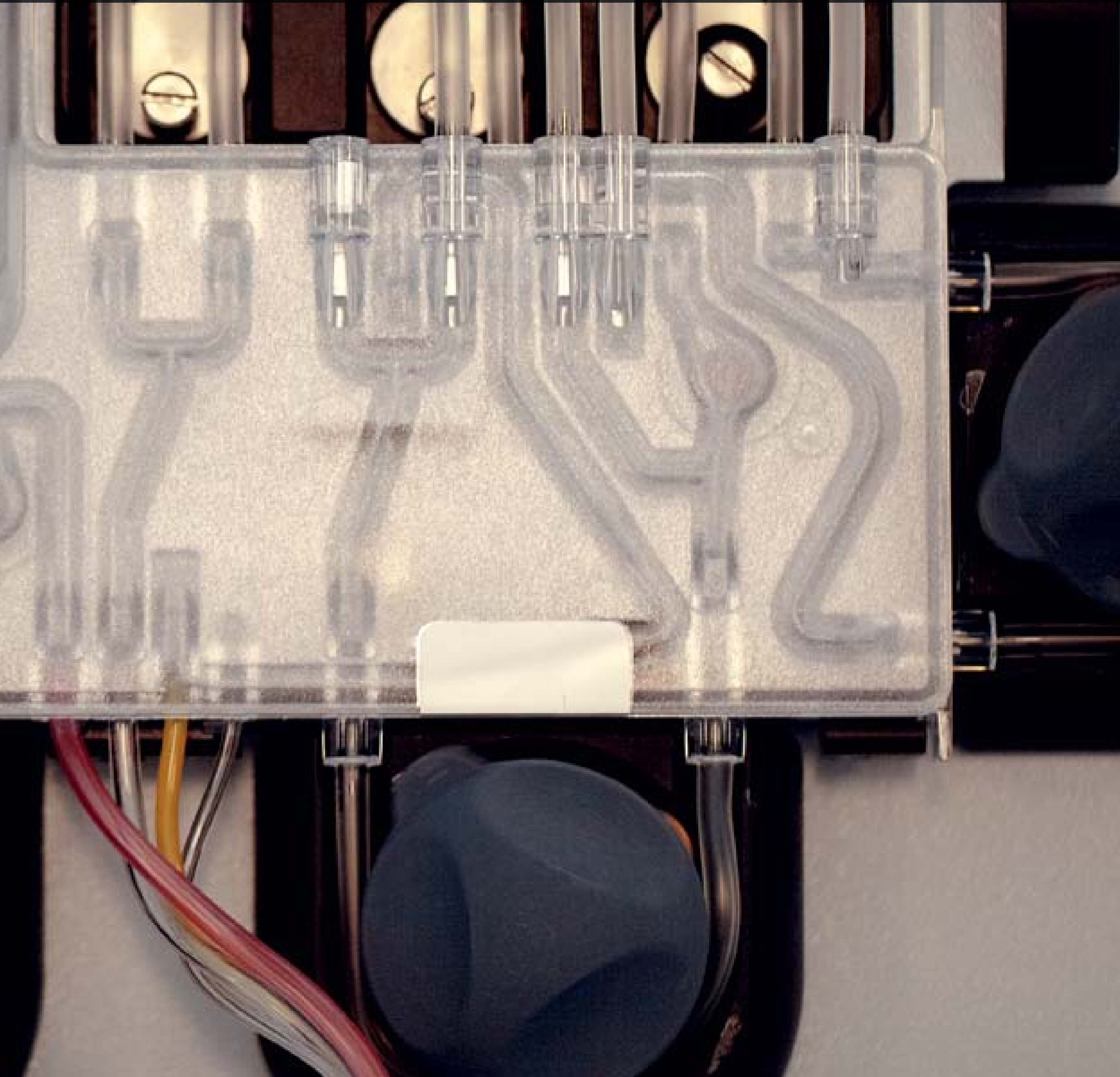

How it works

AUTOMATED INTERFACE MANAGEMENT (AIM) SYSTEM

Using a patented optical detection system, the AIM system continuously manages the separated layers, allowing the platelet and white blood cell layer to accumulate. AIM then directs the system to efficiently remove the targeted components.

- Automates interface adjustments without sacrificing your control
- Monitors and interprets the interface continuously so you don't have to rely solely on your vision
- Helps you efficiently remove targeted components
- Achieves highly predictable results through interface stability

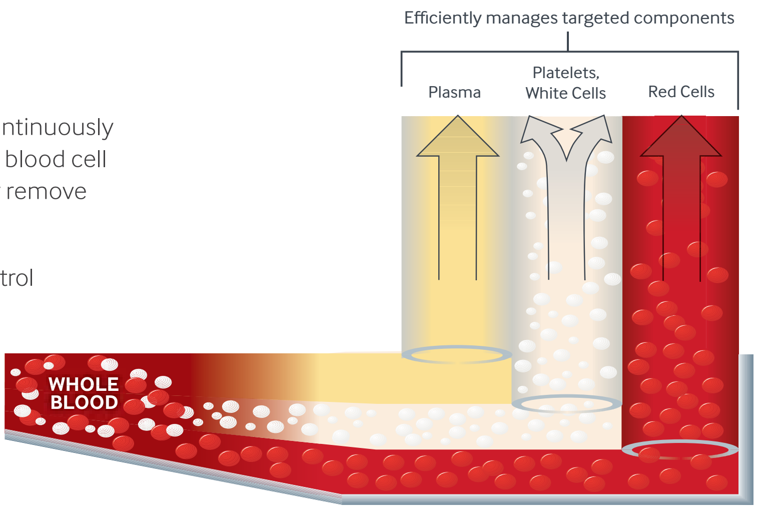

AIM continuously manages the interface

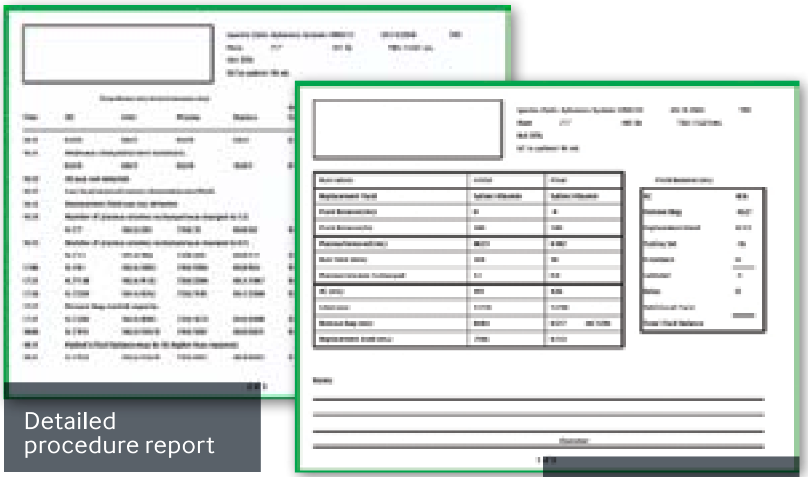

Detailed procedure report

Summarized procedure report

CONVENIENT DATA MANAGEMENT

- Prints reports or exports the data
- Captures and stores procedure data, reducing your need to manually transcribe data
- Helps you address regulatory requirements
- Tracks and records alarms during each of your procedures
- Gives you access to historical data, storing up to 100 procedures

FOCUS ON PATIENT COMFORT AND SAFETY

- Pump precision contributes to optimized fluid balance
- Low-volume tubing set accommodates smaller patients
- System verifies when you have the correct tubing set in place for the selected protocol
- Procedural automation enables you to spend more time with patients and donors
- Low operating noise adds to patient comfort

Working with you

Each and every interaction we have with you is important. By fostering open and ongoing relationships, we bring more value to you and the patients we're all focused on serving.

Even after the technology is in place, we continue to serve you with:

- Education and training
- Technical support
- Clinical and scientific support
- Customer support
- Users groups and professional networks

Intuitive graphical user interface (GUI)

- Connects you to the relevant information using a high-resolution, color touch screen
- Guides you through each step of the procedure
- Helps you enter the necessary patient and procedure information
- Gives you the right information at the right time to enhance procedure efficiencies
- Provides clear alarm messages that you can see at a glance

Incorporated Seal Safe System for sealing tubes

Ready-to-use tubing sets designed to handle multiple procedure types

- You can load the snap-in-place cassette in a few steps
- The system verifies when you have the correct tubing set in place for the selected procedure
- Low-volume tubing set accommodates your smaller patients
- With minimal set types to keep in inventory, you can achieve storage efficiencies

Highly maneuverable system

- Telescoping IV pole that you can easily raise or lower
- Folding screen to help you simplify moving and storage
- Large, durable wheels on pivoting casters brings you a high level of system maneuverability
- Advanced wheel pedal enables you to move or secure the system depending on your needs

Dimensions

- Weight: 91.6 kg (220 lbs)
- Height (lowered IV pole): 115.6 cm (45.5 in)
- Height (extended IV pole): 174 cm (68.5 in)
- Width: 52.7 cm (20.75 in)
- Depth: 81.3 cm (32.0 in)
- Floor space required: 0.43 m^2 (4.6 ft^2)

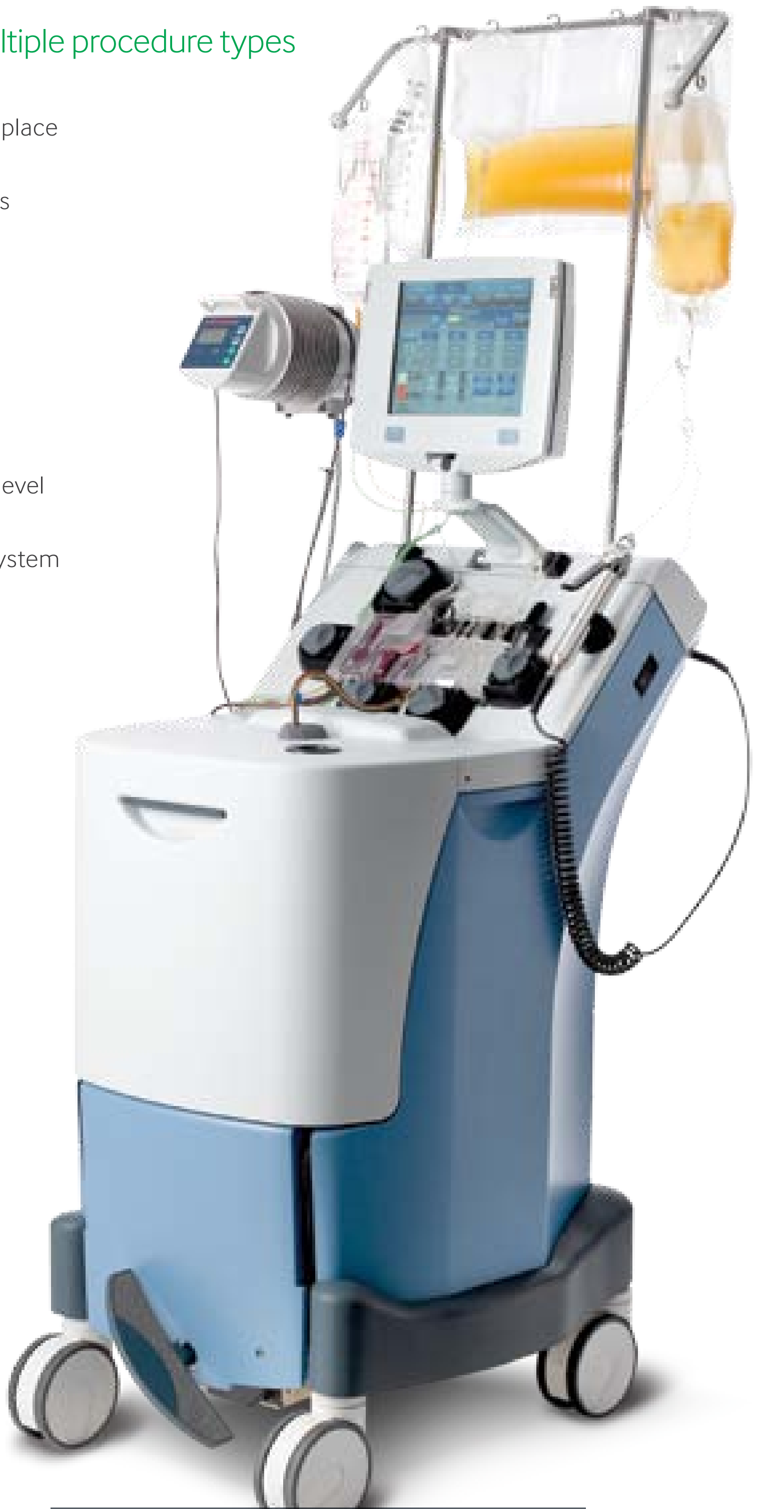

Shown with a tubing set for therapeutic plasma exchange procedures.

TERUMOBCT
Unlocking the Potential of Blood

Terumo BCT, Inc.

10811 West Collins Ave.
Lakewood, Colorado 80215-4440
USA

USA Phone: 1.877.339.4228
Phone: +1.303.231.4357
Fax: +1.303.542.5215

Terumo BCT (Asia Pacific) Ltd.

Room 3903-3903A, 39/F
ACE Tower, Windsor House
311 Gloucester Road
Causeway Bay, Hong Kong

Phone: +852.2283.0700
Fax: +852.2576.1311

Terumo BCT Europe N.V.

Europe, Middle East and Africa
Ikaroslaan 41
1930 Zaventem
Belgium

Phone: +32.2.715.05.90
Fax: +32.2.721.07.70

Terumo BCT Latin America

Juncal 1311 4th Floor
C1062ABO
Buenos Aires
Argentina

Phone: +54.11.5530.5200
Fax: +54.11.5530.5201

©2012 Terumo BCT, Inc. / PN 306670988

UNLOCKING THE POTENTIAL OF BLOOD | TERUMOBCT.COM
